# Screening Pathways through China, the Asia Pacific Region, the World

**DOI:** 10.3390/ijns5030026

**Published:** 2019-08-22

**Authors:** Veronica Wiley, Dianne Webster, Gerard Loeber

**Affiliations:** 1 The NSW Newborn Screening, The Children’s Hospital at Westmead, Westmead, NSW 2145, Australia; 2 Newborn Metabolic Screening Programme, LabPlus, Auckland City Hospital, Auckland 1140, New Zealand; 3 ISNS office, 3721 CK Bilthoven, The Netherlands

## 1. Introduction

The International Society for Neonatal Screening (ISNS) has met regularly at both international meetings and those of the various chapters. One such chapter is the Asia Pacific Region. This year we combine the 10th ISNS International Symposium and the 11th Asia Pacific Regional meeting in Hangzhou, China from 19th to 22nd September. The chosen theme for the meeting “Screening pathways through China, the Asia Pacific Region, the world” aims to elucidate the additional requirements of population screening beyond analytical aspects alone and includes sessions on pre-analytical, analytical and post-analytical requirements. The meeting begins with the end, incorporating a 1 day session on Monitoring and Evaluation of Newborn Screening Programs. Following the opening ceremony, we assess the current status of screening worldwide with presentations from each regional chapter. There are invited presentations on newborn screening systems and pathways to provide the groundwork for sessions assessing starting programs, increasing coverage and disorders included, as well as pathways incorporating molecular technologies. Expansion outside the laboratory includes assessing babies at the bedside and point of care testing for critical congenital heart disease has increasing coverage especially in the Asia Pacific region. As the possibilities for screening expand, it is timely to look into the future with gazing into the crystal ball of what might be.

The abstracts grouped as oral presentations and poster presentations are included below.

## 2. Oral Communications

### O01. 30 Years of Newborn Screening in China

ZhaoZhengyanThe Children’s Hospital Zhejiang University School of Medicine, Hangzhou, Zhejiang, China

China Newborn Screening Program started in 1981 when CH and PKU were initially screened in Shanghai. Technical specification for neonatal genetic metabolic diseases screening was released in 2004 and regulation for neonatal genetic metabolic disease was issued in 2009. The government pays more and more attention to the newborn screening. As a public health project in China, by the end of 2018, there has been 238 newborn screening centers in the China mainland and the screening rate has exceeded 97.5%. More than 15 million newborns have been screened for at least 3 diseases every year, which prevents tens of thousands of newborns from serious developmental delay and intellectual disability.

China newborn screening has established a complete network system, from sample collection and delivery, laboratory testing, results reporting, clinical diagnosis to follow-up. Strict management and quality control run through the whole work flow. At the same time, the information management system is constantly improving and updating. The newborn screening center establishes multi-channel communication with sample collection units and patients through the Internet, Alipay and WeChat platforms to improve management efficiency and parental satisfaction. In addition, the professional cold chain logistics system has also been introduced to newborn screening in recent years to ensure sample quality.

With the development of newborn screening, new technologies have been introduced and more screening disorders have been expanded. Tandem mass spectrometry screening of Inborn Error of Metabolism has been carried out in nearly 30 provinces across the country, with nearly 200 newborn screening centers participating by 2018. Zhejiang Newborn Screening Center has completed a 100,000 DMD screening pilot study and began the exploration of genome sequence-based screening for newborns.

With the realization of the importance of newborn screening, we also won the support of the government finance, the participation of non-profit organizations and social funds. Some drug for therapy are included into medical insurance and local salvation fund projects are founded to help affected babies, all of which have greatly promoted the development of China newborn screening.

### O02. Status of Newborn Screening in Latin America: Improvements and Difficulties

BorrajoGustavoDetección de Errores Congénitos, Fundación Bioquímica Argentina, La Plata, Argentina

Latin America (LA) is a region consisting of 20 countries characterized as presenting a wide diversity in terms of geography as well as demographics, ethnicity, economies and social and healthcare system. According to the World Bank, 3 Latin American countries have high-incomes, 16 have middle-incomes and the remaining one has low-incomes. As regards the Newborn Screening (NBS), it has been running for more than 30 years however a great heterogeneity has been observed among countries in the year and modality of NBS implementation, the panel of diseases screened for, the available technologies, the coverage reached, the legislation in force and the growth and success reached. Currently, Latin American countries can be grouped in five categories: a) with optimal NBS fulfillment, b) in an expansion and growth phase, c) in a recent implementation phase, d) with minimal and non-organized activities and e) without any NBS activities at all. Fifteen countries have national or regional NBS programs, however 2 of them have coverage < 3.0% and only 10/15 > 75%. In another country, NBS is conducted through the different components of its complex healthcare system. Thirteen countries have legislation in force defining NBS as mandatory. All countries that have national/regional programs (16) screen for CH, 11 for PKU, 9 for CAH, 6 for CF and galactosemia, 5 for biotinidase deficiency and hemoglobinopathies, 3 for MSUD and 1 for G6PDD. NBS by MS/MS is implemented at a national level only in 2 countries, while in other 4 it is conducted at a regional level, in the private sector or as a pilot. Despite these disparities, a sustained and significant growth in NBS activities has become evident during the last decade, highlighted by the implementation of new programs, the increase in coverage, the expansion of NBS panels and the increasing involvement of government and public health authorities. Nevertheless, still there are a lot of aspects to continue improving. Every year, around of 11 million infants are born in LA, having access to the NBS benefits around 70% of them.

### O03. Newborn Screening in North America

OjoduJeliliMillingtonDavidMeiJoanneAPHL, Silver Spring, MD, USA

Newborn Screening began in the United States of America in the 1960s and as of today all states perform screening for the majority of disorders on the federally issued Recommended Uniform Screening Panel (RUSP). In the United States there are 35 core conditions on the RUSP. Vermont, New York and Minnesota universally screen for all of these conditions. Massachusetts screens for all conditions but 3-MCC, MCAD and SMA are likely to be detected and reported due to universal screening of another disorder. Additionally, Pennsylvania screens for all conditions but BIOT, CF, GALT and SCID are offered to select populations. There are 26 secondary conditions on the RUSP.

The number of NBS disorders screened in Canada, as well as services provided, test methodologies and other procedures and requirements (i.e., bloodspot retention time, consent) differ based on province/territory. All ten provinces and three territories in Canada offer some kind of NBS: number of disorders screened range from five to perhaps 40. Newborn screening started in Prince Edward Island in 1963 (with PKU). A detailed breakdown of screening per province/territory can be found below.

For additional insight and background information, an environmental scan was conducted in 2011 regarding NBS for disorders and abnormalities in Canada, details of which will be further presented during the update [4].

^1^ https://cadth.ca/newborn-screening-disorders-and-abnormalities-canada

^2^ https://www.albertahealthservices.ca/info/Page9080.aspx

^3^ https://archive.news.gov.bc.ca/releases/news_releases_2009-2013/2009hls0041-000721.htm

^4^ http://www.perinatalservicesbc.ca/our-services/screening-programs/newborn-screening-program

### O04. Newborn Screening in Europe

LoeberJ. GerardISNS office, Bilthoven, The Netherlands

Over the last 50 years almost all European countries have introduced neonatal screening as an important public health feature. Depending on health care structure, available funds, local politics, input from professional groups and the general public, this introduction has led to different approaches in the way the screening programs have been set up, financed and governed. To get more information on these differences, in 2010 an online survey, commissioned by the EU, was compiled in which the whole screening program was covered by a questionnaire. This survey covered the EU member states, (potential) candidate member states and EFTA countries, in total 38 countries. Results showed large variations in the panel of screened conditions, ranging from 0 to more than 30 conditions; specimen collection time after birth; screening methodology; and storage of residual specimens, varying from 1–1000 years. In addition, confirmatory diagnostics, treatment and follow-up showed large discrepancies. In 2011 the project group provided a list of 60 recommendations to the EU Commission but so far none of them have been taken up. On the other hand, the EU has stimulated the formation of European Reference Networks (ERN’s) but these are focused mainly on the diagnosis and treatment of patients

Recently the same colleagues were asked to update their data. In some, mostly smaller, countries considerable changes have been implemented, mainly concerning the number of ms/ms detectable conditions. In contrast, in other mainly larger, countries very little has changed, if at all. Screening for SCID and CCHD, very much promoted and implemented in the USA is getting attention but so far only in pilot programs in a few countries. In contrast to the US, in Europe, national public health policies are either not at all, or only marginally, influenced by developments in neighboring countries. It is therefore unlikely that NBS programs in Europe will converge in the years to come.

ISNS has developed a searchable database showing the screening panels and additional information per jurisdiction worldwide. The database is freely available at https://membership.isns-neoscreening.org/disorders/. The contents may help policymakers to consider a strategy in their own jurisdiction for further expansion of the screening panel.

### O05. Newborn Screening in the Mena Region

KhneisserIssam^1^SkrinskaVictor^2^1University Saint Joseph, Newborn Screening laboratory, Beirut, Lebanon2Hamad Medical corporation, Metabolic Laboratory, Doha, Qatar

The Middle East and North Africa (MENA) is a region encompassing approximately 22 countries in the Middle East and North Africa, combined with its location between three continents, (Asia, Africa and Europe). The MENA region accounts for approximately 6% of the world’s population, 60% of the world’s oil reserves and 45% of the world’s natural gas reserves. Newborn Screening reached the region in the mid 80’s, mainly for CH, with the efforts of HVD and IAEA. During the years, countries like Saudi Arabia, Qatar and Lebanon did become regional and even international pioneer for newborn screening application by tandem mass spectrometry (KSA), homocystinuria and TREC-KREK screening (QATAR) and G6PD screening (Lebanon). Others lost their preliminary national program for CH—like Libya, Syria and Yemen—due to instability caused by man-made calamities. International efforts had been focusing to improve and organize the newborn screening in the region since 2006, with the Marrakech declaration. ISNS did provide some road map to the region since with fruitful results of the start National screening program in Morocco in 2015. The results of a survey query sent during the first quarter in 2019 will be presenting the current status of the MENA region where the high rate of consanguinity leads to an increase in the prevalence of these diseases, thus justify the cost-benefit of adding these screening.

### O06. Newborn Screening in The Asia-Pacific Region

RanieriEnzoHead Biochemical Genetics, Directorate of Genetics & Molecular Pathology, SA Pathology at the Women’s & Children’s Hospital Adelaide, South Australian, Australia; ISNS Board member representing the Asia-Pacific Region

Newborn screening in the Asia-Pacific region has continued to expand being led by Australia, New Zealand, Japan, Philippines, Taiwan, South Korea, P.R. China & Singapore. There have been significant advances in training and development in the region with groups from Singapore, Bangkok, Thailand, Shanghai, Beijing, P.R China, Kula Lumpur, Malaysia & Ho Chi Minh City, Vietnam either have undergone or are planning to undertake specialist training in Australia from the groups in Adelaide & Sydney. As examples, Singapore at the KKH Women’s & Children’s’ hospital Department of Pathology & Laboratory Medicine have been moving forward with the further expansion of their newborn screening program, planning to include congenital adrenal hyperplasia & severe combined immune deficiency. The Tu Du Children’s hospital in Ho Chi Minh City has a well-established program providing newborn screening to their hospital that has an annual delivery of over 60,000 babies. This group are planning to expand their program to include the screening for inborn errors of metabolism by MSMS. Specialist training will be sought from the department of Biochemical Genetics, Directorate of Genetics and Molecular Pathology at SA Pathology Women’s & Children’s hospital Adelaide, Australia. There is the establishment of a number of programs within India from groups notably in but not limited to, New Delhi, Chennai, Bangalore, & Mumbai either as pilot programs funded by the government sector or as private pathology providers. The P.R China has recently reported a neonatal screening coverage of their annual birth rate in excess of 98%. Well established programs exist throughout the country with centers of excellence located in Shanghai, Beijing, Guangzhou, Hangzhou and Shenzhen, with other provinces coming to the forefront in providing an expanded neonatal screening program.

There are either limited or no formal screening programs in Bhutan, Brunei, Burma, Cambodia, East Timor, Indonesia, Laos, Lebanon, Nepal, North Korea, Pakistan & Papua New Guinea. A portion of the Polynesian islands in the Pacific have access to neonatal screening through New Zealand.

There is the need to further improve access to developing countries in the implementation of fundamental screening programs for disorders such as PKU & congenital hypothyroidism. Whilst there is the availability of newborn screening testing provided by the private sector the cost to the individual may be prohibitive. Neonatal screening should be seen as a government initiative as an essential preventative public health program.

### O07. G6PD Newborn Screening Overview

KhneisserIssamUniversity Saint Joseph, Newborn Screening laboratory, Beirut, Lebanon

G6PD is a very common X-linked diseases. G6PD newborn screening possibility emerged with the Beutler method 40 years ago. Current ready to use kits contribute more for the consideration of adding this disease to the screening panel. Many studies showed the cost benefit of this screening by slashing the number of hemolytic crisis hospitalization in population with high risk of food habit. Others added that screening may prevent to kernicterus. Some recent data are in favor to add this screening in population at risk. More recent argument to be added, G6PD had been found to be the third cause of cerebral palsy in a developing country. In other area associated with man-made calamities, free food like fresh fava to refugees’ camps lead endemic chaotic hemolytic crisis. Huge number of people needed hospitalization and blood transfusion, with their condition to access to limited health facilities and related resources of blood bank.

Some may argue against the need to implement the need for the G6PD screening, taking into consideration all the above G6PD screening should be considered. G6PD is prevalent and the relative cost of adding this screening is penniless. On the other hand, the health education of the family and the patient is very efficient by reducing drastically the risk of hemolytic crisis.

### O08. Public Health Perspective of Screening

JacksonGaryCounties Manukau District Health Board, Auckland, New Zealand

Screening is an innately appealing prospect, with the potential to prevent the development of disease, prevent premature death and disability and to improve quality of life. However, it also has costs and causes harm. The aim of a good screening program should be to maximize benefits while minimizing the harm caused. Key elements for an organized screening program are clearly met for newborn screening. These include having an identified target population, disorders that are able to be tested for with high sensitivity and specificity and having facilities available for screening and testing. Being able to test is important but a number of other conditions need to be present, including strong quality control, training for all key personnel, a timely referral system for all positive results and facilities for the diagnosis and appropriate treatment of screening-detected disease, with ongoing follow-up. Systematic evaluation and monitoring of the whole program is required, particularly to ensure complete population coverage and the timely capture and treatment of all positive cases. Screening metrics from the New Zealand program are used to illustrate techniques for monitoring screening programs for rare disorders.

### O09. The Newborn Screening System

WebsterDianneNew Zealand Newborn Screening Program

All screening in public health is done to achieve health gains. In some jurisdictions these are quantified during the processes of adding disorders or program evaluation. For example, the aim of screening for congenital hypothyroidism is to enable affected children to reach their full growth and development potential and this obviously involves more than early detection. The processes involved in a newborn screening system are pre-analytical (informed consent, sample collection and transport); analytical (laboratory testing); post-analytical (reporting); short-term (from result reporting to diagnosis) and long-term (from diagnosis forever) follow-up; and program audit. The roles and organizations delivering screening interact at multiple points and in different ways—best shown diagrammatically—but, in summary

- Healthcare funders (not in all jurisdictions)—program policy, funding and audit
- Primary care providers—liaison with families, informed consent, sample collection and transport, receipt and action on results, liaison with secondary and tertiary care providers (e.g., specialist pediatricians) for diagnosis and treatment.
- Laboratory—testing and reporting results—screening testing, diagnostic and monitoring testing
- Follow-up team (may be in laboratory)—reporting results, liaison with maternity and pediatric care, tracking follow-up actions.
- Specialist pediatricians—contribution to screening policy, diagnostic testing, ongoing patient care, long-term follow-up.
- Families—consent for screening, ensuring results available, daily care of and delivering treatment to, affected children.

To achieve health gains for affected children screening programs must identify their local screening system and ensure effective communication between the components.

### O10. Newborn Screening Pathways

BonhamJim R.Department of Newborn Screening, Sheffield Children’s Hospital, Sheffield S10 2TH, UK

Families who participate in Newborn Screening do so to gain re-assurance about the well-being of their new baby. It therefore comes as a shock if anything goes wrong with the process or if a screen positive result is reported. It is well known that the resulting stress can have long lasting effects and in some cases can result in hypervigilance or anxiety within the family.

The best way to address this is to ensure that all aspects of the screening pathway are carefully planned and monitored and any incidents are viewed as an opportunity to learn and improve the organization and delivery of the program.

In this presentation we will examine the pathway beginning with societal awareness and the pre-screening information provided to parents. We will also examine the problems that can result from poor sample quality and the effects of disruption to sample transport before looking at the control of the analytical process itself.

We will also explore the various approaches made to the referral process and the way in which parents are contacted with screen positive results. The role and organization of confirmatory testing and the need to record and use outcome data to validate and develop the program will also be considered.

### O11. Starting Programmes: Comprehensive Newborn Screening in India—The Numbers Behind the Game

KapoorSeema^1^ThelmaB.K.^2^1Pediatrics, Division of Genetics & Metabolism, Maulana Azad Medical College, New Delhi, India2Department of Genetics, University of Delhi, India

It is envisaged that a holistic program to screen every newborn in the country has greater benefits in terms of reduction of both mortality and morbidity. The targets envisaged include blood spot screening for Congenital Hypothyroidism and Congenital Adrenal hyperplasia, Screening for critical congenital heart disease, screening for deafness and screening for hemoglobinopathies. The challenges for integrating these in a country like India include setting up of confirmatory labs, streamlining neonates with both deafness and critical congenital heart disease to appropriate measures and offering treatment and reproductive counseling for those with Thalassemia’s and hemoglobinopathies.

The problems envisaged are covering 51 births per minute in the capital alone, with many birthing facilities having 40–90 births per day. The time spent per baby in a comprehensive program which would include the pretest counseling, screening for hearing at an appropriate time before discharge in a relatively noise free environment, measuring saturations both pre ductal and post ductal in each neonate and collecting heel prick samples. Apart from the sheer numbers each paramedical health worker has to do, the optimum time frame with most neonates getting discharged at 24 hours will pose a critical challenge. For those undergoing repeat procedures at optimum time frames may move the discharge schedule leading to overcrowding in most facilities with the emergent scare of nosocomial sepsis.

Objective measures to correct these may be modification of optimum time frames but still reducing the number of false positives to improve the credibility of an emerging program. Using a single pulse oximetry cutoff, shifting heel prick collection to 16–24 hours could be some measures which may need serious consideration in a country like India. Handholding by most developed programs will go a long way in establishing a successful program.

### O12. Increasing Coverage—Improvement, Problems and Challenges

PadillaCarmencitaUniversity of the Philippines Manila, Newborn Screening Reference Center, National Institutes of Health, Ermita Manila, Philippines

Introduction: Newborn Screening (NBS) in the Philippines started in 1996. In 2018, it has screened 76.9% of its annual 2.056 million livebirths. In its 22 years of implementation, it has screened 12,233,971 yielding 4,335 positive cases for CH, 614 for CAH, 91 for PKU, -----131 for GAL, 196,160 for G6PD Deficiency. In 2012, MSUD was included in the panel, since then picking up 122 affected. In December 2014, expanded NBS was started and of 665,578 screened, 16 cases of fatty acid disorders, 16 cases of organic acid disorders, 4 cases of urea cycle defects and 480 cases with hemoglobin disorders were identified.

Purpose: This session reports on: 1) the improvements of the newborn screening program; 2) its current problems and 3) continuing challenges on country implementation.

Materials and Methods: Accomplishments reports for the past five years were reviewed to identify improvements made, screening coverage, challenges and plans.

Results and Discussion: The past five years saw major improvements in the program: 1) introduction of expanded NBS in 2014 and its full coverage in the national insurance in early 2019; 2) establishment of the long term follow-up program involving specialists in 14 of 17 political regions; 3) increased number of newborn screening laboratories; 4) setting up of metabolic and hemoglobinopathies reference laboratories and additional G6PD confirmatory laboratories; and Program 5) Addition of ASA in the newborn screening panel. Continuing challenges include 1) the shift to full expanded screening program; 2) home deliveries; 3) laboratory and quality assurance services, 4) cost of confirmatory tests 5) long term recall and management of patients

Conclusions: The DOH issued the strategic framework for the implementation of the Expanded Newborn Screening Program from 2017–2030 to provide policy direction and guidance for DOH offices, its attached agencies, local government units and development partners in prioritizing interventions for the health of newborns.

### O13. The Present Status of Congenital Hypothyroidism Screening in Bangladesh

Anwar-Ul-AzimMohammadHasanMizanulNational Institute of Nuclear Medicine and Allied Sciences (NINMAS), In Vitro, Dhaka, Bangladesh

Congenital Hypothyroidism (CH) is a serious condition of newborn babies, which leads to permanent mental and physical retardation if not identified and treated within the first few weeks of life. With present birth rate, every year about 3.4 million babies are born in Bangladesh and screening for CH is included in the “National Neonatal Health Strategy and Guidelines for Bangladesh, October 2009.” From 2006 to 2011, the government implemented a national project on CH screening and near about two hundred twenty thousand newborn babies were screened under that project. With that project, 96 newborn babies were diagnosed with CH and the incidence rate of CH was found 1:2300, which is much higher than world incidence (1:4000). Because of the high incidence rate of CH, a new national project “Screening of Congenital Hypothyroidism in newborn Babies (Phase 2)” has launched under the Annual Development Program (ADP) of Government. The three year (September 2018–August 2021) project is completely funded (5.66 million USD) by the Government. With the project, newborn screening program for the early detection of CH will be carried out throughout the country by Dried Blood Spot (DBS) method and the positive cases of CH will be treated for a long time. A dedicated newborn screening laboratory with radioimmunoassay and fluorescence immunoassay technology will be established at National Institute of Nuclear Medicine and Allied Sciences (NINMAS). Demographically, Bangladesh is an iodine deficient country and thyroid related diseases are common in our country. Hence, among the congenital disorders, Bangladesh is giving priority to the CH screening.

### O14. Newborn screening—An Indian Perspective

GuptaAshokAgrawalKamlesh KumarJ.K. Lone Hospital, Pediatrics, Jaipur, India

Newborn screening (NBS) aims at the earliest possible recognition of disorders to prevent the most serious consequences by timely intervention. Nowadays NBS program is an integral part of the health system of developed countries, whereas in India, it is still in the primitive stage.

Only few Indian states are running their own NBS program through discrete private and government sectors. Kerala has started universal screening in govt sector. Goa has re-launched the NBS under which babies were born at government-run hospitals are screened for 50 metabolic disorders. Delhi, Chandigarh and recently Tamil Nadu also have started NBS projects.

We started NBS program in J K Lon hospital, SMS medical college, Jaipur in Jan 2018. Our NBS panel consists of congenital hypothyroidism (CH), congenital adrenal hyperplasia (CAH) and glucose-6-phosphatedehydrogenase (G6PD) deficiency. Total 6476 newborns were screened over a period 14 months (Jan 2018 to Feb 2019). Prevalence of G6PD was 1in 720, of CAH 1in 3200 and none in CH.

ICMR project screened neonates in 5 Centers in India—Delhi, Mumbai, Chennai, Hyderabad and Kolkata (2007–2012). The incidence of CH was 1 in 1130 varying from 1 in 727 in Chennai to 1 in 1528 in Mumbai. The incidence of CAH overall was 1 in 5762 varying from 1 in 2036 in Chennai to 1 in 9983 in Mumbai. In Sir Ganga Ram Hospital Delhi NBS done for three disorders since 2006–CH, CAH and G-6-PD deficiency the incidence of CH was 1:1870, of CAH 1:4208 and for G6PD deficiency 1 in 263 in males and 1 in 1402 in females.

Conclusion: In resource limited countries like India NBS should be included in national program like National Health Mission to provide benefit to vast population.

**Keywords:** newborn; newborn screening program; congenital hypothyroidism (CH); congenital adrenal hyperplasia (CAH) and glucose-6-phosphatedehydrogenase (G6PD) deficiency

### O15. Newborn Screening for New Disorders in Developing Countries: Treatment Challenges

SkrinskaVictorKhneisserIssamHamad Medical corporation, Newborn Screening laboratory, Doha, Qatar

Ready-to-use kits were developed to enable the implementation of the neonatal screening of many new diseases. Newborn screening screened disease panel is expanding more around the world, reaching easily developing countries. Recently, the MENA region is witnessing an improvement in the field of neonatal screening, countries like Qatar, Lebanon started adding SCID. The first step to implement these screening is to assure the availability of Treatment. Multiple studies have shown that the success of Allogenic Hematopoietic Stem Cell Transplantation (HSCT) is highly dependent on the age of the patient: the earlier the better! This treatment is efficient also for mucopolysaccharidosis type IH (MPS I-H), also known as Hurler syndrome and X-ALD. Therefore, early screening of these diseases is of major interest, funding and the availability of the treatment should be taken into consideration with health policies and schemes.

Newborn screening is not buying a kit or an equipment, it is holistic approach with securing the means of access to treatment is the first and main goal.

### O16. Newborn Screening for Amino Acid Disorders, Organic Acidemias and Fatty Acid Oxidation Disorders Using Tandem Mass Spectrometry, Prevalence and Characteristics in China

GuXuefanXinhua Hospital, Shanghai Jiao Tong University School of Medicine, Shanghai Institute for Pediatric Research, Shanghai 200092, China

Newborn screening is a public health policy for the prevention of genetic diseases with significant preventive effects, with the best cost-effectiveness and the largest number of tests. Newborn screening in China began in 1981. After years of efforts, the coverage of congenital hypothyroidism and hyperphenylalaninemia screening has reached 97.5% in 2017.

China has started neonatal screening using tandem mass spectrometry in Shanghai since 2002 and is gradually popularizing in the country. In order to update the National Health Committee’s "Newborn Screening Technical Specification, 2010 Edition,” we propose to expand the list of treatable and preventable diseases. For this purpose, we conducted a national survey on the status of neonatal screening, diagnosis and treatment on amino acid disorders, organic acidemias and fatty acid oxidation disorders. This work collected the data from the beginning of the mass spectrometry screening to the end of 2017. A total of 49 newborn screening centers from Zhejiang, Shanghai, Henan, Shandong, Guangxi, Fujian and other provinces were collected, the maximum single-center screening was 2.4 million. Seventeen screening centers with a screening volume of less than 30,000 were excluded (123,617 in total) and a total of 7,819,662 in 32 screening centers were included in the statistics.

The results showed that 2480 patients were detected by tandem mass spectrometry in total 7,942,829 newborns. The prevalence rate was 1/3311 (1/1366–1/7696). A total of 36 diseases were found. The top 10 diseases with the highest prevalence include hyperphenylalaninemia (1/10,712), methylmalonic acidemia (1/15,213), primary carnitine deficiency (1/24,513), short-chain acyl-CoA dehydrogenase deficiency (1/56,257), 3-methylcrotonyl-CoA carboxylase deficiency (1/62,557), citrullinemia type II (1/68,594), hypermethionemia (1/104,262), medium chain acyl-CoA hydrogenase deficiency (1/150,378), propionic acidemia (1/195,492), isovaleric acidemia (1/195,492). However, some disease spectrum and prevalence are quite different in southern Chinese and northern Chinese, for example, hyperphenylalaninemia and methylmalonic acidemia, the incidence rate in the northern is higher than in the southern Chinese and for citrullinemia type II, the incidence rate in the southern is higher than in the northern Chinese in general.

According to the principle of neonatal screening for disease and disease spectrum in Chinese, our expert committee have selected 12 diseases as the preferred screening panel among the amino acid, organic acid and fatty acid oxidation disorders detected by the tandem mass spectrometry technology: hyperphenylalaninemia, methylmalonic acidemia, primary carnitine deficiency, citrullinemia type II, medium chain acyl CoA dehydrogenase deficiency, propionic acidemia, isovaleric acidemia, glutaric acidemia type I, maple syrup urine disease, long chain hydroxyacyl CoA dehydrogenase deficiency, citrullinemia type I, homocystinuria.

As a big country with 17 million newborns per year, the economic development and medical level are quite different. There are still problems such as insufficient diagnosis ability of genetic metabolic diseases, lack of resources for treatment experts, lack of necessary therapeutic drugs and perfect social security system. Government policy support and further personnel training are urgent.

### O17. Challenge of Genotyping all Detected Cases in NBS in Japan—Genetic Diagnosis Has Been Provided For 260 Patients with Inherited Metabolic Diseases Positively Screened by NBS in Japan since 2014

T.Fukao^1^,^2^H.Sasai^1^,^2^Y.Ago^1^H.Matsumoto^1^H.Otsuka^1^,^2^,^3^J.Hosokawa^4^R.Fujiki^4^O.Ohara^4^Y.Nakajima^5^T.Ito^5^K.Hara^6^M.Kobayashi^7^G.Tajima^8^O.Sakamoto^9^J.Kido^10^S.Matsumoto^10^K.Nakamura^10^T.Hamazaki^11^H.Kobayashi^12^Y.Hasegawa^12^1Dept. of Pediatrics, Gifu Univ, Gifu, Japan2Div. of Clin Genet, Gifu Univ Hosp, Gifu, Japan3Dept. of Neonat Med, Gifu Pref Gen Med Center, Gifu, Japan4Kazusa DNA Res Inst, Kisarazu, Japan5Dept. of Pediatr, Fujita Medical Univ, Toyoake, Japan6Dept. of Pediatr, Kure Med Center, Kure, Japan7Dept. of Pediatr, Jikei Univ Med, Tokyo, Japan8Div. of Neonatal Screening, NCCHD, Tokyo, Japan9Dept. of Pediatr, Tohoku Univ, Sendai, Japan10Dept. of Pediatr, Kumamoto Univ, Kumamoto, Japan11Dept. of Pediatr, Osaka City Univ, Osaka, Japan12Dept. of Pediatr, Shimane Univ, Izumo, Japan

Newborn screening (NBS) using tandem mass started all over Japan in 2014. Since them the number of NBS-target diseases increased from 6 to 20. We have been conducting a follow-up project of patients with inherited metabolic diseases (IMDs), who were positively screened in NBS, after confirming their genotypes since 2014. We designed a gene panel covering the NBS-target IMDs and their related diseases. Gene panel analysis was performed at Kazusa DNA Research Institute. In 2014–2016, mutation analysis was done on research basis and after 2017 mutation analysis for a large part of NBS-target IMDs were covered by public medical insurance. More than 340 cases were enrolled in this gene panel study for five years (January 2014 to March 2019). Among them, the number of patients with IMDs detected by NBS were 260, as follows—Propionic acidemia (49), Hyperphenylalaninemia (37), Methylcrotonylglycinuria (27), VLCAD deficiency (19), Methylmalonic acidemia (18), Galactosemia (17), MCAD deficiency (16), Maple syrup urine disease (14) and so forth. Most of other 70 cases were symptomatic patients who were previously diagnosed. In some NBS-target IMDs, we found specific mutations only identified in NBS-positive cases. For example, Y435C in *PCCB* was predominantly identified in Japanese patients with propionic acidemia who were detected in NBS. Clinical phenotype of patients with Y435C has been asymptomatic or mild. Similar findings were also seen in VLCAD deficiency. It is challenging to distinguish patients with high risk from those with no symptom or low risk and make genotype-adjusted clinical guidelines.

### O18. Next Generation Sequencing in Newborn Screening in the United Kingdom National Health Service

ThomasRebeccavan CampenJuliaSollarsElizabethBartlettClareDaweJenniferWinshipPeterKirkRichardBonhamJimGoodeveAnneDaltonAnnSheffield Children’s NHS Foundation Trust, Sheffield Diagnostic Genetics Service, Sheffield, UK

Next Generation DNA Sequencing (NGS) has the potential to improve the diagnostic and prognostic utility of newborn screening programs. DNA suitable for NGS can be prepared from dried blood spots taken at birth. The genetic information is independent of the baby’s age, condition, feeding or gestation. NGS could be used in newborn screening as a first line test for genetic disorders with no biochemical marker or as an adjunct test to improve a prognosis, due to particular genetic variants being associated with variations of a disease. This study aims to assess the feasibility of screening dried blood spots using Next Generation Sequencing in a National Health Service (NHS) laboratory. An automated high throughput NGS protocol was designed and optimized using dried blood spots collected from healthy controls. An NGS panel targeting five genes relevant to newborn screening disorders was validated using commercial cell lines and comparing results from venous blood and dried blood spots from matched samples. The technical sensitivity of the assay was 100% and technical specificity was 99.96%. Overall, seven high throughput NGS runs were performed to ascertain the reproducibility of results. This NGS protocol enables processing of up to 1000 sample a week with a turnaround time of four days from receipt of sample to reporting. This study concludes that it is feasible to automate targeted NGS on routine dried blood spot samples in a UK NHS laboratory setting.

### O19. A Cohort Study of 300 Chinese Patients with Isolated Methylmalonic Aciduria

KangLuluLiuYupengLiuYiHeRuxuanJinYingLiMengqiuSongJinqingZhangYaoDongHuiLiuXueqinYanHuiZhangChunyuXiaoHuijieLiHaixiaZhangHongyunQinJiongZhengHongChenYongxingLiDongxiaoWeiHaiyanZhangHuifengZhangYananDengXinLiXiaowenHuangMinLiPeiZhaoZijiangYangYanlingPeking University First Hospital, Department of pediatrics, Beijing, China

Background: Methylmalonic aciduria is the most common organic aciduria in China. This cohort study aimed to characterize epidemiology, genotypic and phenotypic variability in Chinese patients with isolated methylmalonic aciduria.

Method: Three hundred cases of isolated methylmalonic aciduria who were referred to Peking University First Hospital from 1998 to 2018. 129 (43.0%) patients were females and 171 (57.0%) were males. The diagnosis is based on clinical examination, biochemical assays and genetic analysis.

Results: 300 patients were diagnosed as isolated methylmalonic aciduria at the age of 3 days to 23 years, with the mean age of 1.01±0.15 years. 58 patients (19.3%) were detected by newborn screening. 242 patients (80.7%) were clinically diagnosed after onset. of 234 patients for whom clinical data were available, 93 cases (38.3%) manifested with brain damage. 73 (30.0%) exhibited hematological abnormality. 116 (47.7%) had metabolic acidosis, vomiting, lethargy, diarrhea, hyperpnea and coma. 20 cases accompanied with renal involvement (8.23%). 19 cases had skin lesion (7.82%). Pathogenic mutations were found in five gene (*MUT*, *MMAA*, *MMAB*, *SUCLG1*, *SUCLA2*) associated with isolated methylmalonic aciduria from 281 cases. 114 mutations were detected. 58 patients who were detected by newborn screening got favorable outcome after the treatment by cobalamin, L-carnitine and dietary intervention. But various sequelae were observed in the patients who were diagnosed after onset. Eleven patients were improved after liver transplantation.

Conclusion: Neonatal screening is the key for the early treatment of the patients with isolated methylmalonic aciduria. Our study expanded the spectrum of phenotype and genotype of Chinese patients with isolated methylmalonic aciduria.

### O20. High-Throughput Identification of Hemoglobinopathies & Thalassemias by HRAM/MS

LukacsZoltanMechtlerThomasSchwarzMarkusKasperDavid C.WiesingerThomasHamburg University Medical Center, Newborn Screening and Metabolic Diagnostics Unit, Hamburg, Germany

The early diagnosis of hemoglobin disorders becomes more and more important due to higher numbers of affected individuals in the European Union, especially in urban areas (e.g. recent studies showed an incidence of ca. 1 in 2500 newborns in Hamburg and Berlin). In general, genetic disorders related to hemoglobin (Hb) can be classified into two main groups (i) hemoglobinopathies and (ii) thalassemias. Early identification of Hb S (SCD) in homozygous form is critical for preventive therapy. In addition, Hb variants (e.g., Hb -C, -D and -E) with co-inherited Hb S in a heterozygous form are also of importance in regard to early detection and therapy. Due to single mutations in the beta chain, these variants may differ only by 1 Dalton resulting in issues for the resolution when traditional tandem mass spectrometry is applied, especially for ion species that demonstrate high *m*/*z* ratios (>15+). To tackle this challenge, a novel approach using high resolution mass spectrometry was evaluated. For this purpose, intact Hb chains and the corresponding tryptic digest were mixed in a defined ratio and the mixture was directly analyzed by mass spectrometry. Suitability and accuracy of the novel method were confirmed by preliminary experiments and have been compared to conventional HPLC analysis (Bio-Rad, Munich, Germany).

### O21. Blood Collection on Filter Paper for Newborn Screening Program—Updates to the CLSI Standard NBS01, 7th Edition

MeiJoanne^1^BorrajoGustavo^2^De JesúsVictor^1^1Division of Laboratory Sciences, National Center for Environmental Health, Centers for Disease Control and Prevention, Atlanta, GA 30341, USA2Fundación Bioquímica Argentina, La Plata, Argentina

Background**:** The Clinical and Laboratory Standards Institute (CLSI) routinely develops and updates guidelines and standards for clinical testing. Since 1982, NBS01 has been used to inform and instruct health care professionals, who collect and submit DBS specimens for NBS, manufacturers who develop testing methodologies and equipment and NBS programs that perform testing.

Methods**:** The Document Development Committee, consisting of an international group of NBS professionals experts representing several countries met to discuss changes that occurred since the publishing of the approved standard NBS01-A6 in 2013. Several chapters and appendixes were revised and included reformatting the standard to follow a sequential process.

Results**:** Changes made in the 7^th^ edition include: easy-to-follow step-action tables; definitions for check sum character, expected range, false-negative screening test result, false-positive screening test result, in-range result, out-of-range result and re-collection; revised appendices for collecting an acceptable blood spot specimen, specimen quality and the protocol for testing absorption characteristics of filter paper; and extension of the shelf-life of filter paper from three to five years.

Conclusions: NBS01, 7th edition updates the procedures, challenges and related topics associated with specimen collection, the filter paper section of the specimen collection device and the application of blood onto filter paper for NBS analyses, with the goal of better serving the global community and harmonizing the collection and application of high-quality DBS specimens for NBS worldwide. Standardizing these procedures within and across NBS programs and systems is expected to improve NBS consistency and efficacy.

### O22. Cost Utility of a Proposed Additional Disorder

JacksonGaryCounties Manukau District Health Board, Auckland, New Zealand

Correctly developed newborn screening programs are one of the most cost-effective interventions available to modern medicine. However, there are hundreds of metabolic disorders. Even with an established newborn program and an accurate test available, it may not be appropriate or cost-effective to add the test to the program. For example, it may not be possible to treat the disorder in the early newborn period. This presentation examines the process to determine the cost-utility of adding a new disorder to a screening program. The case for adding SCID (Severe combined immunodeficiency) screening to the New Zealand program is used as a worked example. SCID is a rare inherited disorder caused by a deficiency or absence of T cells, a type of lymphocyte that plays a central role in immunity. SCID is difficult to diagnose clinically and without treatment most babies die before the age of one. Early detection of SCID is associated with improved treatment outcomes and lower health system costs.

### O23. Screening for Spinal Muscular Atrophy (SMA) in NSW, Australia

WileyVeronicaEsberRoslynKimWon-TaeWottonTiffanyFarrarMichelleSydney Children’s Hospital Network, NSW Newborn Screening Program, Westmead, Australia

Spinal muscular atrophy (SMA) is an autosomal recessive genetic neuromuscular disorder, producing significant disability and mortality which affects ~1:10,000 births. There are four types of SMA which are categorized based on age of onset and achieved motor function. In its severest and most frequent form it causes progressive infantile paralysis, respiratory failure and premature death before two years of age. The disorder is caused by insufficient levels of the survival motor neuron protein, caused by mutations in the survival motor neuron 1 (SMN1) gene and modulated by the copy-number of a paralog, SMN2. Several treatment modalities have been investigated recently. Natural history data suggests that the pre-symptomatic window may be brief (several weeks or months). Importantly the benefit of early diagnosis and initiation of treatment on outcome is now recognized, with the most beneficial response to treatment to date seen in patients treated before the onset of symptoms. Given this timescale, NBS presents as the optimum and acceptable screening tool.

Since August 2018 there has been a 2 year pilot program which aims to demonstrate the feasibility, sensitivity and specificity of treated outcome of SMA in NSW, Australia. Samples collected for routine newborn bloodspot screening are also being tested using a DNA-based multiplex qPCR assay for homozygous SMN1 deletion to detect infants at risk of developing SMA. For positive samples a droplet digital PCR assay is used to determine copy number of SMN2.

In 7 months, there have been 58,640 babies screened with 4 babies referred for diagnostic testing where all were proven to have the disorder with 2 or 3 copies of SMN2. All proven cases were successfully treated before 4 weeks of age.

Inclusion of SMA in routine newborn screening will depend on the longer term outcome of treated cases.

### O24. Validation of a Multiplexed Real-time PCR Assay to Detect SCID and SMN1 Homozygous Exon 7 Deletion and Droplet Digital PCR Assay to Assess SMN2 Copy Numbers in Newborn Screening for Spinal Muscular Atrophy

BakerMeiMochalSeanLoeheMandieZeitlerBethanyChenJinZhangRenJohnstoneAlecNewPannSwobodaKathrynUniversity of Wisconsin School of Medicine and Public Health, Wisconsin State Laboratory of Hygiene, Madison, WI 53706, USA

Spinal muscular atrophy (SMA) is one of the most common lethal recessive genetic conditions, with an incidence of 1 in 11,000 births. The condition is associated with significant motor disability, respiratory and nutritional compromise and death in infancy or childhood in more than 50% of affected children. Infants with homozygous deletion of SMN1 and 2 SMN2 copies typically manifest the most severe infantile form of SMA, SMA type I. However, symptom onset and disease severity in infants and children with 3 or more copies of SMN2 is more variable.

We successfully developed and validated a real-time PCR assay to simultaneously screen for SMA and severe combined immunodeficiency (SCID). The PCR assay identifies the absence of exon 7 in the SMN1 gene while simultaneously evaluating the copy number of the T-cell receptor excision circles (TREC). Moreover, we have further developed and validated a droplet digital PCR (ddPCR) assay to assess the SMN2 copy numbers. We used blood spots from a well-characterized cohort of subjects with SMA, SMA carriers and control subjects enrolled in the Newborn Screening Translational Research Network (NBSTRN) Longitudinal Pediatric Data Resource (LDPR) study. Subjects were diagnosed with type 0, I, II, III or IV SMA and had 2, 3, 4 or more than 4 SMN2 copies. Samples from these subjects were used to validate the accuracy, reproducibility and clinical validity of this assay.

Because SMN2 copy number is a major modifier of SMA disease where the higher SMN2 copy numbers are associated with later onset and milder phenotype, identifying infants with 2 or 3 SMN2 copies and treating at the earliest possible timepoint is critical to ensure the best outcomes. The comprehensive approach of NBS for SMA, that includes “just in time” knowledge of SMN2 copy numbers in newborns who have homozygous deletion of exon 7 in SMN1, will facilitate early clinical follow-up, family counseling and treatment planning.

### O25. Newborn Screening for Severe Combined Immunodeficiency in California, 2010–2017

CurrierRobertAmatuniGeorgeNaidesStanleySciortinoStanleyPuckJenniferUniversity of California, San Francisco, Pediatrics, San Francisco, CA 94143, USA

Objectives: Since newborn screening for severe combined immunodeficiency (SCID) was instituted in California in 2010, over 3.25 million infants in the state had DNA from dried blood spots assayed for T-cell receptor excision circles (TRECs). Abnormal TREC results were followed up with liquid blood flow cytometry for T-cell abnormalities to determine urgency and appropriate site for further evaluation and care. We report the performance of the SCID screening program, outcomes of infants who were identified and reference intervals for lymphocyte subsets in neonates.

Methods: Data that were analyzed included demographics, nursery summaries, TREC and lymphocyte flow-cytometry values and available follow-up, including clinical and genetic diagnoses, treatments and outcomes. Flow-cytometry data from infants were analyzed separately for strata of birth weight or effective gestational age at testing (EGA), defined as gestational age at birth plus age at testing.

Results: Infants with clinically significant T-cell lymphopenia (TCL) were successfully identified at a rate of 1 in 15,300 births. of these, 50 cases of SCID or 1 in 65,000 births (95% confidence interval 1 in 51,000–1 in 90,000) were found. Prompt treatment led to 94% survival. Although no cases of typical SCID are known to have been missed, 2 infants with delayed-onset leaky SCID had normal neonatal TREC screens but came to clinical attention at 7 and 23 months of age.

Reference intervals were generated for absolute numbers and percentages of lymphocyte subsets from infants with EGA 22–52 weeks. Sex and ethnicity were not significant determinants of lymphocyte subset counts in this population after controlling for EGA or birth weight. Lymphocyte counts rose postnatally.

Conclusions: Newborn TREC screening, although unable to detect immune defects in which T-cells are present, is effective for identifying SCID and clinically important TCL with high sensitivity and specificity. The experience in California supports the rapid adoption of SCID newborn screening.

### O26. Newborn Screening for Neonatal Intrahepatic Cholestasis Caused by Citrin Deficiency and Molecular Characterization of SLC25A13 Gene in Guangzhou Population

TangChengfangFengYiXuWeiLiuSichiJiangXiangTangFangLiNaHuangYonglanGuangzhou Women and Children’s Medical Center, Guangzhou Newborn Screening Center, Guangzhou, China

Objective To investigate the sensitivity of newborn screening for neonatal intrahepatic cholestasis caused by citrin deficiency (NICCD) based on tandem mass spectrometry and the carrying rate of known pathogenic variants of SLC25A13 in Guangzhou population.

Methods A total of 124,250 neonates born in Guangzhou from January 1,2015 to December 31, 2018 were screened for NICCD using tandem mass spectrometry technology. SLC25A13 mutation analysis was performed in high-risk newborn and outpatients suspected NICCD. We retrospective analyzed the pathogenic variants of SLC25A13 in 2395 healthy children who were performed the whole exon sequencing as controls.

Results Among the 124,250 screened neonates, 31 were positive for NICCD screening and one of them was confirmed. We found three patients with NICCD were false negative in this newborn screening program. All of the four patients were homozygous for 851del4/851del4. Among 2395 healthy children, we detected 60 alleles with reported pathogenic variants of SLC25A13 and the carrying rate of pathogenic allele was 1/40. The estimated prevalence of citrin deficiency is about 1/6400. The most common pathogenic variant was c.851_854del (56.7%) and second was c.790G>A (23.3%). The controversial variant c.2T>C was found in 113 children with heterozygous and 2 with homozygous and the carrying rate of c.2T>C was 1/21.

Conclusion The sensitivity of newborn screening for NICCD by tandem mass spectrometry is limited. It is necessary to reevaluation blood amino acid profile in suspected early infants with negative newborn screening results. The carrying rate of pathogenic variants of SLC25A13 in Guangzhou population is 1/40 and the estimated prevalence of Citrin deficiency is 1/6400.

**Keywords:** citrin deficiency; newborn screening; *SLC25A13* gene

### O27. Newborn Screening for DMD

TongFanThe Children’s Hospital Zhejiang University School of Medicine, Department of genetics and metabolism, Hangzhou, China

Objectives To investigate the epidemiology, genotype and phenotype of male neonatal DMD in Zhejiang province of China.

Methods All male newborns with the informed consent of their parents were enrolled from July 16, 2018 to August 30, 2018 in Zhejiang Newborn Screening Center. Above 400ng/ml of CKMM (dried blood sample) as the positive screening outcome, which with CK (venous sample) ≥ 700U/L or continue elevated cases did genetic tests. All confirmed and suspected DMD were followed up in the congenital myopathy clinic every 1-6 months.

Results A total of 53,166 male neonates were screened, among them, 8 cases with pathogenic variants of *DMD* (6 cases with deletion, 1 case with duplication, 1 case with nonsense mutation p.E3077 *) and 8 cases with other types of congenital myopathy (including *MYH7, CAV3, RYR1, LDB3, CLCN1, CACNA1S, PLEKHG5, GAA*). The de novo variants accounted for 42.8% of *DMD* (3/7). The prevalence of DMD was 1:6645. Two cases with benign variants of *DMD* were detected (p.Q1835L, c.5923-11A>T). CK increased gradually during the follow-up of all DMD cases, no MRI changes were found in thigh muscle and sural muscle until 6-8 months and the ASQ score was normal. The case with *LDB3* p.Q598H variant were found to have dysphagia, recurrent respiratory infection and global develop delay.

Conclusion DMD is common in Zhejiang population. Newborn screening is beneficial to family genetic counseling and improves the outcome of DMD. The p.Q1835L and c.5923-11A>T of *DMD* was confirmed to be benign variants and the p.Q598H of *LDB3* was suspected to be pathogenic variant.

### O28. Tandem Mass Spectrometry for Newborn Screening of Lysosomal Storage Disorders and Beyond

GelbMichaelUniversity of Washington, Chemistry and Biochemistry, Seattle, USA

Newborn screening and diagnosis of Lysosomal Storage Disorders (LSD) by use of tandem mass spectrometry (MSMS) has been in place for over 15 years. LSD newborn screening started in Taiwan for Pompe disease. This was achieved through the use of acarbose (Gelb et al.) that enabled the selective measurement of the alpha-glucosidase.

Currently, both Pompe and MPS-I are on the Recommended Uniform Screening Panel (RUSP) in the USA and have been included into routine screening programs in several states in the USA, in parts of Italy and in Taiwan. We have compiled newborn screening performance data from these laboratories and the results show rates of first-tier, screen positives (low enzyme activity below a set cutoff) of 10-20 per 100,000 newborns across all laboratories. Some programs include the screening for other LSDs namely Gaucher, Krabbe, Niemann-Pick-A/B and Fabry diseases that can be multiplexed with Pompe and MPS-I.

More recently, Taiwan and the US Washington state have undertaken prospective pilot studies for additional LSDs (MPS-II, MPS-IIIB, MPS-IVA, MPS-VI, MPS-VII and MLD), the results of which show acceptable false positive rates (~10 per 100,000). The MPSs are screened by enzymatic activity assay, whereas MLD requires a substrate accumulation analysis because of the high prevalence of pseudo deficiency alleles identified by low enzyme activity. These programs have shown the feasibility of newborn screening of these LSD by use of MSMS technology.

In the past several months we have expanded the disorders that can be screened using MSMS technology to include the following additional disorders: Batten disease types 1 and 2, Wolman disease, Niemann-Pick-C, MPS-IIIA, Cerebrotendinous Xanthomatosis, Wilson disease and Cystinosis.

We will present the results of a multiplex MSMS assay requiring a single 2-minute analysis time per dried blood-spot, thus opening up new possibilities for expanded newborn screening of conditions that are amenable to treatment or benefit from early clinical intervention.

### O29. Introduction and Summary of Pre-Conference Workshop

DerraikJoseAuckland, New Zealand

Abstract not available.

### O30. Evaluation of Laboratory Quality in Newborn Screening 

MeiJoanneZobelSherriWilliamsIrenePetritisKonstantinosBernsteinJohnCuthbertCarlaNewborn Screening Quality Assurance Program, Centers for Disease Control and Prevention, Atlanta, GA 30341, USA

Background: The Newborn Screening Quality Assurance Program (NSQAP) is an accredited proficiency testing (PT) provider (ISO/IEC 17043:2010). NSQAP provides comprehensive dried blood spot (DBS) PT materials used by laboratories to achieve certification by regulatory bodies and quality control materials (QC) for monitoring method performance.

Methods**:** NSQAP distributes DBS for fifteen PT and eleven QC programs. NSQAP collects PT data from participating laboratories and provides individual laboratory evaluations. NSQAP tracked the number of false positive (FP) and false negative errors (FN) over a five-year period, compared to the number of labs enrolled. Data collected from QC materials was sorted by method to allow comparisons within and between methods.

Results**:** Between 2014 and 2017, NSQAP experienced a growth in PT laboratories of 15% and an increase in PT errors of 21%. The five-year growth and error rates were 13 and 22%, respectively. Certain labs repeatedly made errors, despite enrollment in PT programs for years. Overall, more errors were reported for analytes tested by tandem mass spectrometry. For QC, linear regression analysis showed most methods routinely demonstrated acceptable performance when comparing analyte recovery with enrichment; five of 68 analytes showed lower recovery.

Conclusions**:** NSQAP currently identifies FN and FP errors on individual laboratory evaluations. As part of a quality management system, the laboratory should investigate sources of errors and adopt measures to minimize future risk. NSQAP PT evaluations are important tools to aid laboratories in demonstration of individual laboratory performance and peer comparisons and should be used for continuous quality improvement as part of good laboratory practice.

### O31. Evaluation of CAH Screening

FuJunfenDivision of Endocrinology, The Children’s Hospital Zhejiang University School of Medicine, Yan’an Road, Zhu Gan Avenue 57#, Hangzhou 310003, China

Congenital adrenocortical hyperplasia (CAH) is a group of autosomal recessive inheritance disorders, characterized by impaired cortisol synthesis. More than 90% of the CAH cases result from deficiency of the enzyme steroid 21-hydroxylase (21-OHD). The Endocrine Society updated a new version of clinical practice guideline in 2018 with combination of the latest published evidence and expert opinion. The new guideline proposes new recommendations on screening methods, treatment exploration and follow-up evaluation. With review of the International CAH Guidelines and the consensus on the CAH screening and management in China, we have screened over 2 million neonates since 2013, among which 103 CAH cases were diagnosed including 66 boys and 37 girls. More attention has been paid to the supplementation of corticosteroids and sodium and the hospitalization rate declined over the year. We highlight neonatal screening status in our hospital, together with treatment and monitoring of 21-OHD which mainly focus on linear growth of growing individuals with CAH and reproductive function of adult patients. And we emphasize that CAH, as an endocrine disease that covers the whole life cycle, needs to be treated jointly at all ages and in a multidisciplinary approach.

### O32. Cystic Fibrosis Screening—What Is a Missed Case?

HeatherNatashaByrnesCassJaksicMirjanaTateJanWebsterDianneNZ newborn metabolic screening program, LabPlus, Auckland City Hospital, Auckland 1148, New Zealand

Screening program sensitivity is a good measure of program reliability and is the proportion of cases detected by the program to cases detected + cases missed. However, the count of missed cases varies according to the definition used. CF cases can be missed at all steps of the screening and diagnostic pathway and mild cases present at any age.

Some areas of ambiguity are whether the screening program

- Includes short term follow-up as part of the screen, so that babies with positive screens but ‘normal’ sweat tests are included in the count of missed cases.
- Includes the presence of meconium ileus (MI) as the first test, hence a baby with MI and in-range IRT would proceed to DNA testing and if CF would be a detected case.
- Includes babies at increased prior risk (MI, family history) – if IRT or DNA not out of range are counted as a missed case,
- Aims to detect all disease, including mild and very late presenting cases which may or may not have been helped by early detection.

We presented a series of possible “missed cases” designed to explore opinion and practice amongst the international newborn screening community. A questionnaire was presented to attendees at the 2018 ISNS AGM and will also be presented to attendees of the 2019 APHL newborn screening meeting. Preliminary analysis revealed wide variation amongst ISNS attendees.

Performance data is highly dependent on the definition of a missed case. To enable meaningful comparison of screen performance, programs must clarify what steps in the screening pathway are included in the assessment, the screening algorithm and the screening target.

### O33. Newborn Pulse Oximetry Screening in China: An Integrated Public Health + Public Policy Approach

HuangGuoyingSaarinenAnnamarieChenJiaLiuFangZhuJunChildren’s Hospital of Fudan University, Pediatrics, Pediatric Cardiology, Shanghai, China

This session will provide program stakeholders and public health leaders with a case example of how newborn pulse oximetry screening for congenital heart disease has been studied, piloted, tested and implemented in China. A panel will share the latest information, strategies and tools to fully leverage the role of program leaders, clinical implementors and policy makers in supporting point of care screening as an integrated part of newborn screening. A panel of clinicians, public health experts and policy/advocacy leaders will walk attendees through the process of how China has addressed the challenges and opportunities of newborn screening for CHD and CCHD in a nation with vast geography, treatment capacity hurdles and among the highest annual births in the world. The lessons learned can inform emerging pilot projects and scalability in countries throughout Asia and internationally. The concept of newborn screening for congenital heart disease (CHD) developed slowly from small studies between 2002 and 2009 to larger population based studies in Europe and the U.S. from 2010 onward. The U.S. became the first nation to formally add Critical Congenital Heart Disease (CCHD) screening to the Recommended Uniform Screening Panel (RUSP) in 2011. Around this same time, China began researching the feasibility of newborn pulse oximetry leading to the largest published study of newborn CCHD screening in 2014. Continued study lead to a 2018 country-wide implementation process utilizing a "dual-index" method for screening. The presentation will focus on public health and clinical implementation methods utilized to effectively scale POC screening across diverse settings and jurisdictions with a focus on actions taken for following positive screens, reducing false positive and false negatives and helping to identify gaps in follow-up diagnostics, referral pathways and treatment infrastructure.

### O34. Philippine Multicenter Pulse Oximetry Screening for Critical Congenital Heart Disease (CCHD): A Preliminary Report

Jonas del RosarioJoseLiberty AlcausinMaria MelanieMariz DajoyagKevinaPadillaCarmencitaUniversity of the Philippines Manila, Newborn Screening Reference Center, National Institutes of Health, Ermita Manila, Philippines

Background: Congenital heart disease occurs in 9 in every 1000 live births and approximately one quarter (2–3 out of 1000) of these children will have critical congenital heart disease (CCHD). Despite the increasing use of prenatal diagnosis, a significant proportion of affected newborns are still not diagnosed before discharge after birth. Pulse oximetry screening (POS) is proposed as an effective, non-invasive, inexpensive tool allowing earlier diagnosis of CCHD. In the Philippines, there is no public health program requiring CCHD screening prior to hospital discharge.

Objective: This study aims to provide data on the utilization of POS as screening tool in detecting neonates at risk of having CCHD.

Method: All full-term, non-oxygen requiring, healthy newborn infants delivered in each of the participating hospitals whose parents consented to the procedure, are included in the study. POS based on the Minnesota protocol is performed on babies within 24 to 72 hours of life. A motion tolerant pulse oximeter (Masimo iSpO2 Pulse Oximeter) is used.

Results and Discussion: Six (6) institutions are currently enrolled in the study which is now at 20% of data collection. Among the 6, 527 newborns screened for CCHD, nine (9) failed the screening. Two (2) have persistent pulmonary hypertension (PPHN), four (4) have pulmonary conditions and one (1) with sepsis. Two (2) newborns with failed screening are cases of CCHD, both with transposition of the great arteries (TGA). Both underwent balloon atrial septostomy and are for arterial switch procedure. The results of the study will provide a basis for future policies on the inclusion of POS in the national comprehensive newborn screening policy in the Philippines.

### O35. Learning from the Past, Going Forward

HowellR. RodneyUniversity of Miami, Miller School of Medicine, Miami, FL, USA

More than 50 years ago, it was discovered that babies born with a rare genetic disorder due to an inability metabolize phenylalanine, which then led to an accumulation of this amino acid in the blood, suffered profound developmental delay (later named PKU). It was shown that babies could avoid this developmental delay if begun on a low phenylalanine diet soon after birth. Such treatment for this autosomal recessive disorder, however, required a highly reliable test for elevated phenylalanine concentrations suitable for testing every single newborn baby. Such a method, using dried blood spots collected from the baby was developed by Robert Guthrie. It was soon clear that babies with PKU, detected by this new test and treated with a low-phenylalanine diet, developed normally. This remarkable discovery led to the establishment of newborn screening, as the system was named, in many parts of the world, over the past 50 years. This most successful system to detect and treat PKU, led to the addition of tests for other conditions, one at a time, for a variety of other disorders, in a disorganized fashion around the world. Each area and country had a different panel. Tandem mass spectroscopy (MS/MS) was soon introduced into newborn screening, which readily permitted the measurement of a vast number of compounds from these same dried blood spots. This led to widespread addition of conditions to the newborn screening panels and enormous variation around the world.

In the United States in 2005, under a federal contract, the American College of Medical Genetics reviewed current screening panels and based on the available evidence, devised a Recommended Uniform Screening Panel or RUSP. Based on this work, over 30 conditions for well-established programs in highly developed countries is now recommended. During this same period, many highly specific drugs were being developed for rare, often fatal disorders. For optimum benefit, these treatments needed to begin in infancy and therefore require newborn screening. Some disorders require not only enzyme assays as the newborn screening tests but some also require DNA-based tests. These screening tests can be expensive and the highly-specific treatments, which include such as antisense-oligonucleotides, other small special molecules and gene therapy, are extremely expensive. Although widely discussed, first first-tier newborn screening using whole genome or whole exome sequencing is unlikely to occur soon due to difficulty in interpreting variants of unknown significance in such sequence data. As we move forward, the expansion of newborn screening must require not only the use of evidence based data about conditions and tests but also the evaluation of the economic costs of the tests and treatments and the readiness and availability of newborn screening laboratories. These factors will ensure that widespread variation throughout the world will continue to exist based on the many different but correct regional answers to these questions.

### O36. Pandora’s Jar in a Crystal Ball—Promises and Pitfalls of NGS in Newborn Screening—Proceeding with caution

SchielenPeterSinkeRichardNelenMarcelDolléMartijnDekkersEugenieWeversRonSpronsenFrancjan vanNational Institute for Public Health and the Environment, Reference Laboratory Neonatal Screening, Centre for Health Protection, Bilthoven, The Netherlands

Multiple next generation sequencing (NGS) techniques to expand and improve Newborn screening (NBS) are arising and DNA analysis can now be done using a single dried blood spot (DBS). NGS-first techniques allow identification of diseases lacking reliable biochemical footprints in DBS or antedate diagnosis of candidate NBS diseases, as recognized by the Dutch Health Council in 2015. Internationally, the case is made for genetic analysis in neonatal screening, given, for example, the ‘BabySeq project’ and first publications on exome analysis on small cohorts of newborn screening samples.

Reviews exploring the possibilities of NGS-in-NBS analysis and pilot studies (both targeted and whole-exome or -genome approached) presented possibilities and challenges of genomic analysis in NBS, while recent pilots in our own laboratory showed the feasibility of NGS analysis for many genes.

While analytically the possibilities are ever increasing at increasingly lower cost, to add meaning to the generated genetic data, making DNA analysis a useful parameter in the risk estimation for the diseases in newborn screening, is another step to take and a difficult one.

Questions that quickly arise are, for example, what metabolic diseases are particularly eligible for genomic testing in NBS, whether we can develop a rapid NGS based workflow (including analytical and bio-informatic pipeline) and whether we can and should incorporate this pipeline in the screening laboratory. Answering these questions is heavily dependent on the current and foreseen state of technology but also on the current and foreseen state of counseling on screening results. While that makes the challenge to plan implementation of NGS in NBS even more complex, here we want to present a realistic view in how NGS can help NBS in a responsible way in the next 5–10 years.

### O37. Really Inside the Crystal Ball

ChristodoulouJohnBrain and Mitochondrial Research Group, Murdoch Children’s Research Institute, Melbourne, Australia

There is little doubt that the development of newborn screening has been one of the great global public health successes of the last millennium. We have seen the scope of newborn screening evolve from metabolite analysis, to protein or enzyme quantitation and in more recent years, targeted molecular genetic testing. As the scope of newborn screening has advanced, so too have the available technologies. So, what might the multi-omic era have to offer?

Exploratory studies are currently under way evaluating the clinical utility of genomic sequencing in newborns, either as an alternative first-tier test for the genetic disorders for which newborn screening is currently performed or examining the potential utility of genomic testing beyond the newborn period. Whilst the technical aspects of genomic sequencing in newborns are steadily improving and bioinformatic analysis is becoming more sophisticated and “easier,” the current costs of genomic sequencing preclude it from being rolled out at a population scale, at least under the current newborn screening paradigm. Moreover, consumer engagement in these exploratory studies has not been as enthusiastic as had been hoped. of great importance, there are very significant potential ethical concerns associated with newborn genomic sequencing.

In this glimpse into the newborn screening crystal ball, these threads will be drawn together and a personal view on what the short, medium- and longer-term future might look like will be presented. It is this presenter’s view that whilst we are not yet ready for the introduction of genomic sequencing as a mass screening tool in newborns, it will inevitably become a reality. There is an opportunity for the newborn screening community to bring to bear its decades of collective experience and rigor to help build robust processes for the responsible incorporation of this and other emerging technologies into newborn testing.

## 3. Posters

A. Congenital Hypothyroidism

### P1. Analysis of Children with Congenital Hypothyroidism in Neonatal Disease Screening For 20 Years

ZouHuiJinan Maternity and Child Care Hospital, Neonatal Disease Screening Center, Jinan, China

Objective Analyze the treatment and prognosis of children with congenital hypothyroidism diagnosed by neonatal screening.

Methods Give oral medication to children with CH or high TSH(TSH > 10) diagnosed by screening at Jinan Newborn Screening Center. Children with CH can try to stop the drug after 2–3 years of standard treatment. Children with high TSH can try to stop taking drugs after half a year and retrospective analysis.

Results In 1996–2016,the total number of screenings was 1,266,485, of which 522 were diagnosed with CH and the incidence of CH was about ½,426,227 cases were discontinued after treatment and the drug withdrawal rate was about 43.5%,295 cases were still taking drugs, accounting for 1/4293 of the total number of screenings.305 cases of high TSH disease requiring drug treatment, after 2-3 years of treatment, 35 cases could not be stopped, accounting for 11.48% of high TSH.75 patients who were referred or lost to follow-up. There were 198 cases(87.22%)with normal thyroid tumors in the children who discontinued the drug and 103 cases (46.81%)in the children who did not discontinue the drug, the difference was statistically significant (*p* < 0.05).There were 827 children with CH and high TSH, including 12 cases combined with short stature and 4 cases combined with 21-trisomy.The incidence of CH and high TSH combined with short stature(1.45%) is lower than the normal population combined with short stature(3%).The incidence of 21-trisomy combined with CH (1/207) was significantly higher than that of normal population (1/2426).

Conclusion Children with CH diagnosed by neonatal screening have a higher rate of discontinuation after standard treatment; part of children with high TSH may be converted to permanent hypothyroidism; the condition of thyroid B ultrasound has important guiding significance for the prognosis of children; children with CH and high TSH have a good prognosis through early formal treatment; it should be noted whether 21-trisomy children have congenital hypothyroidism.

### P2. Factors Affecting Neonatal Hyperthyrotropenemia—A Prospective Study

SaitHaseenaGargRitikaJindalAnkurBKThelmaKapoorSeemaMAMC, Division of Genetics, Department of Pediatrics, New Delhi, India

Objective: To study maternal and neonatal factors associated with transient neonatal hyperthyrotropinemia (TNH) and its effect on the development assessed at 3 months of age.

Design: Case Control analytical study

Setting: Tertiary care hospital in North India

Participants: The case group (neonates with initial TSH values between 10–20 mIU/L with return to normal value of TSH <10mIU/L within 2 weeks of age) and control group (TSH ≤10mIU/L), comprising of seventy newborn mother dyads.

Methods: TSH estimation was done by fluroimmunoassay, fT3 and repeat TSH estimation by chemiluminescence. Maternal urine iodine concentration (UIC) was estimated by wet digestion method and anti TPO antibodies by ELISA. Developmental assessment was done at 3 months using Developmental Assessment Scale for Indian Infants (DASII).

Outcome: Factors responsible for TNH, development quotient and thyroid profile at 3 months of age.

Results: Out of 11,739 births, (86% screened), mean TSH values being 18.45 mIU/L and 3.03mIU/L in case and control group. TNH was more common in females [1.25:1] (p<0.001) and in those whose mothers had thyroid dysfunction(p<0.003). Maternal UIC reflected deficiency in 30% and 8% in the case and control group. Two of the neonates with TNH had low DQ score with high TSH and low fT4 on repeat thyroid profile when re-evaluated at 3 months of age.

Conclusion: Development assessment and a repeat thyroid profile should be done in all infants who show evidence of TNH, especially in infants born to mothers with hypothyroidism, low UIC or evidence of autoimmunity in order to initiate prompt therapy and reduction of disability.

**Keywords:** hypothyroidism; transient hypothyroidism; newborn screening

### P3. Cascade Screening of First Degree Relatives (FDRs) of Children with Congenital Hypothyroidism (CH): Report from a Tertiary Care Facility in North India

ChaudharyPujaSaitHaseenaDabbasAashimaPradhanGauravBKThelmaYadavSangeetaKapoorSeemaMaulana Azad Medical College, Division of Genetics, Department of Pediatrics, New Delhi, India

Objective: To estimate the proportion of structural or functional abnormalities of the thyroid gland in FDRs of children with CH.

Setting: Genetic & Endocrine clinic of a tertiary care facility in North India.

Methods: TSH estimation was initially done on dried blood spot using two site fluroimmunometric assay and values of >10 mIU/L were reconfirmed by analyzing the full thyroid profile by electro chemiluminescence assay. Volumetric assessment of thyroid gland was done by a single observer using 7–12 MHz ultrasound probe. Published normative data was used to ascertain hypoplasia.

Results: 211 FDRs of 74 index cases of CH were included. Thyroid dysgenesis (TD) was identified in 24 and dyshormonogenesis in 50 index cases. FDRs included 74 mothers, 72 fathers and 65 siblings. Consanguinity and inbreeding was present in 18.9% and 13.5% families respectively. Thirty four FDRs (16.11%) demonstrated abnormal USG findings. Colloid cyst in 5.2%, adenomatous nodule in 1.9%, diffuse thyroid disease in 7.8%, hyperplastic nodule 0.5%, colloid nodule with calcification in 0.5% and left hemigenesis with absent isthmus in 0.5%, thyroid hypoplasia in 18% of the FDRs was identified. Sixteen FDRs (7.6%) comprising 15 mothers and 1 sibling had hypothyroidism (abnormal venous TFT). Six mothers with deranged thyroid function (40%) also had anti TPO antibody positivity. None of the FDRs were symptomatic at presentation. Growth and development of all the siblings was normal.

Recommendations: Both structural and functional abnormalities are higher than expected by chance in FDRs of children with CH. Cascade screening of asymptomatic FDR’s if of great value. Further exploration of the role of genetic, epigenetic and autoimmune factor is desired for reaching a logical conclusion.

### P4. Closer Surveillance for both Iodine Deficiency and Excess Affecting Newborn Screening Results: The Changing Paradigm

KRDeepashreeSaitHaseena†YadavSangeetaJunejaMonicaGuptaSangeetaJindal AnkurKapoorSeemaMaulana Azad Medical College, Division of Genetics, Department of Pediatrics, New Delhi, India†Joint first authors.

Objective: To study the effect of iodine excess in pregnant mothers on the thyroid function, growth and neurodevelopment of the neonates when assessed at 3 months of age

Setting: Antenatal wards, Genetic and Endocrine OPD of a tertiary care center in North India

Methods: 400 newborn mother dyads with maternal urinary iodine >300 µg/L were prospectively followed up. Term neonates with birth weight > 2.5kg were included. Neonatal TSH was collected by heel prick at 24 hours of age or at discharge (whichever was later) and estimated by time resolved fluroimmunoassay. The maternal urinary iodine concentration was estimated by wet digestion method.

Results: The average maternal age was 25.1 years with 52% being multiparous & 72% delivered vaginally. All were exposed to povidone iodine containing antiseptic. Iodized salt consumption was present in 97.5% population. The mean neonatal TSH was 3.6±0.6 mIU/L and 12.3 % neonates had TSH value > 5mIU/L. No significant correlation was observed between TSH and gestational age, parity, birth weight and mode of delivery. No correlation was observed between maternal UIC and neonatal TSH. Mean TSH at 14 days and 3 months of age was 5 ± 3.7 mIU/L and 4.3 ± 2.4 mIU/L. The neurodevelopment of infants by DASII was normal for age.

Recommendation: Iodine excess causes only a transient rise in neonatal TSH. This has increased the recall rates with no adverse impact on the neurological development of these neonates. Use of povidone containing antiseptics should be seriously questioned.

### P5. Critical Cutoff for Newborns to Improve Reporting for Thyroid Stimulating Hormone

MajidHafsaHabibAyshaAhmedSibtainHumayunKhadijaSiddiquiImranKirmaniSalmanJafriLenaAga Khan University Hospital, Pathology, Karachi, Pakistan

Background: In order to strengthen our laboratory post analytical services, ensure patient safety and enhance quality of reporting, neonatal TSH results have been embedded in our laboratory’s critical informing list. This audit was performed to verify that whether the critical cut-off value of 20 mIU/L for neonatal TSH results is applicable in Pakistani population with a different biochemical make up compared to the Caucasians.

Methods: A retrospective cohort analysis of TSH results was conducted at the section of Chemical Pathology, Department of Pathology and Laboratory Medicine over two years period, from January 2015 to December 2016. Serum samples for TSH were analyzed at an automated analyzer ADVIA Centaur (Siemens Diagnostics, US) using chemiluminescence immunoassay technique. Abnormal TSH cutoff for critical result informing was defined after literature search and consensus of pediatric endocrinologist. Attending physicians or parent/guardians (in case of outside referrals) were informed if their patient’s TSH results were abnormal. In follow up the reported patients were contacted telephonically to record clinical details regarding congenital hypothyroidism (CH) on a predefined questionnaire.

Results: After literature search >20uIU/ml were taken as critical limits for TSH in consensus with pediatric endocrinologist. Total 27407 test were performed over two year’s period, 41% (*n* = 180) had a value of >20uIU/ml. Repeat TSH was performed in 26% (*n* = 46) and 7% (*n* = 7) of these had TSH levels >20uIU/ml. While free thyroxine (FT4) was tested in 18.3% (*n* = 33); and it was low in only 9% (*n* = 6) of these neonates. Based on repeat TSH and FT4 testing CH was diagnosed in 12 subjects only and lowest initial TSH noted in these subjects was 21.3uIU/ml.

Conclusion: We recommend a cut off value of >20uIU/ml TSH levels can be used as a critical for CH screening in Pakistani population and results must be timely informed to physicians to prevent delays in management and ensure better outcomes

### P6. Evaluation of the Performance of Genetic Screening Processor and Setting the Cut-off Values in Screening for Congenital Hypothyroidism in Newborn

WangLiwenNiMinZhaoDehuaLiuSunaThe Third Affiliated Hospital of Zhengzhou University, The Neonatal Screening Center of Henan Province, Zhengzhou, China

Objective: To evaluate the feasibility of genetic screening processor (GSP) applied in the screening for congenital hypothyroidism (CH) in newborns by detecting thyroxine stimulating hormone(TSH) in blood and to establish the cut-off value of screening TSH for CH in newborns in Henan province.

Methods: The precision and accuracy of GSP were calculated by detecting the dried-blood spots specimen of the Centers for Disease Control and Prevention. Detect the linear sample of 6 concentration gradient specimens to calculate the linearity of GSP. The neonatal heel blood samples were collected and the TSH in the dried-blood spots was detected using the Perkin Elmer GSP test kit. The TSH cut-off value was determined by the percentile method and the receiver operating characteristic (ROC)curve method.

Results: The within-run coefficient of variation of GSP screening system was 4.32 % ~ 5.87 %; The between-run variation was 4.45 %~9.08 %. All of them were less than 1/3 of the total error (TEa). The biases detected by the GSP system was -7.89%~0.12%, less than 1/2 TEa. The linear equation for observed TSH value by expected value was *y* = 0.908*x* − 0.915. The correlation coefficient(R2) was 0.998. percentile method of 99% confidence interval was 6.1 μU/ml. The critical value ROC curve method was 6.45 μU/mL.

Conclusions The quantitative performance of the GSP system can meet the laboratory requirements. It is a feasible method for neonatal CH screening. The cut-off value was set at 6.1 μU/ml.

### P7. Screening for Congenital Hypothyroidism in Qingdao over the Period 1997–2012: Incidence Trends and Clinical Types

ZhanLiqinNewborn screening center, Qingdao Women and Children Hospital Affiliated to Qingdao University, Qingdao, China

We aimed to investigate the incidence and clinical types of congenital hypothyroidism (CH) identified during neonatal screening in Qingdao, Shandong Province, China, during the period 1997–2012.

Design. Cross-sectional survey. Patients. Children with laboratory-confirmed CH at the Qingdao Neonatal Disease Screening Center who were seen between January 1, 1997 and December 31, 2012 were enrolled. Measurements. The clinical types of CH, including persistent congenital hypothyroidism (PCH), transient congenital hypothyroidism (TCH) and hyperthyrotropinemia (HT), were determined according to the patients’ condition following drug discontinuation. Incidence trends were determined and correlated to screening and imaging methods.

Results. The overall incidence rate of CH in Qingdao in 1997–2012 was 43.89 cases per 100,000. The incidence rate of PCH was 20.34/100,000; of TCH, 15.31/100,000; and of HT, 6.59/100,000. Trend analysis showed that CH and TCH were on an upward trend during this period (*p* < 0.01). The screening method used (enzyme-linked immunosorbent assay [ELISA] and time-resolved fluorescence immunoassay [TRFIA]) had a significant impact on the incidences of CH and TCH (*p* < 0.05). Abnormal thyroid development was observed in 80.39%, 44.31% and 9.85% of PCH, CH and TCH patients, respectively. Serum free thyroxine (FT4) level was significantly higher in TCH patients than in PCH patients (*p* < 0.05).

Conclusions. The incidences of CH and TCH have shown an increasing trend in the past decades. TRFIA is more sensitive than ELISA in screening for CH and TCH. Thyroid imaging findings and serum FT4 level are useful in determining the clinical type of CH.

### P8. Analysis and Assessment of Cut-off Value for Congenital Hypothyroidism on Newborn screening in Zhejiang Province

XuYanhuaYangRulaiZhengJingChenDingwanThe Children’s Hospital Zhejiang University School of Medicine, Department of Genetics and Metabolism, Hangzhou, China

Objective: To analysis the distribution of thyroid-stimulating hormone (TSH) in newborn screening and evaluate the cut-off system of congenital hypothyroidism (CH) in Zhejiang Province.

Methods: According to the dates of CH newborn screening from the Lab of Neonatal Screening Center of Zhejiang Province during 2017, the analysis and assessment of cut-off value for CH were investigated by using percentile method and ROC curve.

Results: Totally 614005 blood sample were screened during 2017, Among which 21,805 newborns were recalled.560 neonates were diagnosed with CH, 2719 with high TSH hyperlipidemia. The morbidity rate of CH was 1:1096. The cut-off value of TSH in newborn screening was skewed, P50 was 2.43 mIU/L and P99 was 10.00 mIU/L. Combined with ROC curve, the cut-off value of Zhejiang province was 9.005 mIU/L. Considering various factors, the optimal cut-off value of CH was 9.00mIU/L, the sensitivity, specificity and misdiagnosis rate were 99.51%, 98.81% and 0.15%, respectively.

Conclusions: It is important to establish the appropriate cut-off value according to the actual situation of the province. Appropriate screening cut-off value can provide scientific basis for newborn screening and improve screening effectiveness.

**Keywords:** cut-off value; congenital hypothyroidism; thyroid-stimulating hormone; newborn screening; Zhejiang

### P9. Bridging the Gap in Pediatric Reference Intervals (RIs)—Data mining of Laboratory Information System for Establishment of RIsE of Neonatal Thyroid Stimulating Hormone at a Tertiary Care Hospital in Pakistan 

AhmedSibtainJafriLenaHabib KhanAyshaMajidHafsaGhaniFarooqSiddiquiImranThe Aga Khan University, Pathology & Laboratory Medicine, Karachi, Pakistan

Background: Neonatal screening for thyroid stimulating hormone (nTSH) is conducted in our hospital for the timely diagnosis and treatment of congenital hypothyroidism. However, reference intervals (RI) of nTSH are known to be method and population dependent as well as age specific. So, the aim of this study was to determine the reference interval of nTSH based on laboratory data.

Material and methods: A cross-sectional analysis of results of serum nTSH of neonates (≤1 month of age) acquired from October 2015 to March 2016. Analyses of TSH serum samples was performed on an automated immunoassay system (ADVIA^®^ Centaur™, Siemens Diagnostics, NY, US) using a direct chemiluminescence detection system.

The CLSI recommended method was used for the determination of upper and lower end points covering 95% of the reference values of each analyte with respective 90% Confidence intervals (CI). Subjects were sub-grouped according to age, ≤5 days of life and 6 days to 1 month. Standard statistical methods, including the Reeds test and the Kolmogorov–Smirnov test were used for data analysis using the MedCalc 16.2.1 software package (MedCalc, Mariakerke, Belgium).

Results: Total 6400 NTSH tests were performed over a period of 6 months, amongst them 88% (*n* = 5610) were aged ≤5 days of life. There was a near equal gender distribution for both age group.

The RI for nTSH using the CLSI recommended method for ≤5 day’s age group was calculated to be 0.62 uIU/mL to 14.97 uIU/mL with CI of 0.58–0.67 and 14.5–15.63 uIU/mL respectively. For the 6 days to 1 month age group RI was 0.462 uIU/mL to 9.102 uIU/mL with CI of 0.35–0.62 and 8.5–10.6 uIU/mL correspondingly.

Conclusion: Our results show that age specific RIs for nTSH in neonates differ from population to population. We recommend reporting neonatal TSH values in neonates using age and population specific reference intervals.

### P10. Discussion on Early Predictors of Different Classification of Congenital Hypothyroidism

YanYaqiongShanxi Maternal and Child Health Hospital, Neonatal Disease Screening Center, Taiyuan, China

Objective To explore predictive factors that can differentiate early children with different classification of congenital hypothyroidism (CH).

Methods The subjects who were diagnosed in Shanxi Province neonatal disease screening center from October 2009 to October 2014 and treated with L-T4 for 2–3 years and who accepted the follow-up treatment for more than 1 year after drug withdrawal are enrolled. According to the follow-up results, permanent hypothyroidism (PCH) and transient hypothyroidism (TCH) were distinguished. The clinical basic conditions of the two groups and the L-T4 dose during follow-up period were compared to find early predictors.

Results The differences in initial screening TSH level, initial treatment timing, time required for TSH to return to normal, thyroid situation composition and L-T4 dose during follow-up period were statistically significant for children with PCH and TCH. There were no significant differences in gender composition, gestational weeks, birth weight, initial treatment months composition and time required for FT4 to return to normal. In addition, during the follow-up period, the dose of PCH increased with age, while the dose of TCH decreased with age. The initial screening TSH levels for children and L-T4 dose at 7 months after diagnosis can be used for early differentiation between children with PCH and TCH.

Conclusions (1) The initial TSH screening levels and the dose of L-T4 during the follow-up period have predictive significance for early differentiation of PCH and TCH; (2) Combined with etiology, initial screening TSH level and TSH and FT4 levels at diagnosis, initial differential dose administration for children is of great significance for the early recovery of thyroid function in children.(3) For some children requiring low dose L-T4, advancing the time of drug withdrawal and of re-evaluation can be taken into consideration.

**Keywords:** congenital hypothyroidism; permanent hypothyroidism; transient hypothyroidism; initial screening TSH level; L-T4 dose

### P11. Key Performance Indicators of the Screening for Congenital Hypothyroidism Sri Lanka in Terms of Coverage, Effectiveness of Detection and Managing Cases

HettiarachchiManjulaAmarasenaSujeewaUniversity of Ruhuna, Nuclear Medicine, Galle, Sri Lanka

Introduction: Program evaluation using key performance indicators (KPI) is an important organizational practice in all public health program. We perform a retrospective evaluation of babies diagnosed with congenital hypothyroidism at our screening program (www.nsisd.ruh.ac.lk) in order to analyze clinical reflections of the program.

Methods: The database of the program was reviewed to analyze KPIs in 2017 and 2018. The coverage based on screened infants out of total live births, age at screening, at confirmation and at the time of commencing treatment were analyzed and the positive predictive value calculated on total positives (on true and false positives).

Results: The program coverage has improved to 92% by the end of 2018 with positive predictive value of 54%. There were 159 primary CH confirmed neonates out of 297 screening positives. The incidence rate of primary CH was 1:1910 and the false positive rate was below 0.05%. Analyses of the sample collection indicated that 6% (*n* = 18,536) of babies’ blood spots were collected <2 hrs of delivery; 41% (*n* = 24,894) in next 12 hrs; 21% (*n* = 64,774) on day 2; followed by 32% (*n* = 95,654). The age at serum sampling (for the confirmatory testing) was 6 to 31 days with an average of 17 (±7) days. Serum confirmation was made before 10 days of age among 18% (*n* = 29) and within 11–21 days of age for 52% (*n* = 83) of neonates. The mean age for thyroxine replacement therapy started by 21±9 days.

Conclusions: There is a need to improve the laboratory and clinical services to ensure that results are available and reviewed within the stipulated time frame. Liaison with the state public health department is important to promote community awareness and trace cases that required retesting. It is important to undertake periodic audits of the system to identify any deficiency and improve on the overall quality of the newborn screening program.

### P12. Analysis of Positive Recall Results of Screening for Congenital Hypothyroidism in Newborns in Guangxi from 2014 to 2018

LiWeiFanXinLinCaijuanXuYuqiHuangXiaotaoGuangxi Zhuang Autonomous Region Maternal and Child Health Hospital, The Center Laboratory of Genetics and Metabolism, Guangxi Neonatal Disease Scree, Nanning, China

Objective: To analyze the positive recall of screening for congenital hypothyroidism (CH) in newborns in Guangxi from 2014 to 2018, in order to provide a more scientific basis for further improving the recall rate.

Methods: Recalling suspiciously positive children with CH screening in Guangxi,10964 cases were tested for thyrotropin (TSH) concentration in blood samples by time-resolved fluorescence, and re-take blood or collect venous blood according to laboratory; If the cut-off value TSH≥8 IU/L, we detect TSH, FT3, FT4 to confirm.

Results: 945,713 cases were screened, of which 11,438 cases were suspicious and 10,964 cases (95.90%) were recalled. It was found that 5909 cases were recalled when the initial TSH results were 8≤TSH<10uIU/ml and of which 321 cases (5.43%)were diagnosed with CH(including Hyperthyroidism);When 10 ≤ TSH < 15 uIU/ml, 3599 cases were recalled and 313 cases(8.70%) of CH(including Hyperthyroidism) were diagnosed; If 15 ≤ TSH < 25 mIU/L,617 cases were recalled and 186 cases(30.15%) were diagnosed CH (including Hyperthyroidism).

Conclusion: When the initial screening result 8 ≤ TSH < 15 mIU/L, the false positive rate is >90% and accounts for 86.72% of the total recall, It is suggested to recall the re-collection of blood tablets for secondary review and if the review result was TSH≥8uIU/ml, Then, venous blood was used to detect the concentration of TSH, FT3 and FT4;When the initial screening TSH ≥ 15 uIU/ml, It is suggested to directly recall venous blood for TSH, FT3 and FT4 concentration detection, which can not only greatly shorten the diagnosis and treatment time, but also improve the screening efficiency and reduce birth defects.

**Keywords:** CH; TSH; Positive recall results

### P13. The Role of Genetic Factors in the Pathogenesis of Congenital Hypothyroidism in Guangxi China

FanXinChenShaokeMaternal and Child Health Hospital of Guangxi, Laboratory of Genetics and Metabolism, Nanning, China

Gene variants have been reported to be associated with only part of CH and the relationship between CH genotype and clinical phenotype has not yet been established. the purpose of this study was to analyze the mutation spectrum and prevalence of 12 known causative genes (*TSHR*, *PAX8*, *NKX2.1*, *NKX2.5*, *FOXE1*, *DUOX2*, *TG*, *TPO*, *GLIS3*, *NIS*, *SLC26A4* and *DEHAL1*) in 382 CH patient in Guangxi.

Methods: Peripheral venous blood samples were collected from the patients. Genomic DNA was extracted from peripheral blood leukocytes. All exons and their exon-intron boundary sequences of the 12 known CH associated genes in 382 CH patients were screened by next-generation sequencing (NGS).

Results: NGS analysis of 12 known CH associated genes revealed that 191 patients (191/382, 50.0%) were detected to have at least one suspected variant. The mutation rate of *DUOX2* gene was the highest, followed by *TG*, *SLC26A4*, *PAX8*, *TSHR*, *TPO*, *NIS* and *GLIS3.NKX2.1*, *NKX2.5*, *FOXE1* and *DEHAL1* genes did not detect any suspected mutation. PA8 gene heterozygous mutations are associated with Permanent CH(PCH), TSHR heterozygous mutations are associated with TSH elevation, TSHR with DUOX2/TG heterozygous mutations are associated with CH, most single or multiple DUOX2 mutations are associated with Transient CH (TCH) or TSH elevation and multiple DUOX2 mutation sites are associated with PCH.

Conclusion: Our study expands the mutation spectrum of CH genes, provides an overview of role of genetic factors in the pathogenesis of congenital hypothyroidism in Guangxi. Variable mutation frequencies of CH associated genes across this cohort. Currently we do not know if heterozygous variants are contributing the occurrence of CH or digenic mode also contribute to CH. More sequence data from large population will be needed to understand these questions, also will help to see how big fraction of CH have genetic underpinning rather than environmental factors.

### P14. Mutation Spectrum in TPO Gene of Bangladeshi Patients with Thyroid Dyshormonogenesis

Mizanul HasanMohammadBegumMst. NoorjahanKawsar KonikaTasniaSultanaSadiaSaleheen QadriSyedQadriFirdausiAkhteruzzamanSharifMannoorKaiissarNational Institute of Nuclear Medicine and Allied Sciences, Thyroid, Dhaka, Bangladesh

Although thyroid dyshormonogenesis (TDH) accounts for 10–20% of congenital hypothyroidism (CH), the molecular etiology of TDH is unknown in Bangladesh. Thyroid peroxidase (TPO) is most frequently associated with TDH and the present study investigated the spectrum of TPO mutations in Bangladeshi patients and analyzed the effects of mutations on TPO protein structure. Sequencing-based analysis of TPO gene revealed four mutations in 36 diagnosed patients with TDH including three nonsynonymous mutations, namely, p.Ala373Ser, p.Ser398Thr and p.Thr725Pro and one synonymous mutation p.Pro715Pro.Homology modelling-based analysis of predicted structures of MPO-like domain (TPO142-738) and the full-length TPO protein (TPO1-933) revealed differences between mutant and wild type structures. Molecular docking studies were performed between predicted structures and heme. TPO1-933 predicted structure showed more reliable results in terms of interactions with the heme prosthetic group. p.Ala373Ser and p.Thr725Pro mutations were more damaging than p.Ser398Thr. However, when the interactions between the crucial residues including residues His239, Arg396, Glu399 and His494 of TPO protein and heme were taken into consideration using both TPO1-933 and TPO142-738 predicted structures, it appeared that p.Ala373Ser and p.Thr725Pro could affect the interactions more severely than the p.Ser398Thr.Validation of the molecular docking results was performed by computer simulation in terms of quantum mechanics/molecular mechanics (QM/MM) and molecular dynamics (MD) simulation.

In conclusion, the substitutions mutations, namely, p.Ala373Ser, p.Ser398Thr and p.Thr725Pro, had been involved in Bangladeshi patients with TDH and molecular docking-based study revealed that these mutations had damaging effect on the TPO protein activity.

### P15. Clinical Outcome of Regulation of TSH And FT4 in Children with Congenital Hypothyroidism Seen in the Newborn Screening Continuity Clinic in the Southern Philippines Medical Center

NiñoraKrystle MarieAbarquezConchitaSouthern Philippines Medical Center, Department of Pediatrics, Davao City, Philippines

Background: Babies who are confirmed to have congenital hypothyroidism (CH) need long-term follow-up care ideally by Pediatric Endocrinologists. In the Philippines, there is limited number of endocrine specialists. In October 2014, the Department of Health ordered for an establishment of continuity care clinics in each region of the country to facilitate continuity of care of confirmed patients in its area of coverage. Currently, continuity of care of confirmed CH babies are handled by the NBS continuity clinics in the country.

Objective: To determine FT4 and TSH levels of patients given high dose thyroid hormones enrolled in NBSCC during the given time period. To describe the profile of the patients using the standard monitoring protocol for confirmed cases. To determine the clinical outcome over time of high dose thyroid hormone in patients starting within the two weeks of life or upon diagnosis and enrolled in NBSCC until the first year of life. To correlate the effect of Levothyroxine to the clinical outcome over time

Methods A descriptive study was employed and data was obtained through retrospective chart review. The study of the record of patients who were given a high dose of levothyroxine and had been followed-up and evaluated using the checklist from January 2014 to December 2018 was conducted at the NBS Continuity Clinic of Southern Philippines Medical Center.

Results: The study is ongoing and is expected to be completed by May 2019. Final results will be presented during the conference.

### P16. Detecting Missed Cases of Congenital Hypothyroidism During Repeat Newborn Screening of Preterm Babies

AbarquezConchitaSouthern Philippines Medical Center, Newborn Screening Center Mindanao, Davao City, Philippines

Background: Newborn screening for preterm babies requires serial testing because this group of neonates is at risk for false positive or false-negative screens. As a national policy, repeat NBS collection for preterm babies, regardless of status during initial collection, is recommended on the 28th day of life.

Objective: The study aims to determine the repeatability of TSH screening for premature infants, to determine the number of false-positive and false-negative CH cases in the newborn screening tests of preterm newborns and to describe the phenotypic spectrum of CH missed cases.

Methods: A seven-year review of data was retrieved from the Neometrics database. The data include number of babies with initial NBS sample, number of preterm babies with initial and repeat NBS samples, TSH values in both initial and repeat samples. This repeatability of TSH screening results was measured and compared using the paired t-tests.

Results: Results showed that out of the 2,000,796 babies screened from 2012 to 2018 in NSC Mindanao, 51,675 (2.58%) were premature. of this 51,675 preterm babies with initial newborn screening test, only 7,511 (14.54%) babies submitted a repeat dried blood spot for NBS at >28 days of life. Initially screened abnormal values for TSH showed no significant difference with repeat card. The mean difference of TSH values between initial and repeat samples was only 5.31µU/mL which denotes consistency of NBS screening for the abnormal results. Normal initial values of TSH were found to be significantly higher by 0.45 µU/mL, as compared to the results of repeat card. Twenty-four (24) cases of CH were missed during the initial newborn screening test.

Conclusion: Congenital hypothyroidism can be missed in the initial newborn screening test of a preterm newborn. To prevent missed diagnosis, there is a need to perform serial newborn screening tests for preterm infants.

### P17. DUOX2 Hotspots Variants and Outcomes of Patients with Congenital Hypothyroidism Suspected Thyroid Dyshormonogenesis

HuangYonglanTanMinyiJiangXiangTangChengfangFengYiLiuSichiLiPeiLiuJilianLiuLiGuangzhou Women and Children’s Medical Center, Guangzhou Newborn Screening Center, Guangzhou, China

Objective To investigate prospectively molecular and clinical characteristics of infants with congenital hypothyroidism (CH) caused by DUOX2 mutations in Guangzhou.

Methods A population-based cohort of 83 patients with CH was recruited based on newborn screening among 108899 newborns born in Guangzhou from Apri. to Sept. in 2015. Genetic analysis of DUOX2 hotspots (including 11 exons) by PCR-direct sequencing was performed in 52 patients with suspected thyroid dyshormonogenesis (SDH) according to thyroid ultrasound at diagnosis. All the patients were follow-up 3 years. The data of this cohort study were compared with those of 96 patients with SDH in 2011–2012.

Results (1) The incidence of CH in 2015 was 1:1312 (83/108899). 73.5% (61/83) of CH patients was classified as SDH. Compared with those founded in 2011–2012, the incidence of CH was increased significantly (1:1312 vs 1:2779, *p* < 0.001), while the frequency of SDH was not different significantly (73.5% VS 76.6%, *p* = 0.633). (2) 27 (51.9%) SDH patients were detected DUOX2 hotspots variants, including 6 cases with biallelic variants and 21 cases with monoallelic variants. The p.K530X was the most common mutation accounting for 50% (17/33) detected allelic and involving in 16(30.8%)SDH cases. Novel p.S1091F variant was probably damaging variant predicted by SIFT and PolyPhen-2. Both of detected rate and spectrum of DUOX2 variant were not significantly different compared with those in 2011-2012. (3) There were no significant differences in phenotypes between with-DUOX2 and non-DUOX2 variants cases. 24 (88.9%) patients with DUOX2 mutation were transient CH.

Conclusion This study confirmed that the incidence of CH was increased in last few years in Guangzhou. Most of them were SDH. Half of SDH were detected DUOX2 hotspots variants. The p.K530X was the most common mutation in this cohort population.

B. Congenital Adrenal Hyperplasia

### P18. Growth Analysis in 40 Japanese Patients with 21OHD—Influence of Glucocorticoid Dosage, Genotype and Phenotype on Growth Outcome

OkadaSatoshiKagawaReikoSakataSonokoHaraKeiichiTajimaGoHiroshima University Graduate School of Biomedical and Health Sciences, Department of Pediatrics, Hiroshima, Japan

Background: An Endocrine Society Clinical Practice Guideline of steroid 21-hydroxylase deficiency (21OHD) recommend the dose of hydrocortisone (HC) should be less than 20 mg/m2/day during the first year and less than 15 mg/m2/day during childhood. We evaluate the influence of HC and 5α-fludrocorticosterone (F) dosage and genotype on growth pattern and outcome.

Methods: We analyzed 40 patients with 21OHD. Final height (FH)/predicted final height (pFH) and loss of height potential related to target height (TH) were calculated. The patients were divided into 4 groups depending on the dose of HC and evaluated height outcome. (Group1: HC >20 mg/m^2^/day in first year, >15 mg/m^2^/day in 5-10years old, Group2: HC < 20 mg/m^2^/day in first year, > 15 mg/m^2^/day in 5–10 years old, Group3: HC >20 mg/m^2^/day in first year, < 15 mg/m^2^/day in 5–10 years old, Group4: HC< 20 mg/m^2^/day in first year, <15 mg/m^2^/day in 5-10years old) Mean FH SD score (SDS) and pFH SDS were analyzed in accordance to dosage of HC, F and genotype.

Results: Mean (FH SDS, pFH SDS) was −1.1 ± 0.83 SD, with 9% of all patients reached to FH exhibiting < −2SD. Mean (FH-TH) was −0.72 ± 0.92 SD. Goup2 exhibited trend of higher FH SDS, pFH SDS and smaller height losses. In growing patients, group1 were administered more HC and F and exhibited smaller body height during early childhood. On the other hand, genotype containing I172N alleles were administered less HC and F than other genotype but they tended to accelerate bone age.

Conclusions: HC substitution in 21OHD patients should be kept at the lowest efficient level, if possible <20mg/m2/day during the first year but bone age progression under 10 years old must be prevented with the optimization of HC dose. Genotype played only a minor role in growth outcome but a supportive information about growth pattern.

### P19. Determination of a steroid profile in dried blood spots by liquid chromatography-tandem mass spectrometry: Application to newborn screening for congenital adrenal hyperplasia

ZhanXiaYeJunQiuWenjuanHanLianshuZhangHuiwenLiangLiliGuXuefanShanghai Institute for Pediatric Research, Xinhua Hospital, Shanghai Jiao Tong University School of Medicine, Department of Pediatric Endocrinology and Genetics, Shanghai, China

Background: Conventional screening for congenital adrenal hyperplasia (CAH) using immunoassays generates a large number of false-positive results, especially in preterm neonates. The present study aims to improve the sensitivity of the CAH neonatal screening by including second-tier steroid profiling on a dried blood spots (DBS) using liquid chromatography tandem mass spectrometry (LC-MS/MS).

Methods: We developed and validated a LC-MS/MS method for the simultaneous determination of six steroids in DBS, including cortisol, 17-hydroxyprogesterone, 11-deoxycortisol, 21-deoxycortisol, androstenedione and testosterone. We analyzed 5400 full-term and 740 preterm or low-weight newborns samples, as well as 448 cards with a positive screening by the time-resolved fluorescence method (DELFIA).

Results: Values of intra- and inter-day precision coefficients of variance and recovery were 3.3–14.4% and 95.7–117.8%, respectively. The lowest limit of quantification for six steroids was 0.125 ng/mL to 1 ng/mL. The cut-off values of 17-OHP and (17-OHP+androstenedione)/cortisol ratio in full-term neonates were 2.5 nM and 0.73, respectively; while the cut-off values in preterm or low birth weight neonates were 5.0 nM and 1.16, respectively; According to the 17-OHP concentration and (17-OHP+androstenedione)/cortisol ratio of the 448 positive screening samples, total of 80 newborns should be recalled. The false-positive rate was reduced by 82.1%. Ten children were diagnosed with classic CAH due to 21-hydroxylase deficiency. The positive predictive value of DELFIA method was 2.2% and the positive predictive value of secondary screening with LC-MS/MS method was increased to 12.5%.

Conclusions: The second-tier steroid profiling by LC-MS/MS reduced the false- positive rate and increased the positive predictive value of CAH screening. The reduction in false positives significantly reduces anxiety for newborns and their families.

### P20. Mutation Spectrum of CYP21A2 Gene in Congenital Adrenal Hyperplasia Patients in Guangxi China

LuoJinsiLiMengtingYiShengFanXinGuangxi Maternal and Child Health Hospital, Department of Genetic and Metabolic Central Laboratory, Nanning, China

This study was performed to analyze the mutational spectrum of the CYP21A2 gene in Congenital adrenal hyperplasia (CAH) patients in Guangxi China, to identify mutational hot spots and to determine the correlation between the genotypes and phenotypes of CAH. The CAH patients were diagnosed by using tandem mass spectrometry (MS/MS) and Molecular diagnostic methods. 101 CAH families in Guangxi China was enrolled to analyze CYP21A2 gene variants using Sanger sequencing, Multiplex ligation-dependent probe amplification (MLPA). Statistical analysis was used to the correlations between CYP21A2 gene variants and clinical phenotypes of CAH patients. A total of 168 variants were detected in the 202 alleles of 101 suspicious patients with CAH (detection rate: 83.16%), with the three most frequent variants being c.293-13A>G (37.50%), c.518T>A (p.I173N) (15.48%) and c.1069C>T (p.R357W) (9.52%). Five different large deletion variants were identified by the MLPA method, including the large deletion exon1-3 with high frequency. Interesting, in these patients, there a patient who was diagnosed as Congenital adrenal hyperplasia with salt-wasting (SW) and simple virializing (SV) and high 17ohp screened by MS/MS many times in 2014–2019 but the result of the Sanger sequencing and MLPA of *CYP21A2* gene shows that the patient just c.293-13A>G heterozygous mutation carrier inherited from his father. The result indicate further studies are necessary to elucidate molecular mechanisms of CYP21A2 variants or other genetic factors responsible for CAH. In conclusion, the mutational spectrum underlying CAH in Guangxi China was established for the first time. Correlation analysis between variants frequencies in compound heterozygous patients and phenotype severity is helpful for phenotypic prediction.

### P21. Establish the Cut-off Value of Neonatal Screening for Congenital Adrenal Hyperplasia of Premature Newborns in Sichuan

HuQiSichuan Maternal and Child Health Hospital, Neonatal Screening Department, Chengdu, China

Objective Establish appropriate 17-hydroxyprogesterone (17-OHP) cutoff value in congenital adrenal hyperplasia (CAH) screening for Sichuan.

Methods Fluorescence enzyme immunoassay was applied to detect 17-OHP concentration in newborn dried blood spots on filter paper. A1l subjects were grouped according to different birth gestational weeks and used the percentile method to determine the cut-off value.

Result The distribution of 17-OHP levels was skew. The difference of 17-OHP concentration of the early infants and the moderate infants and later infants and the term infants had statistical significance (*p* < 0.05).percentiles of 99% 17-OHP concentration of l27 early infants was 120 nmol/L, percentiles of 99% 17-OHP concentration1 of 1402 moderate infants was 50nmol/L, percentiles of 99% 17-OHP concentration1 of 8772 later infants was 27 nmol/L, percentiles of 99% 17-OHP concentration1 of 82,568 term infants was 23 nmol/L. Suggestions are made for implement of 120 nmol/L as 17-OHP concentration cut-off value in early infant and 50 nmol/L in moderate infant.

Conclusion the cut-off value of neonatal screening for congenital adrenal hyperplasia of premature newborns should be established by different birth gestational weeks, in order to reduce false positive rate and needed recall rate.

### P22. Analysis of Newborn Screening for Congenital Adrenal Hyperplasia on Genetic Screening Processor (GSPTM)in Zhejiang Province, China

FangKexinWuDingwenYangJianbinYangRulaiThe Children’s Hospital Zhejiang University School of Medicine, Genetics and Metabolism, Hangzhou, China

Background: Congenital adrenal hyperplasia (CAH) is an autosomal recessive disorder with a worldwide incidence of 1:10,000 to 1:40,000 depending on the population. AutoDELFIA (AD) system previously applied to detect bloodspot 17-hydroxyprogesterone (17-OHP) concentration in newborn screening program for CAH. However, cross-reactivity with steroid sulfates and EDTA contamination of bloodspots would incorrectly elevate the false positive cases. In order to overcome the drawbacks, a new automated genetic screening processor (GSPTM) was introduced into newborn screening program. It was supposed to overcome most of the drawbacks of AD due to the improvements in chemistry and handling process.

Methods: We summarized the dates of CAH newborn screening from Neonatal Screening Center of Zhejiang province over the past year and a half. The 17-OHP was assayed by GSP. The GSP 17-OHP assay is a solid phase, time-resolved fluoroimmunoassay based on the competitive reaction. Compare the GSP results with previous AD results.

Results: In the study of 813,411 newborns screened from July 2017 to December 2018 the mean 17-OHP was 4.19 nmol/L (0.1–916 nmol/L) and the 99.5th centile 17-OHP was 11.2 and 39.2 nmol/L in full time and premature infants, respectively. 3454 infants were recalled for redetection. 25 cases were confirmed of CAH (range 18.2–916 nmol/L), give an incidence of 1:32,536, The incidence is higher in male infants than in female infants. Also, the incidence is higher in overweight infants (≥4000 g) than in other weight infants. An inverse association between 17-OHP and Gestational age was observed. The GSP can significantly reduce the false positive rate of CAH when compared with AD in premature infants. Moreover, 30 incomplete elution cases were identified by GSP.

Conclusions: GSP is suitable for routine newborn screening for CAH and it is a more specific and convenient system when compared with AD.

### P23. The Second-Tier Newborns Screening for Congenital Adrenal Hyperplasia by Liquid Chromatography Tandem Mass Spectrometry

GengGuoxingFanXinLinCaijuanLuoJingsiHuangXiaotaoChenShaokeMaternal and Child Health Hospital of Guangxi, Laboratory of Genetics and Metabolism, Nanning, China

Background Conventional newborn screening for congenital adrenal hyperplasia (CAH) is commonly accomplished by measurement of 17-α-hydroxyprogestrone (17-OHP) using enzyme immunoassay (EIA). EIA contributes a significant number of false positives. especially in preterm neonates, Therefore, we developed a second-tier steroid profiling method that uses liquid chromatography–tandem mass spectrometry (LC-MS/MS) to measure seven steroids in dried blood spots.

Methods To increase the specificity of the screening process, we developed a second-tier test for CAH use LC-MS/MS method to determine17-hydroxyprogesterone, cortisol, androstenedione, 11-deoxycortisol, Progesterone, dihydrotestosterone, aldosterone. Dried blood spots (DBS) were extracted with methanol containing the deuterium labeled internal standards of d8-17-OHP, d7-androstenedione, d4-cortisol, d3-dihydrotestosterone and d7-aldosterone in ultrasonic extractor. Analysis was done in positive ionization mode (ESI±) and recorded in multiple reaction monitoring mode (MRM).

Results Under the optimized condition, the results show that the linearity between the concentration of seven steroids with coefficients of regression >0.99. The mean recoveries were between 72.6% to 118.2%, the relative standard deviation is between 1.6 and 14.4%. Retrospective analysis of 190 samples include 24 samples with clinically confirmed 21 hydroxylase deficiency, as well as analysis of 110 premature infants samples include 26 cases is positives samples in our routine 17-OHP immunoassay. 24 samples with clinically confirmed were verified identical with the genetic results, 26 cases with positives samples is normal, no false-positives were found. Meanwhile we calculated the ratio between the seven steroids.

Conclusion This method shows a good precision and accuracy, which can be applied to screening of CAH, as a second-tier test can be used to reduce false-positive and improve the newborn screening program.

**Keywords:** newborn screening; congenital adrenal hyperplasia,17-hydroxyprogesterone; genetic screening processor

### P24. Newborn Screening for Congenital Adrenal Hyperplasia in Beijing

YangNanZhaoJinqiYangHaiheKongYuanyuanBeijing Obstetrics and Gynecology Hospital, Capital Medical University, Newborn Screening Center, Beijing, China

Objective: Investigating the incidence and the associated clinical characteristics of neonatal congenital adrenal hyperplasia (CAH) in Beijing.

Methods: Live birth newborns (*n* = 91,148) were screened for CAH in Beijing from July 2014 to February 2019. Blood sample was taken by heel prick between 72 hours to 7 days after birth. Blood samples were analyzed using a 1235 Auto DELFIA automatic immunoassay system. Neonates with a level >30 nmol/L of 17-OHP were called for a retest. CAH was diagnosed based on further laboratory findings combined with clinical signs, such as feeding difficulties, skin pigmentation and atypical genitalia. The lab tests included Na, K, 17-OHP, ACTH, T, F, adrenal ultrasound or CT scans, gonad ultrasound and genetic sequencing (combined analysis of PCR amplification and MLPA technology).

Results: of the 91148 neonates screened, 560 cases were deemed positive. Nine patients (six boys and three girls) were diagnosed with CAH. Seven cases of classic salt-wasting and two cases of simple virializing 21-OHD were identified. The incidence of CAH in Beijing was calculated to be 1:10,127. The follow up of their developments was observed to be almost normal with respect to height and weight gain. Comparison of 17-OHP concentrations between different gestational ages and birth weight categories indicated that gestational age and birth weight were negatively correlated with 17-OHP levels and gestational age had a greater effect on 17-OHP levels. Five pathogenic mutations(c.293-13C/A>G[IVS2-13C/A>G], c.955C>T[p.Q319*], c.1069C>T[p.R357W], c.923dupT and exon1[-113bpSNP]) and one novel mutation(exon6 deletion) were detected in five patients taken genetic test of *CYP21A2* gene mutations.

Conclusions: The incidence of CAH in Beijing was higher than the national average. c.293-13C/A>G mutation was the most common mutation associated with CAH in Beijing. Early detection and treatment through neonatal screening may reduce mortality rates and optimize developmental outcomes.

### P25. The Adjustment of Cut-off for Congenital Adrenal Hyperplasia Neonatal Screening by GSP According to Gestational Age and Age at Sample Collection

JiangXiangHuangYonglanFengYiTangFangGuangzhou Women and Children Medical Center, Guangzhou Newborn Screening Center, Guangzhou, China

Context: CAH screening is facing great challenges because of a high false positive rate and a low positive predictive value, we established and optimized the 17-OHP cut-off value for congenital adrenal hyperplasia neonatal screening with a genetic screening processor (GSP) according with gestational age (GA), birth weight (BW) and age at sample collection to improve newborn screening efficiency.

Method: The 17-OHP concentrations in dried blood spots were measured by time-resolved immunofluorescence with a GSP during from February 1st, 2018 to December 31th, 2018. The 17-OHP concentrations from 48,592 newborns were grouped and assessed in terms of gestational weeks(<32 weeks, 32–36 weeks, 36–37 weeks, 37–42 weeks and ≥42 weeks), birth weight (<1000 g, 1000–1500 g, 1500–2000 g, 2500–4000 g and ≥4000 g) and age at sample collection (24–48 h, 48–72 h, 72–168 h and ≥168 h). The 99.5th percentile was used to set an initial cut-off value as a reference.

Result: There were significant differences in 17-OHP concentrations among newborns with different GA and BW. The correlation between GA and 17-OHP was better according to regression analysis. A significant difference in the concentrations of 17-OHP among different collection age groups has been observed. Finally, we defined new multitier cut-off concentrations based on GA and age at sample collection. Application of the new cut-off values resulted in a 30% reduction of the positive rate and PPV increased from 1.11% to 1.56%, which was a 40% increase from before adjustment.

Conclusions: GA, BW and collection time were useful tools to adjust the cut-off value of CAH screening and the efficiency of CAH screening can be substantially improved by adjusting the multitier cut-off value according to GA and age at sample collection.

C. Phenylketonuria

### P26. The Clinical and Laboratory Study of the First Case of PCD deficiency in China and Literature Review

SunFangWangShunanLiXiaowenZhangZhixinShenMingChina-Japan Friendship Hospital, Pediatrics, Beijing, China

Objective To explore the clinical features and genetic defect of pterin-4α-carbinolamine dehydratase (PCD) deficiency, a very rare type of tetrahydrobiopterin (BH4) deficiency.

Methods The clinical data, laboratory tests and gene sequencing results of one case of PCD deficiency were retrospectively analyzed. The related literatures were reviewed.

Result The patient was first noticed by local hospital as his blood concentration of phenylalanine (Phe) was 4.2 mg/dL (252 μmol/L) during newborn screening, which was retested as 5.86 mg/dL when he was 29-day-old. His urine neopterin (NP) was 0.75 mmol/molCrn and biopterin (BP) was 0.12 mmol/molCrn. The patient was referred to our department when he was 67-day-old. He did not present yellow hair, mouse urine odor, eczema or dystonia. He was the second child of the family, his elder sister and his non-consanguious parents were healthy. His blood concentration of Phe was retested as 1.17mg/dL and tyrosine was 1.41mg/dL. Urine NP was 3.533 mmol/molCrn, BP was 0.758 mmol/molCrn; furthermore, 7-biopterin (7-Pri) was detected as 1.803 mmol/molCrn. His dihydropteridine reductase (DHPR) was 3.938 nmol/min·5mm disc. The patient was followed up monthly and his blood concentration of Phe was persistently less than 2mg/dL. Now he was 5 months old and developed properly as his peers. Gene sequencing revealed that there was a homozygous point mutation, c.129C>A (p.F43L) in *PCBD1* gene. The pathogenic mutation was predicted to result in PCD deficiency. Both of his parents were healthy heterozygous carrier.

Conclusion This patient was the first case of PCD deficiency in China and the homozygous point mutation in PCBD1 gene was unreported before at home and abroad. 7-Pri could be detected in urine sample. The clinical features were benign. The blood Phe would quickly increase after birth but could drop to normal level spontaneously. There is no medical intervention for the patient if the blood concentration of Phe was monitored less than 2mg/dL.

### P27. Mutation Analysis of Phenylalanine Hydroxylase Deficiency Gene in Shanxi

DongQinYangJianpingMuWenjuanZhuLingYanYaqiongXueJinjieWangWeipengChildren’s Hospital of Shanxi, Children’s Hospital of Shanxi, Taiyuan, China

Objective: In this paper, the gene of PKU patients with phenylalanine hydroxylase deficiency (PAHD) diagnosed in Shanxi province is analyzed to study the characteristics of genetic variation of patients in this region, based on which the accurate diagnosis of children and guidance of family reproduction would be improved to some extent.

Methods: Firstly, EDTA anticoagulation are extracted from patients with PKU and their parents in Shanxi Maternal and Child’ Hospital with informed consent as the analysis sample Secondly, through the Second Generation Sequencing Technology, all exons and non-coding regions of phenylketonuria-related genes are chosen to be the target genes, thereby the target genes being dealt with the method of exon detection to find out the pathogenic mutation gene points of the patients. At last, the point mutation patients and their parents are chosen to be the analysis sample and verified using the method of the first-generation Sanger sequencing. The sequencing process was completed by Beijing Zhiyin Oriental Translational Medical Research Center.

Result: At first, the sequencing was completed on a sample of 206 patients and 402 mutation types were detected. Among those patients, 3 mutations were detected in 8 cases, single mutation was detected in 18 cases and there also existed 2 cases that not only 2 mutations of PAH gene were detected but also a PTS mutation point was found. This means that the compound heterozygotes are predominant (87.86%). Secondly, 80.35% of the mutation regions were located on five exons, among which Exon 7 occupied the largest proportion (33.33%), followed by Exon 11 (14.43%), Exon 12 (12.19%), Exon 6 (10.95%) and Exon 3 (9.45%). Thirdly, the top five amino acid changes were p.AR243Q; P.EX6-96A > C; P.V399V; P.R111X; P.R403p. At last, among the mutations types, missense mutation was the most dominant mutation (17.21%), followed by shear site mutation (7.98%), nonsense mutation (13.18%), truncation mutation (14.93%) and deletion mutation (14.93%).

### P28. Studies on Mutations of Gene in Children with Phenylalanine Hydroxylase Deficiency in Qingdao

ZhangLiqinDuWeiLuWeibingQingdao Women and Children Hospital Affiliated to Qingdao University, Newborn Screening Center, Qingdao, China

To explore the mutation characteristics of phenylalanine hydroxylase (PAH) gene in children with PAH deficiency in Qingdao and provide scientific reference for prenatal diagnosis and treatment.

Method: Genetic analysis was performed on 43 children with PAH deficiency detected by neonatal screening in Qingdao and their parents. The PAH gene mutation site of the children was searched and the corresponding mutation site of their parents was detected and verified.

Result: (1) Two of the 44 children with PAH deficiency had homozygous mutations in the PAH gene and the frequency of homozygous mutations was 4.55%. All mutations were detected in their parents’ corresponding mutation sites. (2) A total of 37 mutations were found in 44 patients with PAH deficiency, of which the mutation frequency of c.728g > A was the highest (15.91%), followed by c.1068C>A (10.23%) and c.158G>A (9.09%) again. (3) There were 19 PAH gene mutations in 21 children with phenylketonuria (PKU), of which the mutation frequency of c.1068C>A was the highest (21.43%), followed by c.728G>A (19.05%). (4) There were 14 PAH gene mutations in 10 children with mild PKU, of which the mutation frequency of c.721C>T/722delG was the highest (15.00%), followed by c.1197A>T, c.1301C>A, c.721C>T, c.728G>A (10.00%). (5) There were 17 PAH gene mutations in 13 children with mild HPA, of which the mutation frequency of c.158G>A was the highest (26.92%), followed by c.728G>A (15.38%).

Conclusion: The mutation of PAH gene in children with PAH deficiency in Qingdao is mainly heterozygous mutation and has obvious hot spot mutations (c.728G>A, c.1068C>A, c.728G>A). Children with phenylketonuria were mainly c.1068C>A, c.728G>A, while children with mild PKU were mainly c.721C>T/722delG and children with mild HPA were mainly c.158G>A. The study of PAH gene mutations lays the foundation for the in-depth development of PAH deficiency genetic diagnosis, prenatal diagnosis and further gene therapy.

### P29. A Novel Homozygous Splicing Mutation of QDPR in a Case of Dihydropteridine Reductase Deficiency

YangBichengWangFengLuQingXuXiaolanJiangxi Provincial Maternal and Child Health Hospital, Neonatal Disease Screening Center, Nanchang, China

We report a case of DHPR deficiency and a novel homozygous splicing mutation of QDPR gene. The one-year ten-month old girl was the second child of an unrelated healthy Chinese couple. Pregnancy and birth were unremarkable. Newborn phenylketonuria screening revealed a negative result. The subject was admitted to hospital at the age of 22 months because she appeared symptoms including fever, cough, twitch and lethargy. On examination, she presented with global developmental delay and involuntary movements. She had ataxia, dystonia and oculogyric. Electroencephalography was abnormal. Tandem mass spectroscopy showed elevated phenylalanine level (202 μM, cutoff: <120 μM) and elevated ratio of phenylalanine to tyrosine (4.1, cutoff: <2.0). Despite supplementation of L-dopa/carbidopa and BH4 and correction of acidosis, the girl died on day 7 of treatment. Her older brother unexplainedly died from similar symptoms at 2 years of life. Blood samples from the girl and her parents were tested for genetic- and mutation analysis, after informed consent. Genetic analysis of the girl showed a novel homozygous splicing mutation (QDPR_ex 2 NM_000320.2: c.106-2A>G) in the QDPR gene. The father and mother showed heterozygous splicing mutation (QDPR_ex 2 NM_000320.2: c.106-2A>G) in the QDPR gene. Although newborn phenylketonuria screening is expected to enable presymptomatic diagnosis of DHPR deficiency. However, absence of hyperphenylalaninemia does not exclude DHPR deficiency. This patient was not detected by newborn phenylketonuria screening. DHPR deficiency affecting only the central nervous system does not lead to hyperphenylalaninemia was also described previously. Here we describe similar metabolic phenotype in this patient with a novel homozygous splicing mutation of QDPR. According the clinical outcome of the patient in this report, there was a certain correlation between the genotype and the phenotype.

### P30. Logistic Regression Analysis Improve Diagnostic Accuracy in Secondary MS/MS Newborn Screening for PKU

ZhuZhixingLuHuiTianGuoliGuJianleiWangYanminCaiXiaoshuGuoJingShanghai Children’s Hospital, Biomedical information center, Shanghai, China

Phenylketonuria (PKU) is one of the common inborn error of genetic metabolic disorder which affects nerve development and aberrant behavior usually become apparent with the child grows. In China, the incidence of PKU approximates 1:17000 [1]. Using the newborns screening can effectively control metabolic diseases such as PKU and patients who receive appropriate treatment in the early stage are the same as normal people. Currently, MS/MS screening for high-risk cases requires secondary testing and verification, such as urine testing or genetic testing. Newborn Screening Department of Shanghai Children’s Hospital has accumulated more than 100,000 newborns mass spectrometry screening since 2017 and 262 cases were recalled as high-risk groups, 50 cases were finally diagnosed after further examination. MS/MS can diagnose PKU more accurately. However, the data analysis is a big challenge for MS/MS data. Machine learning method has advantages in high-dimensional and complex features, which can choose the optimal subset to mine the metabolic patterns to reduce the recall rate without further testing and explore the discovery of new clues for unknown causal relations. In this study, the aim is to adopt the Machine learning method to perform secondary screening on PKU high-risk individuals, identifying false-positive samples and reduce the recall rate. Based on our observation, the LRA classifiers showed high accuracy performance with the mean ± SD of a specificity (Sp) of 94.20% ± 3.07%, an accuracy (Acc) of 90.03% ± 3.40% and an area under curve (AUC) of 92.64% ± 3.60%. The results show that the LRA model can be used to re-screen high-risk groups, greatly reducing the number of suspected cases and it has important reference for the selection and evaluation of the secondary screening method.

[1]. Lee, D.H.; Koo, S.K.; Lee, K.S.; Yeon, Y.J.; Oh, H.J.; Kim, S.W.; Lee, S.J.; Kim, S.S.; Lee, J.E.; et al. The molecular basis of phenylketonuria in Koreans. *J. Hum. Genet*. **2004**, *49,* 617–21.

### P31. Mild 6-Pyruvoyl-Tetrahydropterin Synthase (PTS) Deficiency with c.286G>A and c.84-291A>G Mutation: A Case Report

MaZhijunYangNanGongLifeiKongYuanyuanBeijing Obstetrics and Gynecology Hospital, Capital Medical University, Department of Laboratory Medicine, Beijing, China

Background: Hyperphenylalaninemia (HPA) is a hereditary metabolic disease. PTS is the key enzyme in BH4 synthesis. It is involved in the second step of the de novo biosynthesis of tetrahydrobiopterin (BH4), which is a vital cofactor of nitric oxide synthases and three types of aromatic amino acid hydroxylases. In this study, we report a patient with c.286G>A and c.84-291A>G mutations in PTS gene.

Study object: The patient is a female infant. On day 15 after birth, neonatal screening showed that the content of phenylalanine in blood was high. Then she was admitted to our hospital.

Diagnosis: No manifestations, such as skin whitening and yellowing, poor appetite, sucking weakness were detected. No specific odor was found in urine. The results of phenylalanine detection in blood showed that the content was 14.2 mg/dL. Thirty-six days after birth, the results of urine petrin spectrum analysis were as follows: N: 3.44mmol/molCr, B: 0.21mmol/molCr, B%: 5.65%. The DHPR analysis showed that the activity was 1.65nmol/min.5mmdisc (54.86%). The DNA sequencing results show that patient carried c.286G>A and c.84-291A>G mutations in PTS gene.

After 1 year of birth, the height, weight and intelligence of the patient were found to have developed normally. The phenylalanine content in the blood fluctuated within normal range.

Conclusion: PTS deficiency is the major cause in BH4 metabolic abnormality. After 1 year of observation, the patient was found to be in healthy condition, indicating that mutation c.84-291A>G primarily caused mild PTS deficiency.

Mutation c.84-291A>G was located in the intron of the PTS gene and affected the mRNA splicing process. The spliced mRNA had three lengths: normal length, exon 3 deletion and 79 bp fragment insertion. The low level of normal PTS protein in cells can perform their function.

In this case, we report that when c.84-291A>G and other mutations that caused severe PTS deficiency appeared on different chromosomes of the same patient, that patient would have mild PTS deficiency.

D. Glucose-6-Phosphodehydrogenase Deficiency

### P32. Ten-Year Retrospective Analysis of Screening for G6PD Deficiency in Neonates in Hainan Province

WangJieZhaoZhendongHainan Maternal and Child Health Hospital, Neonatal Disease Screening Center, Haikou, China

Objective: To investigate the prevalence and gene mutation of G6PD deficiency in Hainan population.

Methods: The G6PD activity of dried blood tablets of newborns born in Hainan Province from 2007 to 2016 was screened by fluorescence spot method. The G6PD/6GPD ratio method was used to confirm the diagnosis of suspected sample recall and the polychromatic probe fluorescent PCR melting curve method (MMCA) was used to genotype 3012 dry blood slices of children with partial diagnosis deficiency.

Results: From 2007 to 2016, 36,314 positive samples were screened in 914,520 neonates. 26,370 cases of G6PD deficiency were diagnosed. The prevalence of G6PD deficiency was about 3.97% (36,314/914,520) and 2.88% (26,370/914,520) in Hainan Province. The incidence of G6PD was 2.90% (22,156/761,365) in Han population, 2.80% (3278/117,058) in Li population, 2.91% (704/24,209) in Miao population and 1.95% (232/11,888) in other ethnic groups. (2) In 301 G6PD confirmed cases, 13 mutation types were detected by gene detection. The main mutation types were c.1376G>T, c.1388G>A, accounting for 76.56% of the total mutation. C.95A>G and c.1024C>T were also common, accounting for 15.18% of the total mutation and the combination of four mutations accounted for 91.74%. In addition, two mutations outside 16 genotypes, C.86C>T and C.1311C>T, were found by gene sequencing.

Conclusion: The incidence of G6PD among newborns in Hainan Province is high and there are ethnic and regional differences in G6PD incidence. (2) The main mutations were c.1376G>T, c.1388G>A, c.95 A>G and c.1024C>T.

**Keywords:** Hainan Province; dry blood tablets; incidence; G6PD deficiency; gene mutation

### P33. Chinese Newborn Screening for G6PD deficiency, from Enzyme Activity to Gene

ZouLinYuChaowenLiuZhidaiLiQinggeWangZhiguoChildren’s Hospital, Chongqing Medical University, Center for Newborn Screening, Chongqing, China

Glucose-6-phosphate dehydrogenase deficiency (G6PDd) is one of the most common X-linked enzymopathies caused which can cause fulminant hemolysis, severe hyperbilirubinemia, neonatal jaundice and even death. Newborn screening (NBS) is a public health program proved to be effective and economical way for G6PDd prevention around the world. However, little is known of NBS for G6PDd in China.

From 2013 to 2017, the screened population NBS G6PDd was increased from 3,957,075 to 5,199,160 and the screening organizations was increased from 71 in 12 provinces to 111 in 20 provinces. In 2017, the total incidence rate of G6PDd was 0.723%, the screening rates of G6PDd by province were between 37% and 100% and the incidence rate of G6PDd in each province ranged from 0.001% to 3.387%, lowest in Shanxi, highest in Guangxi, high in South China and low in North China.

We unbiased recruited 1,764,299 newborns from 12 provinces. After first-line screening by G6PD activity, G6PD mutation of 10,357 G6PDd suspected patients were identified, getting a mutation detecting rate of 98·8%. The top 5 most frequent G6PD mutations were c.1376G>T, c.1388G>A, c.95A>G, c.1024C>T and c.871G>A. c.487G>A was common in North China and c.592G>T and c.1360C>T were only found in the coastal regions, with the ethnic features of high incidence in Li, Zhuang and She groups. c.152C>T, c.290A>T, c.697G>C and c.1285A>G were firstly reported, causing the decline mRNA expression and G6PD activity.

We then analyzed another 6443 female and 7358 male newborns with normal G6PD activity in 8 provinces and identified 5.8% female heterozygotes but without male. 17.4% G6PD female heterozygotes were exhibited acute hemolysis and hyperbilirubinemia after two-year following-up. This work provides information and experience of Chinese NBS G6PDd, which is helpful for the other high incidence G6PDd regions of the world.

**Keywords:** newborn screening; G6PD deficiency; gene; mutation

### P34. Screening of Glucose-6-Phosphate Dehydrogenase Deficiency in Zhejiang Province

MiaoHiaxiaZhangTingFangKexinXuXiaochaWuDingwenLiQingboShiYezhenXuKeYangRulaiThe Children’s Hospital Zhejiang University School of Medicine, Genetics and Metabolism, Hangzhou, China

Objective: To explore the epidemiological distribution characteristics of glucose-6-phosphate dehydrogenase(G6PD), incidence of G6PD deficiency in neonates and to determine its cutoff values.

Methods: All newborns (*n* = 1.44 million) born in Zhejiang Province (except Ningbo) from March 1, 2015 to September 31, 2017 were included in this study. The results were analyzed by using percentile method, one-way analysis of variance and R×C contingency tables by SPSS 22.0.

Results: Significant difference was found in G6PD level among groups of different gestational age, blood sampling age and blood sampling season. G6PD activity was highest in the <36 weeks gestation group and lowest in the >42 weeks gestation group; The group of blood sampling age of more than 28 days had the lowest G6PD level. The G6PD level of summer and autumn are lower than winter and spring. The male incidence of G6PD deficiency was significantly higher than female incidence. In different regions of Zhejiang province, incidence of G6PD deficiency basically showed a trend of high in the south and low in the north. Significant difference was not found among different gender, birth weight and districts.

Conclusions: The G6PD level is affected by gestational age, blood sampling age and blood sampling season. The incidence of G6PD deficiency was influenced by gender and different regions. The male and female cutoff values were initially set at 2.6 U/g Hb.

### P35. Exploration of the Newborn Screening and Gene Mutation of Glucose-6-Phosphate Dehydrogenase in Sichuan

ZhouJingyaoSichuan provincial Hospital for Women and Children, Newborn screening center, Chengdu, China

Objective To analyze the glucose-6-phosphate dehydrogenase level and to reveal the molecular epidemiologic characteristics of gene in Sichuan area.

Methods G6PD enzyme activities were measured by quantitative fluorescence assay in dry blood spots of 229091 newborns in Sichuan neonatal screening center during 2014 to 2017. Genetic analysis of G6PD gene and enzyme diagnosis were performed on the dry blood spot samples of 182 cases who with high risk of screening.

Results (1) Using the cutoff value of G6PD < 2.8 U/g Hb, a total of 1169 cases (0.51%) showed positive screening results in 229,091 newborns, including 1028 (0.87%) males and 141 (0.13%) females. (2) Among the newborns with positive screening results, 153 males and 29 females were randomly chosen for G6PD gene analysis. The results showed that 122 (84.72%) males were hemizygote and 9 females (23.68%) females were found to be variant. Five common mutations were c.1376G>T, c.1388G>A, c.1024C>T, c.95A>G, c.871G>A, accounting for 91.06% of detected alleles.c.1388G>A gene mutation was the main gene mutation type in Sichuan area. G6PD gene sequencing was performed on 51 newborns who with positive screening results but no hot spot mutation. 5 males were found with mutation. The mutations were c.406C>T, c.703C>T, c.835A>T and c.1311C>T1. (4) Based on results of G6PD gene analysis, the incidence among male infants was 0.76% and among female infants was 0.03% in Sichuan area. (5) Compared with G6PD gene diagnosis, the sensitivity of G6PD activity assay in red blood cells were 90.44%, male 93.70% and female 44.45%; and PPV were 85.42%, male 87.5% and female 50%.

Conclusions The majority of male children who with G6PD deficiency can be detected by the newborn screening program and the enzyme diagnostic criteria in Sichuan area. Combined with molecular analysis will improve the accuracy of diagnosis of G6PD deficiency and detect more heterozygous females.

### P36. Characterization of Mutations through Whole Gene Sequencing of the Glucose-6-Phosphate Dehydrogenase Gene among Filipino Children with G6PD Deficiency

Cutiongco-de La PazEva MariaPadillaCarmencitaRainier SantosNickBaluyotMelissa MaeUniversity of the Philippines Manila, Institute of Human Genetics, National Institutes of Health, Ermita Manila, Philippines

Background. Glucose-6-phosphate dehydrogenase (G6PD) deficiency is known globally as the most common enzyme deficiency, estimated to affect 400 million people worldwide. This multi-ethnic disease has been shown to be more prevalent in tropical and subtropical regions where malaria is endemic. In the Philippines, one out of every 52 newborns is confirmed to have G6PD deficiency. G6PD is a key catalytic enzyme in the hexose monophosphate pathway. In the Philippines, the diagnosis is confirmed through a red blood cell-based semi-quantitative enzyme assay. However, this assay is unable to classify the patients according to severity and makes genetic counseling and management challenging. Deficiency in the G6PD enzyme is secondary to mutations in the G6PD gene located on band Xq28 on the long arm of the X chromosome. Several studies have been published that showed a variety of mutations in the G6PD gene. A reliable, rapid and affordable genotyping method is valuable for better management and more accurate genetic counseling of patients and families with this condition.

Methods. This is a descriptive study that describes the mutations detected by whole gene sequencing of the G6PD gene among 106 Filipino children aged 0–12 months old with G6PD deficiency.

Results and Discussion. The most common variant is Viangchan, which is also the most common variant in the Indochina region, involves a G to A mutation at nucleotide 871 on exon 9. Union, the 2nd most common variant, a c1360C>T mutation on exon 11, was initially reported in an individual from the Philippines but has now been found to have a worldwide distribution. The Chatham variant, c1003G>A in exon 9, is the 3rd most common followed closely by the Vanua Lava variant.

Conclusion. The study characterized the three most common mutations in the G6PD gene among Filipinos with G6PD deficiency. Results of the study will serve as the springboard for the development of a low cost confirmatory molecular based assay that can detect most of the common G6PD mutations in the Filipino population.

### P37. External Quality Assurance Program of G6PD Confirmatory Centers in the Philippines

Dizon-EscorealMaria TrudaSombongAnna VictoriaJomentoCharityAsuncion PanolKarenUniversity of the Philippines Manila, Newborn Screening Reference Center, National Institutes of Health, Manila, Philippines

Background: Under the National Comprehensive Newborn Screening Program of the Philippines, one of the Newborn Screening Reference Center (NSRC) mandate is to maintain an external laboratory proficiency testing program. The NSRC established its External Quality Assurance Program (EQAP) for G6PD Confirmatory Centers (G6PDCCs) by partnering with Preventive Medicine Foundation Taiwan. The G6PDCCs are required to enroll and to participate in the quarterly proficiency survey. In 2009 the EQA performance of the nine G6PDCCs ranges from 15-24% coefficient of variation (CV). Over the years, various strategies were employed to address the gaps encountered. In 2018, inter-laboratory performance has improved to < 10% CV.

Objective: This presentation aims to report on: 1) EQAP for G6PDCCs, 2) the performance of the G6PDCCs over the years, 3) challenges encountered and solutions applied to address them and 4) current implementation challenges.

Methodology: Ten-year EQAP and monitoring reports were reviewed to present trends in G6PDCC performance, identify challenges and actions taken.

Results and Discussion: In 10 years, the EQAP for G6PDCCs saw major improvements: 1) standardization of G6PDCC setting-up process, 2) development of checklist in setting up of G6PDCCs, 2) standardization of testing process in coordination with service provider, 3) improvement of the monitoring system of G6PDCCs with unsatisfactory results and 4) improvement in the reliability of confirmatory results. Challenges met include: 1) compliance of G6PDCCs to NSRC standards, 2) unavailability of G6PD reagents and controls and 3) lack of laboratory staff training on test procedures and interpretation of EQAP results.

### P38. A Rapid Multiplexed Primer Extension Method Using Dried Blood Spot Samples as the Second-tier Assay for Newborn Screening of Glucose-6-Phosphate Dehydrogenase Deficiency

LiuTze-TzeChenHsiao-JanChangYing-ChenLiuYu-NingKaoShu-MinLiuMei-YingWengYing-YenHsiaoKwang-JenChiuYen-HuiNational Yang-Ming University, Cancer Progression Research Center, Taipei, Taiwan

Background: The X-linked glucose-6-phosphate dehydrogenase (G6PD) deficiency is highly prevalent in Southeast Asians and the overall incidence in Taiwan was found to be about 2%. The enzyme activity of female carrier of G6PD deficiency may range from deficiency to normal due to lyonization, only 15–20% of female carriers could be detected in current newborn screening program.

Methods: A multiplex PCR coupled with a single-base primer extension method, a multiplex-SNaPShot assay, was adapted to detect 21 common G6PD mutations found in Taiwan and Southeast Asia using dried blood spot samples. Subjects selected: (1) 500 G6PD-deficient males confirmed by quantitative enzyme activity assay; (2) 90 individuals with G6PD activity >10.0 U/gHb in newborn screening as unaffected controls (52 males and 38 females); and (3) 825 individuals with G6PD activity of 4.1–10.0 U/gHb in newborn screening (141 males and 684 females).

Results: (1) By evaluating in 500 confirmed G6PD deficient baby males, the detection rate of this multiplex-SNaPshot assay was 98.2% (491/500). For the undetermined 9 mutant alleles (1.8%), other mutations were identified by sequencing. (2) of 90 samples (activity > 10.0 U/gHb), no mutation was detected which indicating a high specificity of this assay. (3) of 684 female newborns in the group with G6PD activity of 4.1–10 U/gHb, we detected 419 heterozygotes and 1 homozygote. of 141 male newborns, 71 were G6PD hemizygotes. Among which, 67 baby males had activity of 4.1–6.0, while 4 baby males with activities of 6.1–7.0 U/gHb carried mild mutations. This observation indicated some G6PD-deficient male newborns would be missed when the cut-off was set at 6.0 U/gHb.

Conclusion: To increase the detection rate of newborns at-risk of G6DP deficiency, we suggest adjusting the cut-off value of 6.0 to 7.0 U/gHb and applying the multiplex SNaPshot assay as the second tier test for all newborns whose G6PD activities are between borderline range (5.1–7.0 U/gHb) in newborn screening to reduce clinic referrals.

### P39. Newborn Screening for G6PD Deficiency: Pilot Study to Expand the Screening Panel in Sri Lanka

HettiarachchiManjulaAmarasenaSujeewaUniversity of Ruhuna, Nuclear Medicine, Galle, Sri Lanka

Introduction Glucose-6-phosphate dehydrogenase (G6PD) is an important enzyme in the hexose monophosphate oxidative pathway where it plays an essential role in reducing nicotinamide adenine dinucleotide phosphate (NADP) to NADPH. Individuals with partial G6PD deficiency has been seen frequently in certain parts of Asia and the Mediterranean region. This investigation was aimed to assess the feasibility of successfully performing G6PD screening on DBS samples and incorporation of G6PD screening to the screening panel.

Methods This study was done in 4 out of 15 districts where the newborn screening for congenital hypothyroidism has been running for over 5 years. Dried blood spot samples received during 2 calendar months were subjected to the initial analysis of G6PD enzyme levels. The G6PD activity of the blood sample is calculated by comparing the rates of the sample to rate of the normal control with known G6PD activity and measuring the hemoglobin of the sample (Hb Normalization) at the OD of 405 nm. All babies with residual activity range U/g Hb < 2.5 during initial screening were informed and recalled a second dried blood spot sample after 6 months. These samples were analyzed using the similar assay protocol in order to validate the G6PD enzyme activity.

Results Out of 8795 dried blood spot samples, 215 samples with residual activity range U/g Hb <2.5 on initial analysis. Analysis of second samples revealed that 58 babies (36 males and 22 females) continued to have <2.5 Ug Hb activity. The incidence of G6PD deficiency during this study period in this selected population was 1 in 155. The range of the measured G6PD activities was 0.5–13.8 U/g Hb. Inter assay variation was 2.7% (13.81 U/g Hb) and 2.8% (3.16 U/g Hb).

Discussion We are reporting the incidence of G6PD deficiency for the first time among Sri Lankan neonates. We believe that newborn screening is an area which would greatly benefit from the implementation of this procedure.

### P40. Analysis of G6PD Gene Mutation in Newborns in Hainan Province

ZhaoZhendongWangJieLiuXiulianHuangCidanHainan Maternal and Child Health Hospital, Neonatal Disease Screening Center, HaiKou, China

Purpose: Looking for the incidence of G6PD deficiency and genetic mutations in newborns in Hainan.

Method: The G6PD activity of 130,512 neonatal dried blood tablets born in2017 in Hainan was screened by fluorescence analysis. The suspicious samples were recalled in preliminary screening and confirmed the diagnosis by G6PD/6GPD ratio method. And the multicolor melting curve analysis (MMCA) was used to genotype the dried blood tablets of undiagnosed children.

Result: (1) In 2017, among the 130,512 neonates, 5221 were screened positive in preliminary screening and 2,993 cases of G6PD deficiency were diagnosed. The incidence of G6PD deficiency in Hainan population was about 4.00% (5221/130,512), 1.80% (1792/109,590) for Han Nationality and 5.28% (934/17,698) for Li Nationality. (2) The mutations were detected in 2993 newborns, which the mutation rate is 2.29% (2993/130,512), 1.80%(1972/109,590) for Han Nationality and 5.28% (934/17,698). (3) The G6PD gene mutation types of newborns in Hainan are abundant, a total of 13 gene mutation types were detected. The main mutations were c.1376 G>T and c.1388 G>A, accounting for 76.68% (54.66% +22.02%) of the total mutations. c.95A>G and c.1024C>T were also common, accounting for 15.31% (8.49% +6.82%) of the total mutations. One mutation was found in C.86C>T, C.487G>A and C.1004 C>A, which is the first report in Hainan population.

Conclusion: The incidence of G6PD in neonates in Hainan Province is high and the Li nationality is higher than the Han nationality. The genetic mutations are mainly c.1376G>T, c.1388G>A, c.95A>G and c.1024C>T, Han nationality and There are differences in the types of mutations in the G6PD gene in Li.

E. Methylmalonic Acidemia, Propionic Acidemia

### P41. Screening and Gene Mutation Analysis of Neonatal Methylmalonate Acidemia in Henan

ZhaoDehuaLiXiaoleThe Third Affiliated Hospital of Zhengzhou University, Newborn screening Center of Henan Province, Zhengzhou, China

Objective: To investigate the characteristics of neonatal methylmalonic aciduria (MMA) regarding clinical manifestations, laboratory findings, gene mutation.

Methods: Acylcarnitine spectrum screening was performed on 720,667 newborns in Henan from January 2013 to December 2017 by using liquid chromatography tandem mass spectrometry. The abnormal results were confirmed by detecting organic acids in urine with GC-MS and gene sequencing analysis. Patients with isolated MMA were treated with dietary control and levocarnitine, while those complicated by homocysteinemia were treated with VB12, levocarnitine, betaine and folinate.

Result: MMA was diagnosed in 148 patients, among which 15 patients were isolated MMA and 133 patients were complicated by homocysteinemia. The fourteen isolated MMA cases failed to response to vitamin B12 treatment without any symptoms on diagnosis and 1 case had died before diagnosed. However, vitamin B12 was effective for other133 patients, among which 117 had no symptoms on diagnosis and sixteen had manifestations such as slow response, vomiting, poor feeding and jaundice. All patients underwent genetic analysis and fifteen were found with MUT gene mutations, 131 cases with MMACHC gene mutations and 2 cases with normal gene mutation. MUT gene mutations were classified into 20 types, c.1663G>A was the most common gene mutation, followed byc.729-730insTT.

c.431G>A, c.494A>G and c.965T>A were new gene mutation types. 35 types of MMACHC gene mutations were identified and the most common type is c.609G>A, followed by c.482G>A. We also found new gene mutation types such as c.277-5-301del, c.311C>T, c.321G>A, c.420dupG, c.440G>A, c.540-547delTGACTGTG and c.616C>T.

Conclusions: Newborn screening with liquid chromatography tandem mass spectrometry is important for early diagnosis of MMA. The incidence of neonatal MMA is 1:4869. MMACHC gene defects are the main causes of MMA in Henan and the predominant one is c.609G>A mutation.

### P42. Clinical, Biochemical and Genetic Pictures of Propionic Acidemia Patients Detected by Neonatal Screening in Japan

TajimaGoMaedaYasuhiroFukaoToshiyukiNational Center for Child Health and Development, Division of Neonatal Screening, Tokyo, Japan

Background: Propionic acidemia (PA) and methylmalonic acidemia (MMA) are the most representative organic acid disorders caused by inborn errors of valine and isoleucine metabolism. Before tandem mass spectrometry (TMS)-based neonatal screening (NS) was introduced, the frequency of symptomatic PA was estimated to be 1/465,000 in Japan, which was a half of that of MMA. On the other hand, pilot study of TMS-NS detected twice as many patients of PA as those of MMA, with the frequency reaching 1/45,000. Genetic study indicated that this discrepancy between frequencies of symptomatic PA patients and those detected by TMS-NS was caused by a particular variant of PCCB, c.1304T>C (p.Y435C).

Methods: To reveal actual conditions of PA patients found by TMS-NS, we conducted a nationwide questionnaire study. Activity of propionyl-CoA carboxylase (PCC) was measured by quantifying conversion of propionyl-CoA into methylalonyl-CoA by crude lysates of lymphocytes.

Results: Our questionnaire study detected 87 NS-patients and revealed that none of them presented with symptoms related to PA. Forty-one patients were proved to be homozygotes of p.Y435C variant. Twenty patients had p.Y435C on one allele, of whom five had a pathogenic variant p.T428I on the other allele. Enzymatic activity of p.Y435C variant PCC was estimated to be 10% to 20% of that of wild type enzyme. Comparatively, none of 15 symptomatic patients detected by the same study harbored p.Y435C allele. Among various symptoms, cardiac complications (cardiomyopathy and long QT) were documented in 7 of them, whether they had suffered severe acidotic crisis or not.

Conclusion: One allele of p.Y435C variant seems enough to protect NS-positive patients against clinical symptoms. However, it is difficult to exclude latent influence of the biochemical abnormalities on their cardiac function, which may need life-long observation.

Acknowledgements: This research was supported by AMED under Grant Number JP17ek0109276.

### P43. Genotype Analysis of 566 Patients with cblC Type Methylmalonic Acidemia

HanLianshuEHuishuXinhua Hospital, Shanghai Jiaotong University School of Medicine, Department of Pediatric Endocrinology and Genetic, Shanghai Institute for Pediat, Shanghai, China

Objective: To explore the genotype-phenotype correlation in patients with cblC-type methylmalonic acidemia(MMA).

Methods: Retrospective analysis of clinical data of 566 patients with cblC-type MMA caused by mutation of MMACHC gene in our hospital from August 2009 to January 2019, figure out the relationship between genotypes and age of onset, disease characteristics and treatment effect.

Results: of the 566 patients, 195 (34.5%) cases were from neonatal screening and 52 of them had an onset. Among 371 unscreened patients, 341 cases were clinical patients and 30 cases were diagnosed after their siblings’ onsets. The remaining. MMACHC gene tests showed that c.609G>A (p.W203X) was the most common mutation, followed by c.658-660delAAG (p.K220del), 80A>G (p.Q27R), c.567dupT (p.I190Yfs*13) and c.482G>A (p.R161Q). These mutations frequency were 32.95%, 13.07%, 7.69%, 6.27% and 5.30%, respectively. Patients with p.W203X, p.K220del and p.I190Yfs*13 mutations were more likely to present with feeding difficulty, mental/motor retardation and lethargy; while patients with p.R161Q mutations mainly presented with decreased academic performance, memory loss, poor expression and decreased exercise capacity. All patients were vitamin B12-responsive. Follow-up study showed that among the newborn screening patients, 150 (76.9%) were healthy, 26 (13.3%) had development delay and 4 (2%) were dead; only 91 (23.2%) of the clinical patients recovered well, while 204 (51.9%) of them remained intelligence and/or motor disabilities and 41 died (10.4%).

Conclusions: The p.W203X is the hot spot mutation in Chinese cblC-type MMA. p.W203X, p.K220del and p.I190Yfs*13 are associated with early onset MMA, while p.R161Q have a relationship with late onset MMA and p.Q27R may be associated with pulmonary hypertension. Patients with p.R161Q have a better response to hydroxocobalamin than other mutations.

### P44. The Phenotypes and Genotypes of 851 Chinese Patients with Combined Methylmalonic Aciduria and Homocystinuria

KangLuluLiuYupengLiuYiHeRuxuanJinYingLiMengqiuSongJinqingZhangYaoDongHuiLiuXueqinYanHuiZhangChunyuXiaoHuijieLiHaixiaYangHongyunQinJiongZhengHongChenYongxingLiDongxiaoWeiHaiyanZhangHuifengZhangYananDengXinLiXiaowenHuangMinLiPeiZhaoZijiangYangYanlingPeking University First Hospital, Department of pediatrics, Beijing, China

Background: Combined methylmalonic aciduria and homocystinuria accounts about 70% of Chinese patient with methylmalonic aciduria. This study aimed to investigate the clinical, biochemical and genetic features of Chinese patients with combined methylmalonic aciduria and homocystinuria.

Method: Eight hundred and fifty-one patients with combined methylmalonic aciduria and homocystinuria from twenty-six provinces of China were enrolled in this study. Their main clinical symptoms, biochemical findings and genetic defects were studied.

Results: Among the 851 patients, 475 (55.82%) were males. 376 (44.18%) were females. The age of diagnosis ranged from several hours of life to 50 years old. The mean age at diagnosis was 1.80±0.14 years. 66 cases (7.75%) were detected by neonatal screening. 785 cases (92.20%) were clinically diagnosed after onset. Neuropsychiatric impairment was the most finding (49.80%), followed by hematological involvement (32.81%), cardiovascular diseases (7.58%), renal damage (8.63%) and skin lesion (7.58%). of 598 patients who were confirmed by genetic analysis, 595 cases (99.5%) had MMACHC mutations. HCFC1 mutations were found in two patients., ABCD4 mutations were identified in one case.

Conclusion: cblC type due to MMACHC gene defect is the main cause of combined methylmalonic aciduria and homocystinuria in China. Neonatal screening can significantly improve the outcome of patients. Differential diagnosis by chemical and genetic analysis are important.

### P45. Clinical and Gene Mutation Analysis of Methylmalonicacademia cblA Type in 11 Children

EHuishuHanLianshuXinhua Hospital, Shanghai Jiaotong University School of Medicine, Department of Pediatric Endocrinology and Genetic, Shanghai Institute for Pediat, Shanghai, China

Objective To explore the clinical manifestations, mass spectrometry results, genotypes and treatment outcome of cblA-type methylmalonic academia (MMA).

Methods: A total of 11 cblA type patients’ clinical features, laboratory findings and gene mutations were analyzed. Vitamin B12 (hydroxocobalamin), L-carnitine and special milk free of leucine were used as the main treatment.

Results: Vomiting, dyspnea and drowsiness were the major clinical characterizations of MMA cblA-type. After treatment, the propionylcarnitine level, ratio of propionylcarnitine/acetylcarnitine in blood, methylmalonic acid and methylcitric acid levels in urine have decreased significantly, with the media values respectively reduced from 11.90 µmol/L, 0.56, 145.00 mmol/molCr and 12.25 mmol/molCr to 4.22 µmol/L, 0.21, 39.37 mmol/molCr and 2.04 mmol/molCr (*p* < 0.05). Follow-up prognosis showed that 8 cases had normal development, while 3 cases revealed various degrees of mental or movement delay. Thirteen MMAA gene mutations were detected, with c.365T>C (p.L122P) being the most common (31.8%). Six novel mutations includedc.54delA (p.A19Hfs*43),c.275G>A (p.G92D), c.365T>C (p.L122P), c. 456delT (p.G153Vfs*8), c.1114C>T (p.Q372X) and c.1137_1138delCA (p. F379fs*36) were found.

Conclusion: The main clinical manifestations of patients with MMA cblA-type are vomiting, dyspnea and drowsiness. Using hydroxycobalamina as the main therapeutic method is recommend. The c.365T>C is a potential hot spot mutation of MMAA gene in China.

### P46. Clinical Analysis and Outcome of Patients with c.482G>A Variant in cblC Type Methylmalonicacidemia

HanLianshuEHuishuXinhua Hospital, Shanghai Jiaotong University School of Medicine, Department of Pediatric Endocrinology and Genetic, Shanghai Institute for Pediat, Shanghai, China

Objective To explore the clinical manifestations, treatment and outcome of patients with c.482G>A (p.Arg161Gln) variant in cblC type methylmalonic acidemia(MMA).

Methods The clinical manifestations, mass spectrometry results, genotypes, treatment and outcomes of 75 patients with cblC type MMA carrying c.482G>A (p.Arg161Gln) variant were analyzed.

Results: of the 75 patients, 57 (76%) were from newborn screening and one of them had an onset. Among the rest 18 unscreened patients, 2 were diagnosed after their full sisters’ or brothers’ diagnosis, the rest were clinical patients. There were 17 clinical patients, the medium age of onset was 12years old (10 days~26 years old), 12 late onset patients (70.6%) presented with poor academic performance, memory loss, poor expression and decreased exercise capacity, while 5 early onset patients (29.4%) presented with convulsion and development delay. All patients were vitamin B12-resiponsive. The blood level of propionylcarnitine, the ratio of propionylcarnit into acetylcarnitine, the urinary level of methylmalonic acid, Methylcitric acid and plasma homocysteine deceased significantly after treatment (*p* < 0.01). All patients diagnosed from newborn screening had normal development. However, only 3 clinical patients had a rather normal outcome and the others remained different levels of intelligence and/or motor dysfunction after treatment.

Conclusion The c.482G>A (p.Arg161Gln) variant of MMACHC gene is associated with late onset cblC type MMA. Patients with this variant have a better response to hydroxocobalamin than other variants. The outcome of patients diagnosed from the newborn screening is good, while once symptoms occur, the disability rate is often high. Therefore, newborn screening is a recommend method to prevent this disease.

### P47. The Phenotypes and Genotypes of Chinese Patients with Propionic Acidemia

LiuYiDingYuanLiDongxiaoKangLuluJinYingZhangYaoSongJinqingDongHuiLiHaixiaYangYanlingPeking University First Hospital, Department of Pediatrics, Beijing, China

Objective Propionic acidemia (PA) is a rare autosomal recessive disorder caused by deficiency of propionyl CoA carboxylase. To date, there is lack of large cohort study on Chinese patients. Thus, we investigated the clinical and genetic features of 60 Chinese PA patients and discussed the genotype-phenotype correlation.

Methods 60 patients from 16 provinces and clinically diagnosed between Jan 2007 and Oct 2018 were enrolled. Next-generation sequencing (NGS) was conducted for the genetic diagnosis. Variants were confirmed by Sanger sequencing and multiplex ligation-dependent probe amplification (MLPA). Amniotic fluid metabolites analysis and amniocyte mutation analysis were performed for the fetuses of the families with definite diagnosis.

Results Among the 60 patients, only 5 (8.3%) were detected by newborn screening. 55 (91.7%) were clinically diagnosed after onset. The onset age varied from 2 hours of life to 3.5 years. 25 cases were early-onset and 30 were late-onset. The common presentations were lethargy, hypotonia, vomiting, feeding difficulties, developmental delay, seizures, metabolic acidosis and hyperammonemia. The common MRI findings were bilateral basal ganglia damage and white matter changes. 5 cases detected by newborn screening had normal development, while the other 55 had varied neuropsychiatric disorders. 9 cases (15%) died. Gene tests were conducted in 58 cases. 24 had PCCA mutations and 34 had PCCB mutations. 30 mutations (11 reported and 19 novel) in PCCA and 30 mutations (15 reported and 15 novel) in PCCB were identified. Certain mutations seemed to be related to early- or late-onset type. Prenatal diagnosis was performed for 7 fetuses, with 3 wild-types and 4 carriers.

Conclusions The clinical manifestations of propionic acidemia are complex. Most patients diagnosed after onset had poor prognosis. Newborn screening is key for early diagnosis and treatment. 19 novel mutations in PCCA and 15 novel mutations in PCCB were identified, while the genotype-phenotype correlation is still unclear.

### P48. Gene Mutation Analysis in 98 Propionic Acidemia Patients

LiangLiliHanLianshuXinhua Hospital, Shanghai Jiaotong University School of Medicine, Department of Pediatric Endocrinology and Genetic, Shanghai Institute for Pediat, Shanghai, China

Objective: To analysis the clinical data and gene mutations of children with propionic acidemia (PA) in China and to discuss the relationship between clinical characteristics and gene mutations.

Methods: The subjects were 98 children with PA diagnosed from February 2002 to April 2018. The correlation between their clinical manifestations and gene mutation were analyzed.

Results: Among the 98 children with PA, 38% had early onset, the youngest was 2 days after birth and the oldest was 6 years and 3 months. 9 patients had no clinical manifestations were diagnosed by neonatal screening. Most of the first symptoms were conscious disturbance, vomiting, acidosis, in appetence and convulsions. Genetic mutations were detected in 93 children, 30 of which were PCCA mutations (31.9%) and 64 were PCCB mutations (68.1%). There were 40 mutations in PCCA, 27 of which were not reported in the literature. The mutation c.2002G>A was a homozygous mutation in 1 child and 6 children were carriers, accounting for 13.3% of the PCCA Allele. There were 54 mutations in PCCB, 38 of which were not reported in the literature. The mutation c.1087T>A was a homozygous mutation in 3 children who had no blood relationship and 15 children were carriers, accounting for 10% of the PCCB Allele. There was no significant correlation between clinical manifestations and gene mutation types.

Conclusions: We have detected 104 mutations in PCCA gene and PCCB gene, 65 of them were not reported in the literature. c.2002G>A may be a focus mutation of PCCA gene and c.1087T>C, c.838dupC, c.167_179del13ins1 may be focus mutations of PCCB gene in children with PA in China. There was no correlation between clinical manifestations and gene mutation types.

### P49. Genotypes of 1160 Patients with Methylmalonic Acidemia

EHuishuHanLianshuXinhua Hospital, Shanghai Jiaotong University School of Medicine, Department of Pediatric Endocrinology and Genetic, Shanghai Institute for Pediat, Shanghai, China

Objective: Analysis of the genotypes of patients with methylmalonic acidemia (MMA) to help clinician shave a better understanding of this disease.

Methods: A total of 1160 patients who were treated in our hospital from December 2003 to January 2019 were retrospectively analyzed to figure out the genotypes of MMA.

Results: Among 1160 patients, 878 cases (75.7%) were diagnosed with MMA combined homocysteinemia and 282 cases (24.3%) were diagnosed with isolated MMA. For patients with MMA combined homocysteinemia, 876 cases (99.8%) caused by MMACHC gene mutation and 2 patients (0.2%) by HCFC1 gene mutations. For patients with isolated MMA, 269 (95.4%) cases caused by MUT gene mutations, 12 (4.5%) cases by MMAA gene mutation and 1 case had MMAB gene mutation. A total of 77 MMACHC gene mutations were found, c.609G>A (p.W203X) mutation is the most common(43.04%), followed by c.658-660delAAG (p.K220del) (12.61%), c.80A>G (p.Q27R) (7.13%), c.567dupT (p.I190Yfs*13) (6.16%) and c.482G>A (p.R161Q) (5.37%). Patients with c.609G>A(p.W203X) mutation were more likely to present with mental/motor retardation, while patients with c.482G>A (p.R161Q) mutation mainly presented with late-onset MMA and often had better outcome. A total of 127 MUT gene mutations were found, c.729_730insTT (p.D244Lfs*39) is the most common(10.03%). 13 cases (25.00%) with c.729_730insTT (p.D244Lfs*39) died and 25 cases (55.77%) had an onset within one month, manifested with vomiting, feeding difficulties and lethargy.

Conclusion: The mutation of MMACHC gene is the main cause of MMA in China and c.609G>A is a hot spot mutation. The patients with c.609G>A (p.W203X), c.658-660delAAG (p.K220del) and c.567dupT (p. I190Yfs*13) mutations are associated with early-onset MMA, while patients with c.482G>A (p.R161Q) are associated with late-onset MMA and often had better outcome. The MUT gene mutation is most common in isolated MMA patients and the c.729_730insTT (p.D244Lfs*39) is the most common mutation.

### P50. Newborn Screening for Methylmalonic Acidemia in Henan Province by Tandem Mass Spectrometry

NiMinWangLiwenLiuSunaJiaChenluLiXiaoleMaShengjuLiYanruOuyangYunjiaMaKunZhaoDehuaThe Third Affiliated Hospital of Zhengzhou University, The Neonatal Screening Center of Henan Province, Zhengzhou, China

Aims: To investigate the effect of tandem mass spectrometry in the newborn screening for methylmalonic acidemia (MMA) in Henan province.

Methods: From December 2012 to December 2018, 882310 cases of newborns in Henan province who were fully breast-fed 72 hours after birth were collected and treated with sufficient heel blood to make dry blood filter papers. They were tested by tandem mass spectrometry for propionyl carnitine (C3), free carnitine (C0), acetyl carnitine (C2), methionine (MET), C3/C2, C3/C0 and C3/MET. Urine GC-MS was used to detect the changes in urine levels of MMA, 3-hydroxypropionic acid and methylcitric acid. Blood homocysteine and gene detection were also combined to make the diagnosis.

Results: A total of 174 cases of MMA were confirmed. Results of tandem mass spectrometry showed that 144 cases had increased C3, 93 cases had low C0 and 132 cases had low C2. There were 171 cases of increased C3/C2, 147 cases of increased C3/C0 and 135 cases of decreased MET. There were 148 cases of increased C3/MET. There were 169 cases of increased urinary MMA, 104 cases of increased methylcitric acid and 69 cases of increased 3-hydroxypropionic acid. There were 156 cases of increased homocysteine and the genetic test showed that 156 cases were MMACHC type, which was B12 effective type. The mutation frequency of 609G>A was the highest. Eighteen cases were MUT type which was B12 invalid type.

Conclusion: MMA is the most common organic acidemia in newborn genetic metabolic disease screening in Henan Province, with the incidence of 1/5070. The increase in blood C3/C2 and urine MMA was more specific for the diagnosis of MMA. The increase in C3/MET and homocysteine was good indication for the genotype of MMACHC which was B12 effective type. Through tandem mass spectrometry screening, most children can be screened before symptoms and can be effectively treated before genetic results.

**Keywords:** neonatal disease screening; tandem mass spectrometry; Methylmalonic Acidemia; genotype

### P51. Preliminary Application of Second-Tier Test Used in Newborn Screening by Tandem Mass Spectrometry in Zhejiang Province, China

HuZhenzhenYangJianbinHuangXinwenYangRulaiHuLingweiZhangChaoShangShiqiangWuDingwenZhangYuZhaoZhengyanThe Children’s Hospital Zhejiang University School of Medicine, Genetics and Metabolism, Hangzhou, China

Objective To investigate the feasibility of second-tier test used in newborn screening by tandem mass spectrometry in Zhejiang province, China.

Methods Between June 2017 and September 2017, 145,444 neonates were screened by tandem mass spectrometry (primary screening) at the Zhejiang newborn screening center. 239 dried blood spots of neonates that were reported as screen positive for methylmalonic acidurias or propionic acidemia based on abnormal propionylcarnitine or propionylcarnitine/acetylcarnitine or propionylcarnitine/free carnitine ratios were analyzed for methylmalonic acid, methylcitric acid and homocysteine by high performance liquid chromatography-tandem mass spectrometry (second-tier test).

Results 15 of 239 samples were positive by second-tier test. Among them, 13 cases were suspected methylmalonic acidurias with significantly elevated methylmalonic acid, methylcitric acid and homocysteine and 2 cases were suspected propionic acidemia with significantly elevated methylcitric acid. of 15 suspected positive cases, there were 8 true positives: methylmalonic acidurias (*n* = 6) and propionic acidemia (*n* = 2). Compared with primary screening for methylmalonic acidemia/propionic acidemia, the positive predictive value of combined primary screening and second-tier test increased from 3.3% to 53.3%, the specificity increased from 99.84% to 99.99% and the false positive rate decreased from 1.5‰ to 0.48‱.

Conclusion The second-tier test effectively improved the newborn screening performance for methylmalonic acidemia/propionic acidemia and has important clinical significance in newborn screening by tandem mass spectrometry in Zhejiang province, China.

**Keywords:** tandem mass spectrometry; newborn screening; second-tier test; screening performance

### P52. Routine Newborn Screening for MMA—What Are the Implications?

KumarKishoreNagarNandiniS.V.GirishCloudnine Hospitals, India, Neonatology, Bangalore, India

Objectives and Study Routine newborn screening program for metabolic disorders like Methyl Malonic Acidemia (MMA)—is it worth the effort and money in India, though it is an established practice in many parts of the world.

Methods Subjects and interventions

Newborn screening for metabolic disorders was performed on samples obtained from various hospitals in South India between 19th January 2007 and 19th September 2017. Dried blood samples from either heel prick or vene-puncture were collected @ 36 hours of age from babies, on special filter papers, dried & sent in for analysis by Tandem Mass Spectrophotometry (TMS).

Results In the study period, a total of 54,426 samples were obtained. From these samples received from various hospitals, abnormal results were detected in 221 (0.41%). These samples had mild elevation in both propionyl-carnitine (Normal range being 0.3-4.2 µmol/L whole blood) and methylmalonic acid (Normal levels < 1.10) that at levels often associated with a maternal vitamin B12 deficiency but not indicative of MMA. Hence all these abnormal samples parents were counselled, maternal vitamin B12 levels checked and given injection of Vitamin B12 and Newborn Screening test was repeated after 3 weeks in the babies—and all the 221 samples were NORMAL when this analysis was repeated on these babies.

Conclusions

- The incidence of most of the inborn errors of metabolism is unknown in India.
- Incidence of MMA was “thought to be higher” in the past but no accurate estimations are available.
- In our study the incidence of “Pseudo MMA” was shown to be nearly 0.5% which hitherto could have been “interpreted as MMA” accounting for the belief that MMA is higher in incidence in India.
- Untreated and undetected these “pseudo MMA” can manifest themselves as various disorders in infancy including “Infantile Tremor Syndrome”

**Keywords:** newborn screening; methyl malonic acidemia (MMA)

### P53. Clinical Application of LC-MS/MS in the Follow-up for Treatment of Children with Methylmalonic Aciduria

WangYanyunNanjing Maternity and Child Health Care Hospital, Center for Genetic Medicine, Nanjing, China

Objective: To explore the value of high performance liquid chromatography-tandem mass spectrometry (LC-MS/MS) in the follow-up for treatment of children with Methylmalonic Aciduria (MMA).

Methods: Methylmalonic acid (MMA), 2-methylcitric acid (MCA) and homocysteine (Hcy) were detected by LC-MS/MS in a total of 1,016 samples whose estimated 0.5th and 99.5th percentiles was taken as the reference value. The samples of children with MMA and propionic aciduria (PA) who were followed up in our hospital from January 2017 to August 2018 were collected. Samples of dried blood spots, serum and urine were taken from each patient on the same day. The concentration of C3 indicator in dried blood spots was tested by MS/MS. MMA, MCA and Hcy in dried blood spots were quantitatively determined by LC-MS/MS, the concentrations of MMA and MCA in urine filter papers were determined by gas chromatography-mass spectrometry (GC/MS) and the concentration of homocysteine in serum was determined by enzymatic cycling assay.

Results: Reference value of MMA, MCA and HCY by LC-MS/MS in newborn population were determined. The samples from a total of 50 patients were collected, 48 samples were from children with MMA and 2 samples were from children with PA. The first-order equation regression coefficient of MMA in blood spots and MMA in urine was significant (*p* < 0.05), r^2^= 0.736; the first-order equation regression coefficient of MCA in blood spots and MCA in urine was significant (*p* < 0.05), r^2^ = 0.946; the first-order equation regression coefficient of tHcy in blood spots and Hcy in serum was significant (*p* < 0.05), r^2^ = 0.771.

Conclusion: LC-MS/MS can be used for the follow-up of children with MMA after treatment but it is necessary to establish a reference interval suitable for the local population.

F. Tandem Mass Spectrometry Various

### P54. High Resolution Melting Analysis as Second-Tier Molecular Test For Citrin Deficiency in Newborn Screening

ChenPinwenHsuLiwenChiangShuchuanWangShiaofangChenLihsinChienYinhsiuNational Taiwan University Hospital, Department of Medical Genetics, Taipei, Taiwan

Introduction: Tandem mass spectrometry (MS/MS) analysis is a powerful tool for newborn screening and many inherited metabolic diseases are currently screened using MS/MS. However, the sensitivity of MS/MS screening for inborn errors of metabolism differ among individual diseases and false results can occur due to the nature of the specific diseases. Neonatal intrahepatic cholestasis caused by citrin deficiency (NICCD) can be detected by abnormal newborn MS/MS results but sensitivity is not satisfactory. Therefore, additional second-tier testing may be required to improve screening for citrin deficiency.

Materials and Methods: The mutations for the SLC25A13 gene of citrin deficiency, were analyzed in newborns who demonstrated an inconclusive primary screening result. The extracted DNA from newborn screening samples were scanned for hotspot mutations (c.851del4, c.1638ins23, IVS6+5G>A, IVS16ins3kb, c.1399C>T, c.955C>T, IVS11+1 G>A, c.754G>A, c.1092_1095delT, c.1063C>G, c.1064G>A and c.1231G>A) by high resolution melting (HRM) analysis. The neonates with elevated level of citrulline, methionine or total galactose and mutation of *SLC25A13* gene were referred for further confirmation tests.

Results: The results revealed that 404 of 32,768 neonates received a second-tier test for citrin deficiency and one patient was identified with c.851del4/IVS6+5 G>A compound heterozygous mutation, 10 carriers with mutants c.851del4 (*n* = 4), IVS 6+5G>A (*n* = 3), IVS16ins3kb (*n* = 1), c.1156G>A (*n* = 1) and c.1658G>A (*n* = 1). The frequency of carriers was about 1/34.

Conclusions: HRM analysis is a simple, rapid and robust method for detecting SLC25A13 mutations. Utilizing a molecular second-tier test for citrin deficiency is feasible and can improve newborn screening tests sensitivity.

### P55. Research of a Family with a Heterozygous Mutation of HPD (c.460G>A)

JiaChenluLiuSunaTianYuanZhangLinlinJiaLiting*Third Affiliated Hospital of Zhengzhou University, Screening Center, Zhengzhou 450052, China*Correspondence: jialt@163.com

Background HPD recessive mutations were often recognized as causative for one of Tyrosinemia, type III. Recently, HPD dominant mutations were also recognized as causative for Hawkins Nuria.

Objective We report the clinical and laboratory findings about a family with a heterozygous mission of HPD (c.460G>A) which owns Hawkinsinuria.

Methods We sequence series of genes involved in Tyrosinemia. through Chip capture high-throughput sequencing. Then we validate these genes through Sanger sequencing.

Results We find that the proband has a HPD gene c.460G>A heterozygous mutation. The proband’father has aHPD gene c.460G>A heterozygous mutation and owns Hawkinsinuria.

Conclusion This study provides further evidence for AD Hawkinsinuria associated with HPD c.460G>A heterozygous mutation.

### P56. Screening of MCAD Deficiency in Japan:18 Years’ Experience of Enzymatic and Genetic Evaluation

HaraKeiichiOkadaSatoshiAisakiJunkoTajimaGoNational Hospital Organization Kure Medical Center, Pediatrics, Kure, Japan

Introduction Medium-chain acyl-CoA dehydrogenase (MCAD) deficiency is one of the most representative disorders of fatty acid oxidation (FAOD). In Japan, pilot study of tandem mass spectrometry-based screening started in 1997. Since the first patient was found in 2000, the number of patients gradually increased and finally the official nationwide newborn screening realized in 2014. Correlation between genotype and clinical and biochemical phenotype of Japanese patient has not been fully clarified.

Methods Newborns and symptomatic patients showing elevation of C8-acylcarnitine in blood were evaluated by measurement of MCAD activities in lymphocytes and direct sequencing of ACADM gene.

Results We diagnosed MCAD deficiency in 11 out of 22 symptomatic patients, 60 out of 77 newborns and 6 asymptomatic siblings of affected patients. Residual activities of symptomatic patients were lower than 3% of the average of normal control values, except for a patient with 10% activity. Concerning affected newborns and sibling, 32 of the 66 patients were left with activities around 10% or lower and 28 with activities ranging from 13 to 38%. The other 6 showed higher activities (43–93%) associated with persistent elevation of C8-acylcarnitine in serum and biallelic variants were detected. The most prevalent mutation was c.449-452delCTGA (p.T150Rfs), detected in 29 alleles of 25 patients, followed by p.R17H, p.G362E, p.R53C and p.R281S. These five variants accounted for approximately 50% of all the alleles examined. Caucasian common variant c.985A>G (p.K329E) was not detected at all.

Discussion As the K329E mutant MCAD has been reported to retain 10% to 20% of activity, approximately two thirds of our asymptomatic patients are associated with actual risk of acute clinical onset, while the rest are not likely to present any symptom. Further accumulation of data of enzymatic and genetic evaluation is essential to enable appropriate medical management of Japanese patients with MCAD deficiency.

### P57. Pilot Study of Expanded Newborn Screening for 573 Genes Related to Severe Diseases in China: Results from 1173 Newborns

LuoXiaomeiZhangHuiwenYuYongguoGuXuefanXin Hua Hospital Affiliated to Shanghai Jiao Tong University School of Medicine, Endocrine and Genetic Department, Shanghai, China

Background: Current newborn screening (NBS) in China mainly aims to detect biochemical level of specific metabolites in blood or urine, which may be fluctuated along time and generates false positive/negative results.

Methods: We performed a pilot study to expand current NBS through next generation sequencing (NGS) using dried blood spots (DBS). 573 genes related to severe diseases were selected in this panel, a total of 1173 newborns were random enrolled in Shanghai that were born in September 2016. The newborns had been screened for Phe, TSH, 17-OHP and G6PD as traditional biochemical NBS previously. We then investigated the carrier frequencies of 77 genes causing inherited metabolic disorders (IMDs) in these newborns.

Results: DNA extracted from 1127 DBS samples (96.1%) was qualified for NGS. Biochemical results showed that 9 newborns were positive, including 5 newborns (1 male, 4 females) with high level of 17-OHP and four newborns (all males) with low level of G6PD. Genetic results showed that congenital adrenocortical hyperplasia (CAH) related gene variants were not detected in 5 newborns with high level of 17-OHP, while 4 male newborns with low level of G6PD all carried G6PD hemizygous variants. Besides, there were 3 more newborns with two variants of MYO1A, DUOX2 and SLC22A5 respectively. Follow-up in April 2019 illustrated that newborns with positive 17-OHP values were all healthy except for one out of touch. The top five carrier frequencies in these newborns were PAH (2.04%), ETFDH (1.24%), MMACHC (1.15%), SLC25A13 (0.98%) and GCDH (0.80%).

Discussion: Our study provides data that combined biochemical results with genetics variants of newborns, which can be helpful for evaluation of other expanded NBS strategies. This is the first study that reveals carrier frequencies of 77 genes causing IMDs related to amino acid metabolism, organic acid metabolism and fatty acid oxidation in China.

### P58. Retrospective Analysis of 5 Cases of Hypermethioninemia

ZhangZhileiSunYunWangYanyunMaDingyuanChengWeiJiangTaoNanjing Maternity and Child Health Care Hospital, genetic center, Nanjing, China

Hypermethioninemia is a group of diseases with elevated plasma methionine caused by hereditary and non-hereditary factors. Among them, the disease caused by methionine adenosyltransferase I/III deficiency is the most common and its characteristic is persistent and isolated hypermethioninemia. S-adenosylmethionine is the product of methionine, which can be used as a direct methyl donor of many substances, such as choline and nucleotide and essential in the development of body. Among the patients, most have no symptoms and a small number have central nervous system complications with high level plasma methionine, including mental retardation, cognitive impairment and special breathing odor. Methionine adenosyltransferase I/III deficiency is a common reason of Met elevation in neonatal screening by tandem mass spectrometry. And it is a non-benign disease which need to long-term follow-up. In this study, 5 cases of methionine adenosyltransferase I/III deficiency were diagnosed and retrospectively analyzed among 220,000 newborns.

**Keywords:** methionine adenosyltransferase I/III deficiency; hypermethioninemia; MAT1A; neonatal screening

### P59. Description of the Molecular and Phenotypic Spectrum of Aromatic l-Amino Acid Decarboxylase Deficiency in Thirteen Chinese Mainland Patients

DaiWeiqianYuYongguoShanghai Xinhua hospital, Endocrine inheritance, Shanghai, China

Aims: The purpose of this study is not only broadening the spectrum of diseases phenotype but also providing valuable data to establish genotype-enzymatic-clinical phenotype correlation.

Patients and Methods: We recruited 13 previously undescribed patients, who received a diagnosis of AADC deficiency from 2017 to 2019 in Chinese Mainland. The sequence status of the DDC gene was available for all 13 patients, which detected using whole exome sequencing (WES) and verified the results by Sanger sequencing. Moreover, we make use of PyMol software to inspect all identified mutation enzyme residues, in our study, position and possible contact. In addition, we “In Vitro” expression systems have been carried out to determine the impact of two compound heterozygous mutations on the structural and/or functional features.

Results: Clinical manifestation of most of our patients was consistent with the previous descriptions from the literature but some special phenotype was firstly described in our study. Moreover, most of all eleven different mutations were identified in the DDC gene, including six novel mutations and five previously reported mutation. Ultimately, functional and conformational impact of amino acid replacement on 3D networks of side-chain interactions have been observed and we further analysis catalytic features of the variants.

Conclusion: Perhaps the most intriguing aspect of our study was providing valuable data to establish genotype-enzymatic-clinical phenotype correlation in Chinese Mainland. Moreover, in this study, clinical manifestations, accessory examination, as well as therapeutic response of our subjects expand the clinical, diagnose and treatment spectrum of AADC deficiency. In consideration of AADCD is surprisingly difficult to catch in Chinese Mainland, the data from our study are critical to guide the development of treatment strategies for the disease in the further.

### P60. Mitochondrial 3-Hydroxy-3-Methylglutaryl-CoA Synthase Deficiency: A Patient with Severe Metabolic Acidosis Carrying Two Novel Mutations

LiuHaoMiaoJingkunYuChaowenWanKexingZhangJuanYuanZhaojianYangJingZouLinWangDongjuanChildren’s Hospital of Chongqing Medical University, Center for Clinical Molecular Medicine, Chongqing, China

Mitochondrial 3-hydroxy-3-methylglutaryl-CoA synthase (mHS) deficiency is an autosomal recessive inborn error of metabolism, which will give rise to failure of ketogenesis in liver during illness or fasting. It is a very rare disease with fewer than 20 patients reported worldwide. Here we reported a 9-month-old boy with mHS deficiency presenting severe and persistent metabolic acidosis after diarrhea and reduced oral food intake. These symptoms persisted even after supplementation with sugar and alkaline solution. Blood purification and assisted respiration alleviated symptoms but a second onset induced by respiratory infection several days later led to multiple organ failure and death. The urine organic acid analysis during acute episode revealed an unusual pattern of ketogenic dicarboxylic and 3-hydroxy-dicarboxylic aciduria with notable elevation of 3-hydroxybutyric acid, glutaric acid and adipic acid which may give a clue to mHS deficiency meanwhile the presence of ketone cannot exclude the disease. Analysis of plasma acyl-carnitines revealed elevated 3-hydroxybutyrylcarnitine (C4-OH) and acetylcarnitine (C2). The elevation of C2 was also observed in previous cases due to supplement of L-carnitine, however the elevation of C4-OH was reported for the first time with the possible origin of 3-hydroxybutyric acid. The results of whole exome sequencing revealed two novel compound heterozygotic mutation in HMGCS2 (c.100C>T and c.1465delA). Our patient highlights a severe phenotype of mHS deficiency with an unusual urine organic acid spectrum. Persistent acidosis was the most conspicuous problem which was difficult to correct unless blood purification was performed in this case. Recurrent illness should be avoided because it could induce severe metabolic crisis, possibly leading to death.

**Keywords:** mitochondrial 3-hydroxy-3-methylglutaryl-CoA synthase; hypoglycemia; ketogenesis; inherited metabolic disorders

### P61. A Girl with Maternal 3-Methylcrotonyl-Glycinuria Detected by Newborn Screening

HeRuxuanKangLuluLiuYiJinYingLiMengqiuYangYanlingPeking University First Hospital, Department of Pediatrics, Beijing, China

Objective: 3-methylcrotonyl coenzyme A carboxylase (MCCC) deficiency is an autosomal recessive organic aciduria caused by leucine metabolism disorder, which is included in the newborn screening programs using tandem mass spectrometry. We present a case of maternal 3-methylcrotonylglycinuria.

Methods: A newborn, a one-month old girl, with high blood 3-hydroxy-isovalerylcarnitine (C5OH) was referred to us. For the differential diagnosis, the urine organic acids and blood amino acids and acylcarnitines of the baby and her mother were analyzed. Genetic analysis by whole exome sequencing was also performed for the family to confirm the diagnosis.

Results: Both of the baby and her mother were asymptomatic. The baby had mildly elevated blood C5OH and normal urine organic profile. But her mother had significantly increased blood C5OH and urine 3-methylcrotonoyl-glycine. A novel heterozygous mutation, c.1144_1147inv (p.K382_M563delinsF) was identified in the MCCC2 gene of the baby. But a homozygous mutation c.1144_1147inv on MCCC2 gene was found in her mother. The novel mutation, c.1144_1147inv in the MCCC2 gene was predicted to impair the protein stability. Therefore, the mother was diagnosed as 3-methylcrotonyl-CoA carboxylase deficiency. The baby was a heterozygote with maternal 3-methylcrotonyl-glycinuria. She is one year old now with normal psychomotor development.

Conclusions: For the newborn with increased blood C5-OH, maternal 3-methylcrotonyl-CoA carboxylase deficiency should be considered as a differential diagnosis. Blood amino acids and acylcarnitines, urinary organic acids in the mother should be checked.

### P62. Detection of Methylmalonic Acid, Methylcitric Acid and Total Homocysteine in Dried Blood Spots Using a Modified LC-MS/MS Method

LiuYiKangLuluWangJunjuanJinYingLiMengqiuSongJinqingLiHaixiaYangYanlingPeking University First Hospital, Department of Pediatrics, Beijing, China

Objectives Blood propionylcarnitine (C3) and methionine (Met) are less specific markers for methylmalonic acidemia, propionic acidemia and homocysteinemia. Urine organic acids analysis and gene test should be performed for differential diagnosis. In this study, we used a modified liquid chromatography-tandem mass spectrometry (LC-MS/MS) method to measure methylmalonic acid (MMA), methylcitric acid (MCA) and total homocysteine (tHcy) in dried blood spots and evaluated its ability to rapidly identify the above targeted diseases.

Methods Subjects included 140 healthy controls and 228 patients of inherited metabolic disorders with definite genetic diagnosis (205 cases with methylmalonic acidemia, 17 cases with propionic acidemia, 2 cases with homocysteinemia type 1 and 4 cases with homocysteinemia type 2 or type 3). Blood amino acids and acylcarnitines were determined by current LC-MS/MS assay. Blood MMA, MCA and tHcy were determined in the same dried blood spots by a modified LC-MS/MS method. Serum tHcy was measured by immunoassay.

Results The blood MMA, MCA and tHcy concentrations of 140 controls were 0-1.46 μmol/L, 0.01–0.42 μmol/L and 0.73–9.15 μmol/L, respectively. Blood C3 and MMA were elevated in 83.9% and 99.5% of the 205 patients with methylmalonic acidemia. Blood C3 and MCA were obviously increased in all the patients with propionic acidemia. Elevated blood tHcy were found in all the 6 patients with homocysteinemia, while Met were increased in only 2 patients with homocysteinemia type 1. Blood MMA and MCA correlated with blood C3, respectively. And blood tHcy level also correlated with serum tHcy.

Conclusions The normal ranges of blood MMA, MCA and tHcy in Chinese children were first provided. Supplementary determination of MMA, MCA and tHcy in dried blood spots using LC-MS/MS method might have a good potential in the early diagnosis and treatment monitoring for methylmalonic acidemia, propionic acidemia and homocysteinemia.

### P63. Clinical Manifestations of Patients with Glutaric Acidemia Type I and the Gene Mutation Analysis

HanLianshuEHuishuXinhua Hospital, Shanghai Jiaotong University School of Medicine, Department of Pediatric Endocrinology and Genetic, Shanghai Institute for Pediat, Shanghai, China

Objective: Glutaric acidemia type I (GA-I) is a rare neurometabolic disorder caused by inherited deficiency of glutaryl-CoA dehydrogenase (GCDH). The aim of this study was to elucidate the clinical features from 56 Chinese GA-I patients and assess a potential correlation between genotype and phenotype.

Methods: Fifty-six patient’s clinical manifestations and MRI findings were collected. All patients were diagnosed by elevated blood glutarylcarnitine (C5DC), the ratio of C5DC to capryloylcarnitine (C8) (C5DC/C8) and increased urinary glutaric acid (GA). GCDH genes were analyzed by polymerase chain reaction (PCR) and direct sequencing. The novel missense mutations were assessed by bioinformatic analysis and screened against alleles sequenced from 100 control participants.

Results: Macrocephaly, developmental delay and convulsion were the most common manifestations of GA-I. The median levels of blood C5DC, C5DC/C8 ratio and the urinary GA were 0.91µmol/L (0.21–5.56), 25.11 (4.06–109.78) and 899.88 mmol/molCr (237.68–4514.2), respectively. White matter changes, wide cerebrospinal fluid spaces anterior to the temporal lobes and cerebral atrophy were rather typical in MRI images. A total of 53 different GCDH mutations were found, while c.1244-2A>C being the most common. 12 novel mutations included c.91G>T, c.109_110delCA, c.352delG, c.364G>C, c.452C>T, c.493C>A, c.632_639delCCAGGATC, c.647C>T, c.767T>C, c.784G>A, c.1060G>T and c.1133C>T.

Conclusion: A total of 53 GCDH mutations were detected in the Chinese population, including 12 novel ones. c.1244-2A>C was the most frequent mutation while a genotype-phenotype correlation could not be found.

### P64. A Novel Electron Transfer Flavoprotein Dehydrogenase (ETFDH) Gene Mutation Identified in a Newborn with Glutaric Acidemia Type II: A Case Report of a Chinese Family

OuMingcaiZhuLinZhangYongHuQiZhangYaguoZhouJingyaoZhangYuChenXuelianYangLijuanLiTingSuXingyueWangWenjunSichuan Provincial Hospital for Women and Children, Department of Neonatal Disease Screening, Chengdu, Sichuan Province, China

Background: Glutaric acidemia type II (GA II) or multiple acyl-CoA dehydrogenase deficiency (MADD, OMIM 231680) is an inherited autosomal recessive disease affecting fatty acid, amino acid and choline metabolism, due to mutations in one of three genes namely, electron transfer flavoprotein alpha-subunit, ETFA, electron transfer flavoprotein β-subunit, ETFB and electron transfer flavoprotein dehydrogenase, ETFDH. Currently, few studies have reported genetic profiling of neonatal-onset GA II. This study aimed to identify the genetic mutations in a Chinese family with GA II.

Case presentation: We reported a case of GA II with purulent meningitis and septicemia and identified a novel ETFDH gene mutation in a female infant. The patient developed an episode of hypoglycemia and hypotonicity on the postnatal first day. Laboratory investigations revealed elevations of multiple acyl carnitines indicating glutaric acidemia type II in newborn screening analysis. Urinary organic acids were evaluated for the confirmation and revealed a high glutaric acid excretion. Genetic analysis revealed two mutations in the ETFDH gene (c.623_626 del/c. 1399G>C), which were considered to be the etiology for the disease. The novel mutation c.623_626 del was identified in the proband infant and her father, her mother was carriers of the mutation c.1399G>C.

Conclusions: A novel variant (c.623_626 del) and a previously reported missense (c.1399G>C) in ETFDH gene have been identified in the family. The two variants of ETFDH gene identified probably underlie the pathogenesis of Glutaric acidemia type II in this family and also enlarge ETFDH genotype-phenotype correlations spectrum.

**Keywords:** glutaric acidemia type II; multiple acyl-CoA dehydrogenase deficiency; ETFDH; neonatal-onset

### P65. 3-O-Methyldopa Detection in Dried Blood Spot Assist Diagnosis of Aromatic l-amino Acid Decarboxylase Deficiency

LuMeiYangYanlingXiamen Maternal and Child Care Hospital, Department of Pediatrics, Xiamen, Fujian China, China

Introduction Aromatic L-amino acid decarboxylase (AADC) deficiency is a rare inborn error of neurotransmitter biosynthesis which leads to a combined deficiency of catecholamines and serotonin. It is an autosomal recessive metabolic disorder of monoamines caused by DDC gene mutations. The patients presented with global developmental delay, involuntary movements, autonomic dysfunction and high concentration of 3-O-Methyldopa.

Case Report A 1-year-old girl was admitted to PICU because of the episode of sudden cardiac arrest and pneumonia. Oculogyric crises and bradycardia (heart rate 40-70 bpm) without hypotension were noticed. The bradycardia showed poor response to intravenous dopamine.

She was normal before the age of 3 months. When she was 3-month-old, malnutrition, cerebral palsy, hypotonia, hypersalivation, dystonia, feeding dysfunction and progressive developmental delay were observed.

Her brain MRI and EEG was normal. Blood amino acids and acylcarnitine profiles, ammonia, lactate and urine amino acids were normal. Neurotransmitter disorders were suspected.

Compound heterozygous mutations, c.179T>C (p.V60A, from her father) and c.174+4A>C (from her mother) in her DDC gene were identified.

3-*O*-Methyldopa in her dried blood spot increased significantly (635 nmol/L vs normal control 0-300 nmol/L). Aromatic L-amino acid decarboxylase deficiency was confirmed.

Conclusions In the patients with hypersalivation, oculogyric crises, sudden cardiac arrest and bradycardia, aromatic L-amino acid decarboxylase deficiency should be awareness. 3-*O*-Methyldopa detection in dried blood spot assist the diagnosis of aromatic L-amino acid decarboxylase deficiency.

### P66. Application of Region 4 Stork Project in Suspected Very Long Chain acyl-CoA Dehydrogenase Deficiency from Neonatal Screening Detected by Tandem Mass Spectrometry

YangJianbinZhangChaoHuZhenzhenHuangXinwenYangRulaiShangShiqiangWuDingwenMaoHuaqingHuLingweiZhaoZhengyanThe Children’s Hospital Zhejiang University School of Medicine, Neonatal Screening Center of Zhejiang Province, Zhejiang Key Laboratory for Diagnosis and Therapy of Neonatal Diseases, Ministry of Education Key Laboratory of Reproductive Genetics, Genetics and Metabolism, Hangzhou, China

The elevated levels of blood C14:1-acylcarnitine and related ratios detected by tandem mass spectrometry (MS/MS) are recognized as the characteristic biomarker for very long chain acyl-CoA dehydrogenase deficiency (VLCADD). However, MS/MS has led to the large increases of false positive results. Region 4 Stork (R4S) collaborative project is defined evidence-based cutoff target ranges for all analytes detected by MS/MS and related ratios, then designed the postanalytical interpretive tools to convert metabolic profiles into a composite score driven by the degree of overlap between normal population and disease range. To investigate the feasibility of R4S project in suspected VLCADD from neonatal screening detected by MS/MS, the retrospective study was performed among 2,040,072 neonates screened by MS/MS in Neonatal Screening Center of Zhejiang Province, China from October 2013 to July 2018. There were 910 neonates suspected VLCADD by cutoff system of tandem mass spectrometry. Raw data of these screened neonates were uploaded into R4S website to perform postanalytical interpretive tools, then compared with the results of cutoff system. The rate of positive predictive value (PPV), specificity and false positive rate (FPR) were calculated by cutoff system of MS/MS and R4S project, respectively. Compared with cutoff system by using R4S project, the neonates suspected VLCADD were reduced from 910 to 238, 672 false positive cases were excluded and 9 were diagnosed as true positive cases by confirming genetic analysis. The PPV increased from 0.99% to 3.78%, the specificity increased from 99.96% to 99.99% and the FPR declined from 0.044% to 0.011%. Region 4 Stork postanalytical tools are efficient, high-throughput and can achieve notable improvements of PPV and FPR in suspected VLCADD from neonatal screening detected by tandem mass spectrometry.

**Keywords:** very long chain acyl-CoA dehydrogenase deficiency; Region 4 Stork project; neonatal screening; tandem mass spectrometry; false positive rate

### P67. A Study for Newborn Screening and Genotype Analysis of Hereditary Tyrosinemia

TongFanThe Children’s Hospital, Zhejiang University School of Medicine, Department of Genetic and Metabolism, Hang Zhou, China

Objective To investigate the newborn screening and epidemiological characteristics, genotype, clinical features of hereditary tyrosinemia (HT) in southern Chinese population.

Methods: A retrospective analysis was conducted from November 2013 to November 2018 in the newborn screening center.

Results: Based on 2,188,784 neonatal screening by MS-MS, three HT cases were diagnosed with an incidence of 1 in 729,594, including 1 tyrosinemia type I (HT1)case (homozygous variations of c.455G>A in *FAH* gene), 1 tyrosinemia type II(HT2) case (heterozygous variations of c.890G>T and c.408+1G>A in *TAT* gene), 1 tyrosinemia type Ⅲ(HT3) case (homozygous variations of c.257T>C in *HPD* gene), respectively. The positive predictive value of HT in newborn screening was 3.4%.The case of HT1,with 666.9 µmol/L of screening TYR and 3.87 µmol/L of SA values, appeared with cholestasis, mild elevated of liver enzyme and lactic acid and died at 2 months old even with medical milk powder (tyrosine and phenylalanine free).The case of HT2, with 625.6 µmol/L of screening TYR and normal SA, has normal score of Bayley assessment with medical milk treatment. The case of HT3 is 29 months old now, with 1035.3 µmol/L of screening TYR and normal SA, the values of TYR fluctuated from 532.1 µmol/L to 1060.3 µmol/L due to irregular medical milk treatment. The score of Bayley assessment was normal.

Conclusions: HT is rare in the southern Chinese population. The novel mutations of c.890G>T, c.4081G>A of TAT and c.257T>C of HPD were found, which expended the gene spectrum of HT. The case with genotype of FAH c.455G>A homozygous appeared with acute phenotype. The prognosis of HT2 who was diagnosed by newborn screening and early treated by medical diet is good. HT1 should be treated by compound medical diet with nitisinone as soon as possible, otherwise the prognosis is poor. The HT3 prognosis is not clear so far due to little data.

### P68. ACADVL Gene Analysis of Neonatal with the Very Long Chain acyl-coA Dehydrogenase Deficiency

TongFan†The Children’s Hospital, Zhejiang University School of Medicine, The Department of genetic and metabolism, Hangzhou, China†Supported by the National Natural Science Foundation of China (81741090) and the National Key Research and Development Program of China (2017YFC1001703 and 2017YFC1001704).

Objective To study the clinical features and gene mutations of the very long chain acyl-coenzyme A dehydrogenase deficiency (VLCADD) in 9 neonatal and the relationship between the genotype and the phenotype of it.

Methods Based on the Neonatal screening data of VLCADD by tandem mass spectrometry (MS-MS) between January 2009 and June 2017 and the tetradecenoylcarnitine ± tetradecenoylcarnitine/octanoylcarnitine (C14:1 ± C14:1/C8) as a mark indexes for clinical diagnosis. Those cases with positive outcomes were further confirmed by the ACADVL gene sequencing.

Results Based on 2,129,989 neonatal screening by MS-MS, nine VLCADD cases were diagnosed with an incidence of 1 in 236,665. Except one case loss to follow-up, the observed phenotypes comprised 2 cases of severe early- onset form, 1 case of the hepatic form and 5 cases of late-onset. Good outcomes were acquired among our 6 patients except the 2 early-onset cases. A total of 16 different ACADVL mutations were detected in 9 cases, including 8 novel mutations (c.96-105del GCCCGGCCCT, c.541C>T, c.863T>G, c.878+1 G>C, c.895A>G, c.1238T>C, c.1276G>A, c.1505T>A) and 11 missense mutations. There were 9 different genotypic combinations with one homozygote and 8 compound heterozygotes. All cases, except for two which carry null mutations, have good outcome.

Conclusion The disorder of VLCADD is relatively rare in southern China, in which late-onset form is abundant. Carrying null mutation of ACADVL may related bad clinical outcome. The data presented here will provide a valuable tool for improved diagnosis and management of future cases of VLCADD in China.

**Keywords**: very long chain acyl-coenzyme A dehydrogenase; neonatal; gene mutation; phenotype; inherited metabolic disease

### P69. Detection of Vitamin B12 Deficiency by Newborn Screening for Homocystinuria

SkrinskaVictorRamaswamyMamathaMitriRolaTahtamoniMontherAlabedMamoonJoshiRaviRamaKahlilAbdohGhassanAbdeinLubnaMohammedKhadijaHamad Medical Corporation, Laboratory Medicine and Pathology, Doha, Qatar

Homocystinuria has a high prevalence rate of approximately 1:2000 in Qatar. Due to the high prevalence and the inability to detect homocystinuria effectively with a typical amino acid/acylcarnitine screen by liquid chromatography/tandem mass spectrometry (LCMSMS), all newborns in Qatar are screened for total homocysteine (HCY) on dried blood spots using a previously published rapid LCMSMS chromatographic method. Originally, a cutoff of < 14 μmol/L was used for reporting normal results. Samples with results > 14 μmol/L were reflexed to a 2nd tier LCMSMS chromatographic method for determination of methylmalonic acid (MMA). The results from the HCY, MMA and amino acid/acylcarnitine screens were analyzed for differentiation of homocystinuria and other disorders including vitamin B12 deficiency. While the 14 μmol/L cutoff was effective in identifying homocystinuria, it also detected a number of vitamin B12 deficiencies with HCY results between 14 to 20 μmol/L. To determine whether additional vitamin B12 deficiencies can be detected with a lower HCY cutoff, the cutoff was lowered to 8 μmol/L for reflexing samples to 2nd tier analysis for MMA. Newborns with results for MMA > 5 μmol/L were recalled for testing of the serum vitamin B12 level. Over a 7-month period, 11 newborns out of approximately 16,000 screens were identified with vitamin B12 levels below the detection level of 111 pmol/L and an additional 5 newborns were identified with vitamin B12 levels between 111 to 170 pmol/L. The HCY levels ranged from 10.0 to 26.4 μmol/L and the MMA levels ranged from 5.6 to 16.1 μmol/L. The levels of methionine and propionylcarnitine were normal. The nationalities of the deficient newborns were predominantly Middle Eastern. Further studies are underway to optimize an effective algorithm for detection of vitamin B12 deficiency in newborns.

### P70. Newly Identified Mutations of the IVD Gene in two Cases of Isovaleric Acidemia in Chinese populations

LiuJingWangHuaYanHuimingJiaZhengjunHunan provincial Maternal and Child Health Care Hospital, Newborns Screening Center of Hunan Province, Changsha, China

Isovaleric acidemia (IVA) is an autosomal recessive inborn error affecting leucine metabolism. It is caused by a deficiency in isovaleryl-CoA dehydrogenase (IVD), a mitochondrial matrix enzyme that catalyzes the oxidation of isovaleryl-CoA to 3-methylcrotonyl-CoA. IVD is a FAD-containing enzyme, consisting of four identical subunits. Clinical features of IVA include poor feeding, vomiting, lethargy, developmental delay, metabolic acidosis and a characteristic "sweaty foot" odor. IVA is one of the target disorders for newborn screening by tandem mass spectrometry (MS/MS). The clinical manifestations can be divided to three types: metabolically severe, chronic intermittent and asymptomatic. To date, over 90 disease-causing mutations have been reported worldwide. In this study, we searched for IVD mutations in two Chinese patients with IVA (asymptomatic type). The diagnosis of IVA was confirmed by MS/MS, blood spot acylcarnitine profiles by MS-MS demonstrated an elevation of C5-carnitine with a peak concentration of 1.08 and 2.02 micromol/L (<0.41 micromole/L). Organic acid analysis of urine by GC-MS was normal for both. No feeding problems were observed. All coding exons and the flanking introns in the IVD gene were scanned by high throughout sequencing and the mutations were amplified by Sanger sequencing. We thus identified two hitherto unknown mutations (p.T214A, p.L45R). Patient No. 1 was homozygous for c.640A>G (p.T214A) mutation, while patient No. 2 was a compound heterozygote for 2 mutations, a maternally-inherited c.134T>G (p.L45R) and a paternally inherited c.640A>G (p.T214A). According to the ACMG classification guideline, all the two mutations are classified to be uncertain mutation. Our results have illustrated the heterogeneous mutation spectrum in the Chinese patients. These two cases highlight the importance of screening for metabolic diseases including rare metabolic diseases in the early neonatal period and further expand the mutant spectrum of IVA.

### P71. Analysis of Characteristics of LC-MS/MS Blood Amino Acids and Carnitine in Premature Infants

LinCaijuanFanXinGengGuoxingLuoJingsiHuangXiaotaoLinFeiLiWeiXuYuqiMaternal and Child Health Hospital of Guangxi, Laboratory of Genetics and Metabolism, Nanning, China

Objective By using Tandem Mass Spectrometry to detect the metabolic levels of amino acids and carnitine in premature newborns and normal-term infants, according to the changes in the blood, the reference range is provided for nutritional support for premature infants.

Method 911 premature neonates (25-36 weeks) and 2582 normal-term neonatal filter papers were collected in the neonatal ward of our hospital, the samples were subjected to analyze 11 amino acids and 31 kinds of Carnitine using the Tandem Mass Spectrometry. Statistical methods were used to compare the differences between groups with Kruskal Wallis rank sum test.

Results From a gender perspective, among 911 preterm neonates (512 males and 399 females), except for C4DC+C5OH, the other carnitines were not different in the preterm group (*p* > 0.05) and 11 amino acid levels were no difference (*p* > 0.05); 911 premature infants were divided into gestational weeks of birth Except C8 and C14:2, the other carnitines were different (*p* < 0.05), among which C2, C3, C6DC, C14, C14:1, C16, C16:1, C16OH, C16:1OH, C18:1 increases but C5, C18:2 decrease, the other 10 amino acids except Phe were different (*p* < 0.05) and Cit, Val, Arg, Orn decreased but Ala increased; From the point of weight, 911 premature infants were divided into two groups Compared with the full-term infants, except for C0, C4, C4DC+C5OH, C14:2, the other carnitines were different (*p* < 0.05), C2, C3DC+C4OH, C6DC, C10, C12, C12: 1, C14, C14: 1, C14OH, C16, C16OH, C16: 1OH, C18, C18: OH, C18: 1OH increased while C4, C4DC + C5OH, C5, C5: 1, C18:2 reduced, the other 10 amino acids except Phe were found to be different (*p* < 0.05) and the Ala, Gly, Pro increased while the Met and Arg decreased.

Conclusion The levels of amino acids and carnitine in premature infants are different from those in normal term infants. Therefore, the cut-off value and reference range of each gestational age indicator can reduce false positives and help clinically guide clinicians to implement preterm birth during hospitalization.

### P72. Parallel Determination of Nitisinone Levels in Dried Blood Spot and Plasma Samples of Chilean Tyrosinemia 1 Patients by using LCMSMS. Variability Assay Comparison with Results Reported from Well Child Laboratory, Evelina Children’s Hospital, London

FuenzalidaKarenGuerreroPatricioValienteAlfTurnerCharlesAriasCarolinaCornejoVeronicaUniversidad de Chile, Instituto de Nutricion y Tecnologia de los Alimentos, INTA, Santiago, Chile

Tyrosinemia type 1(Tyr-1) is an inborn error of metabolism caused by defects in tyrosine metabolism causing an accumulation of tyrosine and toxic degradation products. Treatment of Tyr-1 patients is based on nitisinone (NTBC) administration. NTBC is an herbicide and monitoring levels in body fluids are necessary for optimal dosage.

Objective: To implement NTBC determination by LCMSMS in DBS and plasma samples and to compare NTBC levels obtained in our center with those reported by Dr. Charles Turner (London) during the follow up of Tyr-1 patients between 2018–2019.

Methods: NTBC quantification by LCMSMS was implemented by using mesotriene as internal standard. DBS samples were extracted using 100% methanol. ESI^+^ was used as ionization method and analytes were detected by MRM (transitions 330 > 218 for NTBC and 340 > 228 for mesotriene). Fifty DBS samples from Tyr-1 patients were analyzed in parallel with Well Child Laboratory and 26 plasma samples were analyzed for correlation with DBS levels.

Results: We are able to detect NTBC by LCMSMS in DBS and plasma samples from Tyr-1 patients. Our method, shows good precision, accuracy and linearity with detection limit of 0,5 nmol/L. The concentration range determined in DBS samples from Tyr-1 patients was 12,0-43,3 µmol/L and 29,7-97,6 µmol/L for plasma samples. Parallel comparison of NTBC levels in DBS samples shows values 20% higher with those analyzed in Well Child Laboratory. NTBC levels in plasma and in DBS are well correlated being 2.4 times higher in plasma.

Conclusion: We validated in our laboratory the LCMSMS method used in the Laboratorio di Patologia Metabolica at Ospedale Bambino Gesu for the detection of NTBC in DBS and Plasma samples. NTBC determination of Chilean Tyr-1 patients in follow-up during 2018-2019 showed reproducible results, comparable with those obtained in Evelina Children’s Hospital, London. Good correlation was observed in DBS and plasma samples.

### P73. Profiling of Amino Acid and Acylcarnitine in Newborn Screening Dried Blood of 51 Cases with Neonatal Intrahepatic Cholestasis Caused by Citrin Deficiency (NICCD)

TangChengfangLiuSichiFengYiMeiHuifenLiuHaipingFengJinwenYeLixingWangGuoqingLiuLiHuangYonglanGuangzhou Women and Children’s Medical Center, Guangzhou Newborn Screening Center, Guangzhou, China

Objective To investigate the profiles of blood amino acid and acylcarnitine in neonates with neonatal intrahepatic cholestasis caused by citrin deficiency (NICCD) and to explore the sensitivity in newborn screening for NICCD.

Method Amino acid and acylcarnitine profiles in dried blood spots of newborn screening program were analyzed by tandem mass spectrometry (MS/MS) in 124,250 neonates born in Guangzhou from January 1, 2015 to December 31, 2018 and 50 cases with NICCD confirmed by *SLC25A13* gene analysis at 0-5 months old. Amino acid and acylcarnitine profiles and blood spots collecting time of the true positive (TP) and false negative (FN) from NICCD patients were compared.

Results Among 124,250 neonates, 31 neonates were positive for NICCD screening. One of them was confirmed NICCD and 3 cases with NICCD were found to be false negative. The positive predictive value was 0.03 and the sensitivity was 0.25. Among 51 patients with NICCD, 14 cases (14/51, 27.5%) were TP and 37 cases (37/51, 72.5%) were FN based on the cutoff value of citrulline in newborn screening dried blood spots. Compared with FN group and normal control, the TP group had significantly higher levels of blood citrulline, methionine, phenylalanine, threonine, valine, myristyl carnitine (C14:2) and octadecaenoyl carnitine (C18:2) and significantly lower levels of octadecenoylcarnitine (C18:1) and Ala/Cit ratio (*p* < 0.01). The FN group had the same trend as TP group compared to normal control. The blood sampling time of the TP group was 5 ± 2 d, which was significantly longer than the FN group (3 ± 1 d) (*p* < 0.01).

Conclusion The characteristics of blood amino acid profiles of NICCD cases in early neonatal period presented increased citrulline and multiple amino acid and decreased ratio Ala/Cit. The sensitivity and positive predictive value of neonatal NICCD screening were limited. The low sensitivity may be related to blood spots collecting early.

**Keywords:** citrin deficiency; SLC25A13 gene; dried blood spots; newborn screening

### P74. Two Novel Mutations in the BCKDHB Gene that Cause Maple Syrup Urine Disease

HanBingjuanJinan Maternal and Child Care Hospital, Newborn Screening Center, Jinan, Shandong province, China

Background: Maple syrup urine disease (MSUD) is a rare metabolic disorder of autosomal recessive inheritance caused by decreased activity of branched-chain α-ketoacid dehydrogenase complex (BCKD). Mutations in the three genes (BCKDHA, BCKDHB and DBT) are associated with MSUD. Here, we described the presenting symptoms, clinical course and gene mutation analysis of a Chinese boy with MSUD.

Methods: Plasma amino acid analysis was performed by tandem mass spectrometry and the levels of organic acids in urine were measured with gas chromatography-mass spectrometry. The BCKDHB gene was sequenced by Sanger method. Furthermore, the significance of the novel mutations was predicted by Polyphen and Mutationtaster. After diagnosed, the patient was fed with protein restricted diet to reduce intake of BCAA and was treated with L-carnitine. Metabolic parameters, clinical presentation and mental development were followed up.

Results: The patient was diagnosed as MSUD. Two novel BCKDHB mutations (c.523T>C and c.478-25_552del100) were identified. In silico analysis predicted that the two mutations were “disease causing.” The boy tolerated the treatment well and had symptomatic improvement. He presented with mild hypotonia and had nearly normal DQ scores at the age of 10 months. The two novel mutations result in the clinical manifestations of MSUD. Our results may reflect the heterogeneity of the pathogenic variants found in patients with MSUD.

**Keywords:** BCAA; BCKD; BCKDHB; gene mutation; maple syrup urine disease

### P75. Clinical and Gene Mutation Characteristics of Primary Carnitine Deficiency Found by Neonatal Screening

LiXiaoleThe Third Affiliated Hospital of Zhengzhou University, Newborn screening Center of Henan Province, Zhengzhou, China

Objective: To study the prevalence, clinical and gene mutation characteristics of primary carnitine deficiency and to provide a theoretical basis for early diagnosis and treatment, genetic counseling and prenatal diagnosis of PCD.

Methods: From January 2013 to December 2017,720667 newborns and suspected mothers were investigated for PCD by tandem mass spectrometry. An analysis of the carnitine transporter SLC22A5 gene mutation characteristics of patients diagnosed as PCD in Newborn screening Center of Henan Province, the Third Affiliated Hospital of Zhengzhou University. Dietary guidance and supplementation of l-carnitine were conducted, and growth and intelligence development were observed during follow-up among the PCD patient.

Results: 21 neonates and 6 mothers were confirmed as cases of PCD. The incidence rate of newborns was 1:34317. A total of 18 mutations were detected in the SLC22A5 gene, which included 4 previously unreported mutations: c.1484T>C, c.394-1G>T, c.431T>C and c.265-266insGGCTCGCCACC.

18 patients were carrying compound heterozygous mutations and 3 patients were carrying homozygous mutations; In the 5 confirmed mothers, 3 patients were carrying compound complex heterozygous mutations and 2 patients were carrying homozygous mutations. The prevalent mutations were c.1400C>G, c.760C>T and c.51C>G were 42.3% (22/52), 11.5% (6/52) and 7.7% (4/52). After 8-42 months follow-up, PCD in newborn screening program had no clinical symptoms and normal growth. Blood free carnitine level was raised in all mothers after treatment.

Conclusion: the incidence of neonatal primary carnitine deficiency is 1:34317. The most common mutation of PCD is c.1400C>G in Henan and the identification of new mutations enriches the SLC22A5 gene mutation profile.

**Keywords:** primary carnitine deficiency; newborn screening; SLC22A5 gene

### P76. Study of Screening, Diagnosis, Treatment and Genotype of Primary Carnitine Deficiency

YangRulaiTongFanZhengJingWuDingwenZhouYingMaoHuaqingThe Children’s Hospital Zhejiang University School of Medicine, Department of genetics and metabolism, Hangzhou, China

Objective To investigate the clinical diagnose, treatment and genetic features of infant and maternal primary carnitine deficiency (PCD) in newborn screening.

Method Blood samples of newborns were collected for the genetic metabolic diseases screening by tandem mass spectrometry in Zhejiang province. The infants with lower C0(cut-off value = 10 mmol/L) and their mothers were recalled for diagnosis.

Result A total of 4459 infants were detected with lower C0 and finally 121 subjects were diagnosed with PCD in 3,040,815 newborns screening program. The prevalence rate was 1/25,131 and the positive expected value was 2.71%. The complete follow-up data of 111 infant PCD showed that the initial screening time was 3.99 ± 1.81 days, the diagnosis time was 27.02 ± 9.23 days, the initial screening C0 was 5.94 ± 1.89 mmol/L, the recalled C0 was 4.99 ± 1.93 mmol/L. With the treatment of L-carnitine, the C0 level at maintenance dose was 24.94 ± 10.26 mmol/L, which was significantly higher than that of pre-treatment (t = 4.51, *p* < 0.01). Furthermore, 64 maternal PCD patients were identified with a prevalence of 1/47,513 and an average C0 of 3.31 ± 1.79 mol/L. The average C0 in initial screening of their infants was 5.24 ± 1.96 mmol/L; the average C0 in recall was 28.72 ± 15.54 mmol/L. The most common mutation was c.1400C>G (p.S467C) in the infant cases with PCD, accounting for 39.05%; and followed by c.51C>G (p.F17L) mutation. Among maternal cases with PCD, the SLC22A5 c.1400C>G (p.S467C) mutation accounted for 36.67%. Except for 2 deaths due to unknown reasons, other PCD patients were avoided starvation and given long-term standardized treatment with l-carnitine.

Conclusion PCD can be detected early by newborn screening, however maternal carnitine deficiency should be excluded. SLC22A5 c.1400C>G (p.S467C) is the most common mutation identified in PCD patients in our cohort. The treatment of L-carnitine is effective.

### P77. Genetic analyses of Primary Carnitine Deficiency in Guangxi China with two novel mutations

LuoJinsiLiMengtingYiShengGengGuoxingFanXinGuangxi Maternal and Child Health Hospital, Department of Genetic and Metabolic Central Laboratory, Nanning, China

Background Primary Carnitine Deficiency is a human autosomal-recessive inherited congenital myasthenic syndrome with either metabolic decompensation or cardiac and myopathic manifestations which most common presentations in infancy and early childhood. Primary systemic carnitine deficiency is caused by mutations of the SLC22A5 gene.

Objective The objective of this study was to evaluate the molecular basis of Primary Carnitine Deficiency in twenty-six Chinese patients.

Patients and Methods Peripheral venous blood samples were collected from twenty-six unrelated patients. The technology of High liquid chromatography tandem mass spectrometry (HPLC/MS/MS) was used to screen congenital genetic metabolic disease and twenty-six patients with CDSP were diagnosed. The SLC22A5 genes of all individuals were analyzed by direct DNA sequencing and the sequences compared with are reference database and bioinformatics analysis.

Results Among the 26 Primary Carnitine Deficiency patients, a total of 13 variants were detected in the 52 alleles of 26 patients with Primary Carnitine Deficiency, 2 of which were novel variants: c.516delC and c.1031C>T (p.T344I). The population screening and the bioinformatic analysis to determine the effects of the mutations, which revealed these two novel mutations were pathogenic. A correlation analysis between patient phenotype and gene variant frequency showed that c.1400C>G (p.S467C) and c.51C>G (p.F17L) may be the hotspots of SLC22A5 in Primary Carnitine Deficiency in Guangxi, China.

Conclusions In conclusion, the mutational spectrum underlying Primary Carnitine Deficiency in Guangxi China was established for the first time and the functional analysis of 2 novel SLC22A5 gene expands the mutation spectrum of oculocutaneous albinism. Correlation analysis between variants frequencies in compound heterozygous patients and phenotype severity is helpful for phenotypic prediction.

### P78. Analysis of Serial Mass Spectrometry Screening Results of Neonatal Genetic Metabolic Diseases in Guangxi

HuangXiaotaoFanXinLuoJingsiGengGuoxingLinCaijuanGuangxi Zhuang Autonomous Region Maternal and Child Health Hospital, The Center Laboratory of Genetics and Metabolism, Guangxi Neonatal Disease Scree, Nanning, China

Objective: Understand the incidence of various amino acid, fatty acid and organic acid metabolic defects in Guangxi and provide a basis for the prevention and treatment of neonatal genetic diseases.

Methods: A sample of 480,000 cases of neonatal dry blood filter papers received by our laboratory from May 2011 to December 2018 were selected for screening for genetic metabolic diseases by tandem mass spectrometry. Those with positive screening were recalled for blood collection for reexamination, urine gas chromatography-mass spectrometry, biochemical metabolites and enzymatic detection were performed when necessary and gene diagnosis was performed.

Results: of the 480,000 live births, 6,000 were initially screened positive, with a positive screening rate of 1.25%. A total of 62 cases of genetic metabolic disease were diagnosed, including 35 males and 27 females. The overall incidence rate was 1/7742, including 18 cases of amino acid metabolic diseases, the incidence rate was 1/26667 and fatty acid metabolic diseases were 33 cases. The incidence rate was 1/14545. 11 cases of organic acid metabolism, the incidence rate is 1/43,636. Among the 33 cases of fatty acid metabolic disease, 23 cases were primary carnitine deficiency, with the incidence of 1/20870.

Conclusion: Primary carnitine deficiency is the most common genetic metabolic disease among newborns in Guangxi. Combined with clinical symptoms, tandem mass spectrometry can be used in the early screening of neonatal genetic metabolic disease and guide clinical diagnosis.

**Keywords:** tandem mass spectrometry; neonatal genetic metabolic disease; incidence rate

### P79. A Simple Diagnostic Test for Carnitine-Palmitoyltransferase I Deficiency Using Tandem Mass Spectrometer

ShigematsuYosukeYuasaMioriSugiharaKeiichiHataIkueTajimaGoUniversity of Fukui, Department of Pediatrics, Fukui, Japan

Carnitine palmitoyltransferase-I (CPT-I) plays an important role in mitochondrial membrane carnitine cycle through the formation of long-chain acylcarnitines from carnitine and acyl-CoAs derived from long-chain fatty acids. The patients with CPT-I deficiency show hypoketotic hypoglycemia during infancy and are detected by newborn screening using tandem mass spectrometer (MS/MS) with a screening marker of the ratio of free carnitine to long-chain acylcarnitines and such confirmation tests as enzyme assay or gene analysis are necessary. Thus, we evaluated whether MS/MS measurement of deuterium-labeled acylcarnitines in peripheral blood mononuclear cells (PBMCs) after the load of deuterium-labeled palmitic acid (d31-PA) and deuterium-labeled octanoic acid (d15-OA) was effective to diagnose CPT-I deficiency. PBMCs, collected heparinized blood, were incubated for 2 hours together with d31-PA and d15-OA. The washed PBMCs were homogenized in methanol and the homogenate was measured by precursor ion scanning using Sciex API-4000 LC-MS/MS system. Although less amounts of deuterium-labeled palmitoylcarnitine (d31-C16), which was formed from d31-PA by CPT-I, were detected in PBMCs from patients with CPT-I deficiency than those from controls, the decreased levels varied considerably according to the number and viability of the collected PBMCs. Otherwise, the ratios of deuterium-labeled butanoylcarnitine (d7-C4)/d31-C16 were significantly higher in patients with CPT-I deficiency than in controls. While d31-C16 is hardly formed in PBMCs from patients with CPT-I deficiency, d7-C4 is normally formed both in patients with CPT-I deficiency and in controls from d15-OA, which is transported into mitochondria without the action of CPT-I and is metabolized thorough beta-oxidation. In conclusion, in our simple loading test using MS/MS together with d15-OA and d31-PA, the ratio of d7-C4/d31-C16 in PBMCs, which is hardly influenced by cell number or viability, is used as a useful marker to diagnose CPT-I deficiency.

### P80. Neonatal Screening for Carnitine Palmitoyltransferase II Deficiency in Japan Using (C16 + C18:1)/C2 and C14/C3

TajimaGoHaraKeiichiTsumuraMiyukiKagawaReikoUtsunomiyaAkariOkadaSatoshiYuasaMioriHataIkueShigematsuYosukeYamaguchiSeijiNational Center for Child Health and Development, Division of Neonatal Screening, Tokyo, Japan

Background: Carnitine palmitoyltransferase (CPT) II deficiency is one of the most common forms of mitochondrial fatty acid oxidation disorder. Among Caucasians, there is a highly prevalent variant of CPT2 gene, p.S113L, which is known to cause myopathic symptoms and patients of this phenotype is reportedly difficult to detect by neonatal screening (NS). Comparatively, the ratio of hypoglycemic phenotype is apparently higher in Japan, where a reliable method of NS for this potentially fatal disease has been anticipated.

Methods: Dried blood specimens (DBS) were collected on 4th or 5th day after birth. Based on our experience of an infantile case of CPT II deficiency presenting hypoglycemic encephalopathy which had been missed in the NS using C16 and C18:1 concentration as indices, we adopted the (C16 + C18:1)/C2 ratio (cutoff 99.9%ile) as an alternative primary index. Positive cases were assessed by enzymatic and genetic analysis.

Results: The disease was diagnosed in seven of 21 NS-positive subjects. The values for (C16 + C18:1)/C2 of the affected newborns partly overlapped with those of the non-affected. Among several other indices proposed previously, C14/C3 differentiated the affected patients from the non-affected. Based on these findings, the nationwide NS for CPT II deficiency using (C16 + C18:1)/C2 and C14/C3 as indices (both cutoffs 99.9%ile) started in April 2018. By April 2019, the total number of NS-positive subjects we examined reached 40 and CPT II deficiency was diagnosed in 13. The frequent variants were p.F383Y and p.E174K, both had been reported in fatal Japanese patients. However, we diagnosed the disease in four little children showing rhabdomyolytic symptoms without hypoglycemia, whose values for these two indices in newborn DBS were far below the cutoffs.

Conclusion: Though it seems still difficult to find patients of myopathic-type CPT II deficiency by NS without fail, our system is expected to save many affected children in Japan where hypoglycemic type of the disease apparently predominates.

### P81. Clinical Data and Gene Mutation Analysis in Carnitine Palmitoyl Transferase 1A Deficiency Patients

HanLianshuGuXuefanXinhua Hospital, Shanghai Jiaotong University School of Medicine, Department of Pediatric Endocrinology and Genetic, Shanghai Institute for Pediat, Shanghai, China

Objective To analyze the clinical characteristics and gene mutations of patients with carnitine palmitoyl transferase 1A deficiency (CPT1AD), to improve the doctors’ understanding of the disease.

Methods The clinical data and biochemical data of the children were collected. The blood free carnitine and acylcarnitines was detected with tandem mass spectrometry and CPD1A gene in children and their parents were detected with PCR.

Results One of 4 children was found by neonatal screening with no clinical symptoms. The remaining 3 cases were acute onset induced by fever, vomiting and diarrhea. The acute phase of case 1 is characterized by convulsion, coma with feeding intolerance and mental retardation development; case 2 acute stage manifested as convulsion, jaundice, limb weakness, abnormal respiration and coma with delayed motor; the acute phase of case 3 showed convulsion, hepatomegaly and hypoglycemia and the blood glucose as low as 0.1mmol/L. In all 4 patients C0 increased, C16, C18 decreased and C0/(C16+C18) increased. There are 7 mutations in CPT1A gene by analyzing (c.281+1G>A, c.693+1G>A, c.968-8C>T, c.946C>T (p.R316W), c.956G>T (p.G319V), c.1787T>C (p.L596P), c.2201T>C (p.F734S). At present, by avoiding hunger and dietary guidance follow-up, case 1 and case 2 have mental retardation and case 3 and case 4 are the same as normal healthy children.

Conclusion The C0 increased, C16, C18 decreased and C0/(C16+C18) increased in acylcarnitines with MS/MS has a great value in diagnosis of CPT1AD. Neonatal screening is helpful to the early diagnosis. This research has found 7 pathogenic mutations in CPT1A gene, which enriches the CPT1A gene mutation spectrum and provides evidences for genetic counseling of the patient’s pedigree and prenatal diagnosis.

### P82. Clinical, Biochemical and Genetic Heterogeneity in Carnitine Palmitoyltransferase 1A(CPT1A) Deficiency

ZhengJingShenYapingXuYanhuaWuDingwenYangRulaiThe Children’s Hospital Zhejiang University School of Medicine, Department of Genetics and Metabolism, Hangzhou, China

Objectives: To investigate the genetic, biochemical and clinical characteristics of CPT1A patients and to explore the genotype to phenotype relation.

Methods: The blood samples of 3,040,815 newborns in Neonatal Screening Center of Zhejiang Province were collected during January 2009 to December 2018 and then screened for genetic metabolic diseases by tandem mass spectrometry (MS/MS). CPT1A deficiency was defined by the presence of elevated ratio of C0/C16+C18 acylcarnitines (increased C0 with low or normal long-chain acylcarnitines) in plasma and further confirmed by genetic analysis of CPT1A gene. The CPT1A variants obtained by sequencing were be classified based on ACMG standards and guidelines. The prognosis and follow-up of patients with CPT1A deficiency were also evaluated.

Results: A total of 12 cases (4 females and 8 males) of CPT1A deficiency from 10 families were diagnosed, with a birth-prevalence of at least 1:253 401. Elevated levels of C0 and decreased C18 as the most prominent species were observed in all of our patients. The average C0 level of was 129.46 ± 61.99 μmol/L, C16 was 0.43 ± 0.27, C18 was 0.17 ± 0.07 C0/C16+C18 was 312.28 ± 278.53. Molecular analysis showed 14 CPT1A variants (13 novel) including 1 frameshift mutation, 1 splicing mutation and 12 missense mutations were identified among the 11 patients. In addition, 2 patients were homozygous genotype, 7 patients were compound heterozygous genotype. For the two remaining patients, only one variant in a heterozygous state was detected. During 5–28 month follow-up, all of these subjects with routine treatment remained asymptomatic and had normal intelligence and physical development.

Conclusions: Although the clinical and biochemical features of these affected subjects identified in newborn screening program were rather homogeneous, the CPT1A mutations they carried were quite differently. Early intervention and management was very effective and important for CPT1A patients.

### P83. Peripheral Blood Level of Carnitine in Neonates and Influencing Factors

TangHuaHunan Maternal and Child Health Hospital, Medical Genetics, Changsha, China

Objective To determine the peripheral blood level of carnitine in neonates and to analyze its influencing factors.

Methods All newborns (*n* = 46250) born in Hunan provincial maternal and child healthcare hospital from 2015 January 1 to 2017 December 31 were included in this study, except for those primary carnitine deficiency and motherhood carnitine deficiency. Heel prick blood samples were collected after 48 hours after birth for determination of carnitine by tandem mass spectrometry. All subjects were grouped according to gender, gestational age, birth weight, number of pregnancies, sample collecting time and whether to be hospitalized. The level of carnitine was analyzed with Mann-Whitney U test, Kruskal-Wallis H test and multiple linear regression.

Results Multivariate linear regression analysis showed that carnitine was influenced the most by whether to be hospitalized, birth weight, gestational age, number of pregnancies, gender and days on blood sampling.

Conclusion The peripheral blood level of carnitine in neonates is affected by many factors, such as whether to be hospitalized, whether to be hospitalized.

**Keywords:** carnitine; neonate; tandem mass spectrometry

### P84. Genetic Analysis in Abnormal Metabolism of 3-Hydroxyisovalerylcarnitine

WuDingwenLuBinYangJianbinYangRulaiHuangXinwenTongFanZhengJingZhangTingShenYapingZhaoZhengyanThe Children’s Hospital Zhejiang University School of Medicine, Department of Genetics and Metabolism, Hangzhou, China

Objective To investigate the genetic characterization of 3-hydroxyisovalerylcarnitine (C5-OH) metabolic abnormality and provide valuable information for clinical diagnosis and therapy in neonates.

Methods The increased values of C5-OH, C5-OH/C3 and C5-OH/C8 by tandem mass spectrometry from 52 neonates screened were collected and calculated. Genomic DNA was extracted from the whole blood with EDTA anticoagulant of all participants in our cohort. Seventy-nine genes associated with genetic and metabolic diseases including MCCC1, MCCC2, HMGCL, AUH, DNAJC19, HLCS, BTDA and CAT1, were targeted by liquid capture technology. Variation information of these genes was obtained by high-throughput sequencing and bioinformatic analysis and then was classified based on ACMG standards and guidelines. Furthermore, these 52 neonates were divided into four groups including wild-type, MCCC1-maternal-mutation, MCCC1-paternal-mutation and MCCC2-mutation according to their genotypes. Statistical analysis by Wilcoxon rank-sum test was carried out for the increased multiples of C5-OH calculated in neonatal screening. Differences were considered significantly at a *p* < 0.05.

Results We identified 21 MCCC1 variants (14 novel) in 37 cases, 6 MCCC2 variants (5 novel) in 4 cases. The increased multiples of C5-OH calculated in MCCC1-maternal-mutation and MCCC2-mutation groups were significantly higher than that in wild-type group (*p* < 0.05), while there was no significant difference between MCCC1-paternal-mutation group and wild-type group (*p* > 0.05).

Conclusion 1. Mutations on MCCC1 and MCCC2 genes are the major genetic causes for the increased C5-OH in neonatal screening. 2. Paternal single heterozygous mutation on MCCC1gene only leads to a mildly increased C5-OH, while maternal single heterozygous mutation can contribute to the moderately to severely increased C5-OH and so the maternal MCCD should be excluded.

**Keywords:** 3-hydroxyisovalerylcarnitine; genetic testing; gene; mutation; neonatal screening

### P84a. Neonatal Screening on Tandem Mass Spectrometry as a Powerful Tool for Emerging and Underestimated Diseases in Newborns and Their Parents: The Experience of Catania Screening Center

MessinaMaria AnnaRaudinoFedericaIacobacciRiccardoMeliConcettaFiumaraAgataExpanded Newborn Screening Laboratory, Catania, Italy

In 2017 the autonomous region of Sicily adopts the national law on mandatory expanded newborn screening (ENS) and sets 1 December 2017 as starting date.

Herein we report the achievements of the Catania screening center, focusing on the power of expanded screening to bring to light emerging and underestimated diseases in babies and in their parents.

The ENS is performed using the NeoBase2 kit on QSight 210MD screening system, a new high-throughput tandem mass platform.

In 21 months, we screened more than 34,000 babies and we diagnosed 7 hyperphenylalaninemia, 1 citrullinemia type I, 1 glutaric acidemia type I, 1 propionic acidemia, 1 isovaleric acidemia and 4 short chain acilCoA dehydrogenase deficiency (SCAD).

As known, SCAD involves accumulation of butirylcarnitine (C4), however of the 40 recalled babies only four shown ethylmalonic acid in urine. Genetic analysis carried out on their parents and siblings confirmed one father and one sister homozygous. One recalled baby did not show ethylmalonic acid in urine but its father shown high value of C4 and elevated excretion of ethylmalonic acid, resulting affected.

The Italian report disclose an incidence for SCAD of 1:46,576 but our data show a significant SCAD underestimation.

Five babies out of 53 recalled babies for propionylcarnitine build up, did not show any acid excretion but parents investigation disclosed a maternal deficit of B12 vitamin.

Finally, high values of 3-OH-isovalerylcarnitine (C5OH) leaded on misdiagnosis of 3-methylcrotonylcarboxilase deficiency, since the babies resulted heterozygous with persistent C5OH high values and 3-OH-isovaleric acid excretion.

Our results demonstrate as ENS is a powerful tool to disclose particular metabolic condition, which represent a new challenge for clinicians and biochemists.

G. Lysosomal Storage Disorders

### P85. Newborn and High-Risk Screening of Gaucher Disease in Japan

SawadaTakaakiMomosakiKenKidoJunMatsumotoShirouEndoFumioNakamuraKimitoshiKumamoto University, Pediatrics, Kumamoto, Japan

Background: Gaucher disease (GD) is an autosomal recessive lysosomal storage disorder caused by the deficiency of glucocerebrosidase enzyme activity. In Japan, enzyme replacement therapy has been available for all subtypes of GD, even if they have acute neurological symptoms. However, as GD is rare and patients may show heterogeneous clinical symptoms, many patients are either misdiagnosed or remain undiagnosed. So, we conducted two approaches to detect GD patients, newborn screening and high-risk screening.

Methods: Both screening methods have been performed using an enzymatic assay on the dried blood spot (DBS) samples. Subjects with low β-glucocerebrosidase (GBA) activity on DBS were analyzed for mutations in GBA by using long-range PCR and next-generation sequencing technology.

Results: In newborn screening, we measured GBA activity on DBS of 37,841newborns and re-examined six samples with low enzyme activity. Among them, we confirmed two GD patients by analysis of GBA mutations. In high-risk screening, we measured GBA activity of 102 patients with various neurological symptoms, which might be suggestive of neuronopathic GD. We re-examined six patients with low enzyme activity and two patients were diagnosed with GD by gene analysis. We started ERT and their hematological and visceral symptoms had been reduced with the exception of one lethal type patient.

Conclusions: In both screening methods, we could detect GD patients and lead to treatment using ERT. Newborn screening can find GD patients to be born and high-risk screening may find undiagnosed neuropathic GD patients. As a relatively high incidence of neuronopathic GD has been reported, this combination of screening methods may be effective in Japan.

### P86. Multiplex-Measurement of Enzyme Activity for Lysosomal Storage Disorders Using LC-MS/MS

MashimaRyuichiOhiraMariOkuyamaTorayukiNational Center for Child Health and Development, Clinical Laboratory Medicine, Tokyo, Japan

Background: Lysosomal storage disorders (LSDs) are characterized by the accumulation of lipids, glycolipids, oligosaccharides, mucopolysaccharides and other biological substances because of the pathogenic deficiency of lysosomal enzymes. Such diseases are rare; thus, a multiplex assay for these disorders is effective for the identification of affected individuals as a part of neonatal screening.

Methods: In this study, 11 enzyme activities were determined using LC-MS/MS. The activity was calculated based on the concentration of internal standard for each enzyme reaction product. Enzyme reaction was performed at 37 °C for 20 h.

Results: We performed a validation study using dried blood spot (DBS) samples with 100% and 5% enzyme activity for quality control (QC). Under the assay condition, the analytical range, defined as the ratio of the peak area of the enzyme reaction products from the DBS for QC with 100% enzyme activity to that from the filter paper blank sample, was between 14 for GALN and 4561 for GLA. Based on these results, the distribution of the enzyme activity for the 11 LSD enzymes was further examined. Consistent with the previous data, the enzyme activity exhibited a bell-shaped distribution with a single peak. The averaged enzyme activity for the healthy neonates was as follows: GLA, 3.80 ± 1.6; GAA, 10.6 ± 4.8; IDUA, 6.4 ± 2.3; ABG, 8.6 ± 3.1; ASM, 3.3 ± 1.1; GALC, 2.8 ± 1.3; ID2S, 16.7 ± 6.1; GALN, 1.2 ± 0.5; ARSB, 17.0 ± 8.7; NAGLU, 4.6 ± 1.5; and GUSB, 46.6 ± 19.0 mmol/h/L (mean ± SD, *n* = 200). In contrast, the enzyme activity in disease-affected individuals was lower than the minimum enzyme activity in healthy neonates.

Discussion: The results demonstrate that the population of disease-affected individuals was distinguished from that of healthy individuals by LC-MS/MS-based technique.

### P87. Successful Newborn Screening for Gaucher Disease Using Fluorometric Assay in Shanghai

ZhangHuiwenXinhua Hospital, Shanghai Jiao Tong University School of Medicine, Pediatric Endocrinology and Genetic Metabolism, Shanghai, China

Background Gaucher disease (GD) is an inherited metabolic disorder that involves accumulation of glycolipid glucocerebroside in monocyte-macrophage cells, which can result in multiple organ damage. Enzyme replacement and substrate reduction therapies have improved the potential for early diagnosis and treatment. Determining the true incidence of this rare disease is critical for relevant policy establishment. Newborn screening allows for early diagnosis and a comparatively accurate incidence of GD.

Methods A fluorometric method to detect acid β-glucocerebrosidase (GBA) activity on a dried blood spot punch was developed. Validity and feasibility of the fluorometric method was demonstrated by examining 116 healthy controls, 19 confirmed GD patients and 19 obligate carriers. GBA activity was measured on dried blood spots of 80,855 newborns. Samples from positively screened newborns were reanalyzed by a leukocyte GBA activity test and GBA gene analysis. Plasma glucosylsphingosine level was determined as a biomarker of the pathophysiology of GD.

Results GD patients were distinguished from healthy controls and obligate carriers using the fluorometric method. Mean GBA activity in newborn screening specimens was 145.69 ± 44.76 μmol/L/h (*n* = 80,844). Three children had low GBA activity, of which one child had low GBA activity on the second dried blood spot specimen. Leukocyte, genetic and biomarker analysis confirmed the diagnosis and indicated that this child was in the early stages of GD.

Conclusions Screening for GD by fluorometric analysis of GBA activity is an efficient and feasible technology in newborns.

### P88. Clinical, Biochemical Analysis and Genetic Features of Four China Case with Mucopolysaccharidosis IIIC

GaoXiaolanXinHua Hospital, Pediatric Endocrine, Shanghai, China

Objective Mucopolysaccharidosis type III (MPS III), known as Sanfilippo syndrome, which is a kind of autosomal recessive genetic disease, caused by the lack of four subtypes of enzymes that does not completely degrade acidic mucopolysaccharides (glycosaminoglycans GAGs), which finally leading to a lysosomal storage disease. The main component of GAGs in the urine sample of this case is heparan sulphate HS, which can be divided into type A, B, C and D clinically, of which type C and D are rare. This article introduces four domestic cases of MPS IIIC (acetylcoenzyme A: α-glucamine N-acetyltransferase, OMIM252930) patient.

Results In four patients, the concentration of GAGs in urine was significantly increased and the heparin sulfate band (HS) was found in the results of urine mucopolysaccharide vinegar fiber film electrophoresis, also, the results of leukocyte zymography were lower than the normal reference values. Patient 1: HGSNAT mutation was c. 4931G>A; Patient 2: Two known complex heterozygous variants found respectively c. 234 1G>A and c. 1150C>T, p. Arg384A; Patient 3: Found compound heterozygosity: c371G>C, pArg124Thr; c. 1030C>T, p. Arg344Cys. c 371G>C, predicted by the software to affect splicing, which requires enzymology verification; Patient 4: Two complex heterozygous variants c1743delG, P.v582Yfsx18c. 869G>C, p. C290A, P. C290A is a missense mutation with a very low frequency in the population and this locus is not a polymorphic locus, which has not been reported in HDMG professional data.

Conclusions There has not been MPS IIIC reported in China so far. MPS IIIC in our Lab to detected by enzymology for approximately 0.88% of MPS patients in the past years. In this study, we reported the clinical and biochemical analysis of four cases of MPS IIIC in China and the results of screening for hgsnat gene mutations.

**Keywords:** mucopolysaccharidosis type III; heparan sulphate (HS); acetyl coenzyme A: alpha glucosaminide acetyltransferase; hgsnat gene mutation

### P89. Review of Prospective Newborn Screening Results for Lysosomal Storage Disorders

TherrellBradfordDirector, National Newborn Screening and Global Resource Center, Professor of Pediatrics, University of Texas Health Science Center at San Anton, Austin, TX, USA

As countries investigate adoption of newborn screening for lysosomal storage disorders (LSDs), several factors need to be taken into consideration. First, prospective, full-population screening results are available and need to be evaluated to compare the performance of screening methodologies. Two platforms are currently deployed for LSD enzyme testing in dried blood spot specimens—digital microfluidic fluorometry (DMF) and tandem mass spectrometry (MS/MS). Both platforms utilize synthetic substrates but use a different detection methodology. DMF was cleared by FDA in 2017 and more recently MS/MS reagent kits obtained FDA clearance; both have CE-marking.

The ability of each method to discriminate normal from affected samples must take into account the multiple sources of variability (biological/genetic, sample quality, gestational age) that exist between samples. Significant data sets are available from several U.S. states and other countries including Taiwan. The data sets do not show significant differences between the two platforms in terms of the positive predictive value (PPV) though there are some noticeable differences in false positive rates. It is clear, however, from these and other prospective data, that additional measures are required to improve the PPV of LSD screening tests.

That said, newborn screening programs need to carefully evaluate their needs in light of the differences between platforms. Differences include cost of installation, maintenance, ease of use, testing throughput, cross training of technicians and facilities upgrades. We conclude that prospective LSD screening programs in the United States, Taiwan and other countries are excellent, unbiased resources for a comprehensive evaluation of suitability for a newborn screening program. The emerging clinical results reflect the real-world performance of each platform and combined with all other practical issues, the associated costs and workflow also need to be taken into account to determine the best fit.

### P90. Clinical Performance Evaluation of the NeoLSD™ MSMS kit for Lysosomal Storage Disorder (LSD) Newborn Screening

LindroosHanneVaahteraKatjaCohenArielThorbekMarta JLundAllan MKantolaJaanaMattssonPekkaMeierjohannAxelHougaardDavid MPerkinElmer, Diagnostics, Turku, Finland

The NeoLSD™ MSMS Kit is a new multiplex assay for the quantitative measurement of the activity of the enzymes acid-β-glucocerebrosidase (ABG), acid-sphingomyelinase (ASM), acid-α-glucosidase (GAA), β-galactocerebrosidase (GALC), α-galactosidase A (GLA) and α-L-iduronidase (IDUA) in dried blood spots (DBS) from newborn babies. The analysis of the enzymatic activity is intended as an aid in screening newborns for the following lysosomal storage disorders (LSD) respectively; Gaucher Disease, Niemann-Pick A/B Disease, Pompe Disease, Krabbe Disease, Fabry Disease and MPS I Disease. This assay is the first IVD kit bearing CE mark and cleared by FDA to screen newborns for the 6 LSDs.

The objectives of the clinical performance study were to produce newborn population distribution data for each of the 6 enzymes using the NeoLSD™ MSMS assay and demonstrate that the subjects affected with any of the screened disorders can be distinguished from the normal population by using the site-specific cut-offs derived from the population distribution data.

The specimens were archived newborn screening dried blood spot samples from the Danish Newborn Screening Biobank collected from 30 affected—5 Gaucher, 1 Niemann-Pick A, 10 Krabbe, 5 MPS I, 5 Fabry and 4 Pompe—and approximately 4000 unaffected newborns. Clinical outcome was obtained from all samples and used as a comparator for the NeoLSD™ MSMS assay.

NeoLSD™ MSMS kit was found to correctly identify affected subjects with low lysosomal enzyme activity from the normal population. The sensitivity of the NeoLSD™ MSMS assay was 92.9% and 100%, when two screen negative Fabry samples were included and excluded, respectively. The specificity of the assay was 99.4%, leading to 0.6% false positive rate.

### P91. Tier 1 and Tier 2 Mass Spectrometry Methodologies for Determining Enzymatic Defects Associated with Krabbe Disease

HillCollinPerkinElmer, Diagnostics, Waltham, MA, USA

Krabbe disease is a rare lysosomal storage disorder caused by a defect in the galactosylceramidase (GALC) enzyme, rendering it unable to break down galactolipids which are abundant in the brain. It is believed that the accumulating galactolipids, namely psychosine, contribute to the destruction of myelinating oligodendrocytes, resulting in severe neurological disfunction. Therefore, it is crucial to detect Krabbe during the early days of life, during a critical development window for neonates. Here we describe two mass-spectrometry based assays which determine GALC activity through first tier rapid screening and subsequent confirmatory analysis of psychosine using LC-MS/MS in contrived positive samples.

### P92. Tier 1 Screening for Enzyme Deficiencies Associated with GAMT (Guanidinoacetate Methyltransferase) Using Flow-Injection MS/MS

HillCollinPerkinElmer, Diagnostics, Waltham, MA, USA

GAMT deficiency is a severe neurological disorder, caused by the toxic accumulation of guanidinoacetate (GAA), resulting in impaired cognitive and motor development. While no therapies for GAMT deficiency have been approved, research has shown that the physiological amino acid ornithine is a competitive inhibitor for AGAT, which produces the neurotoxic GAA. Thus, it is crucial to diagnosis GAMT deficiency during the early days of life, a critical neurological development window for neonates. Here we describe a flow-injection, non-derivatized method for detection of GAA in contrived positive dried blood spots (DBS). Briefly, blood was spiked with clinically relevant concentrations of GAA to mimic contrived positive samples for GAMT deficiency. Dried blood spots were then made by spotting blood onto filter paper, allowing it to dry, followed by subsequent extraction of GAA. A PerkinElmer QSight^®^ 210 MD mass spectrometer was used, for research purposes only, to demonstrate the capability of the assay. Contrived positive DBS with elevated GAA were successfully flagged via the tier 1 methodology and separated from presumed negative DBS. We conclude that the assay and instrumentation used here can accurately screen for enzymatic defects associated with GAMT, as done in a newborn screening laboratory.

### P93. Tier 2 Resampling from Contrived Positive DBS for X-ALD (X-Linked Andrenoleukostrophy) Using the QSight^®^ 225 MD

HillCollinPerkinElmer, Diagnostics, Waltham, MA, USA

Newborn screening labs are responsible for the rapid testing of several hundreds, if not thousands of samples a day for various inborn errors of metabolism. Typically, this is done by a flow-injection MS/MS approach (Tier 1) which allows for quick turnaround of clinical data. While the incidence of flagged potential positives for any disease is very low, those that are flagged must be retested via a separate Tier 2 approach. often, this is done with a longer chromatography-based methodology which is capable of separating out interfering isobars before the biomarker of interest is introduced into the mass spectrometer. Retesting of these samples often becomes a bottleneck, as the samples must be re-prepared and then subjected to subsequent analytical tests, which adds time, complexity and room for human error to the process. Here, we demonstrate an automated workflow using the PerkinElmer QSight^®^ 225 MD (in development) mass spectrometry system, where samples that are positive for tier 1 markers are automatically retested via tier 2 methodologies, without the need for human intervention. Contrived positive samples for both tier 1 and tier 2 markers were made and subjected to the automated methodologies described here. Examples of diseases associated with enzymatic effects tested include Krabbe disease and X-linked adrenoleukodystrophy. This approach allows for quicker retesting via tier 2, resulting in important clinical data being delivered to health care professionals in a timelier fashion and greatly reduces the risk of human error during sample transport and reinjection. This is of particular importance in the newborn screening community where time is critical and confirmation of disease status is required before significant onset of pathogenesis.

### P94. Tier 1 and Tier 2 Mass Spectrometry Methodologies for Determining Enzymatic Defects Associated with Pompe Disease

HillCollinPerkinElmer, Diagnostics, Waltham, MA, USA

Pompe disease is a rare lysosomal storage disorder caused by a defect in the GAA enzyme, rendering it unable to break down glycogen. In particular, glycogen accumulates in skeletal and cardiac tissue, resulting in neuromuscular degeneration and cardiomyopathy. Enzyme replacement therapy has proven to be an effective treatment, with the Lumizyme^®^ product, especially if administered before pathogenesis of the disease. Therefore, it is crucial to detect Pompe disease during the early days of life, a critical development window for neonates. Here we describe two mass-spectrometry based assays which determine GAA activity through first tier rapid screening and subsequent confirmatory analysis of tier 2 markers ((creatine/creatinine)/GAA) using LC-MS/MS in contrived positive samples.

Contrived positive dried blood spots (DBS) were made by using leukocyte depleted red blood cell concentrate, spiked with creatine and creatinine. Presumed negative DBS were made by using whole blood, spiked with creatine and creatinine. These were first subjected to an enzymatic assay, followed by a rapid flow-injection based MS/MS to distinguish contrived positives from presumed negative DBS for Pompe. Flagged contrived positives were then subjected to a separate tier 2 LC-MS/MS assay capable of separating interfering isobars, to screen for elevated ((creatine/creatinine)/GAA) ratios. Briefly, for tier 2 testing, DBS were extracted for creatine, creatinine and GAA, then analyzed against a standard curve, in order to identify contrived positives. A PerkinElmer QSight^®^ 220 CR instrument was used for analysis.

Here, we demonstrate that the activity-based flow-injection MS method is able to determine deficient GAA activity in contrived positives for Pompe disease. The second tier LC-MS/MS assay is capable of flagging contrived positives with high, clinically relevant ((creatine/creatinine)/GAA) ratios. With this approach, we demonstrate an LC-MS instrument which is capable of both tier 1 and tier 2 testing for Pompe disease.

### P95. Multiplexed Tier 1 and Tier 2 Mass Spectrometry Methodologies for Determining Disorders Associated with Inborn Errors of Lipid Metabolism

HillCollinPerkinElmer, Diagnostics, Waltham, MA, USA

Neonatal screening labs are tasked with screening a vast amount of samples from newborns to flag those that may harbor an inborn error metabolism. The number of possible diseases to screen for can be quite large, making multiplexed analytical techniques such as mass spectrometry attractive. Here, we describe a tier 1 flow injection assay to identify contrived positive samples. Subsequently, a chromatography tier 2 multiplexed method for acylcarnitines was employed as a confirmatory assay. Both assays used a PerkinElmer™ QSight^®^ 220 CR mass spectrometer. Using this approach, contrived positives were flagged, as would be done in a neonatal screening lab.

### P96. Seven-Plex MS/MS Method to Measure I2S, NAGLU, GALNS, GLB1, ARSB, GUSB and TPP1 Enzyme Activities in DBS on a PE QSight^®^ 220 CR

HeTaoTrometerJoeHillCollinTimmonsMikeYeungTsun AuWilliamsonBrianPerkinElmer, Diagnostics, Turku, Finland

The mucopolysaccharisodes (MPS) family of lysosomal storage disorders (LSDs) is caused by defects in the metabolic breakdown of glycosaminoglycans (GAGs). This work demonstrates a novel MS/MS assay that simultaneously monitors the activity of seven different MPS enzymes. Using a single 3.2 mm dried blood spot (DBS) punch and incubation cocktail, our assay has the ability to identify samples with low enzyme activities for I2S (MPS II), NAGLU (MPS IIIB), GALNS (MPS IVA), GLB1 (MPS IVB), ARSB (MPS VI), GUSB (MPS VII) and TPP1 (CLN2). The seven-plex is incubated overnight in the presence of incubation cocktail at 37 °C followed by a post-incubation, fully automated workup that is less than 30 minutes per plate. Enzyme activities are measured by LC-MS on a PE QSight^®^ 220 CR triple-quad Mass Spectrometer. Sample-to-sample time using MS/MS analysis can be as low as 2 minutes, which allows the possibility to obtain more than 5000 results per day if desired.

Method performance studies show good linearity for each enzyme in their respective activity range. Furthermore, a study consisting of several hundred presumed healthy neonates, confirmed low I2S, NAGLU, GALNS, GLB1, ARSB, GUSB, and/or TPP1 activity and CDC control DBS showed excellent resolution and clear distinctions between the different enzyme activity levels.

### P97. A Rapid Multi-Plex MS/MS Assay for Lysosomal Storage Disorders

HeTaoTrometerJoeHillCollinTimmonsMikeYeungTsun AuWilliamsonBrianPerkinElmer, Diagnostics, Turku, Finland

A new multiplex flow injection analysis—tandem mass spectrometry (FIA-MS/MS) method is described that simultaneously measures the activities of the enzymes ABG, ASM, GAA, GALC, GLA and IDUA, using a single 3.2 mm punch from a dried blood spot (DBS). The currently accepted methodology for multi-plex assays involving the GALC enzyme calls for an overnight incubation step. This study examines the effects on all enzymes of a shortened incubation time on the ability to discriminate between positive and negative samples.

The multi-plex method was tested with DBS from 2 groups of presumed healthy subjects (each *n* = ~700), contrived positive samples having low activities and DBS controls. The analytical analysis was done on a PE QSight^®^ 210 MD triple-quad Mass Spectrometer. The data shows a robust difference between the enzyme activities of the presumed healthy subjects from those with confirmed low activities with acceptable signal-to-noise and precision.

### P98. High-Risk Screening for Gaucher Disease *via* Dried Blood Spot in China

JiaXuefangFengYiHuangYonglanGuangzhou Women and Children’s Medical Center, Department of Guangzhou Newborn Screening Center, Guangzhou, China

Objective: Gaucher disease (GD), a relatively common lysosomal storage disorder, is caused by the deficiency of the lysosomal enzyme glucocerebrosidase (GCase) due to the mutant GBA gene. However, the nonspecific symptoms of GD and the inconvenient diagnostic approach often lead to delayed diagnosis in those patients. To evaluate the enzymatic assay on dried blood spot (DBS) and to provide further experimental evidence, the screening program for GD was therefore conducted.

Participants and methods: Totally 436 subjects (195 females, 225 males and 16 subjects with gender unknown) with hepatosplenomegaly and/or hematological changes from China were enrolled. DBS samples were collected from this high-risk population. Activities of GCase and chitotriosidase were measured by fluorescent substrate assay. Then the GCase activity in the leukocyte was assayed and the GBA gene was sequenced by Sanger sequencing in those with positive screening results.

Results: A total of 88 subjects were tested positive on the screening assay and eventually 26 GD patients were confirmed among the 436 high-risk subjects, which yielded an incidence of 6% and a specificity of 84.9%. Through the molecular analysis of the GBA gene, 24 variants were found, including seven novel variants, extending its variant spectrum.

Conclusions: This work demonstrated that screening of GCase using dried blood spot method is an important tool to facilitate the early diagnosis of GD patients suffering from splenomegaly and/or thrombocytopenia. These results will be helpful in establishing a systematic screening program for the high-risk GD patients in China.

H. Haemoglobinopathia

### P99. Analysis of 30,554 Neonatal Disease Screening of Thalassemia in Huangshi City

KeHaiyanJiangHongHuangshi Maternity and Children’s Health Hospital, Laboratory Medicine, Huangshi, China

Objective: To investigate the status of thalassemia gene carrying in Huangshi city and its different genotypes and clinical phenotypes.

Methods: The initial screening of thalassemia was conducted by capillary electrophoresis and the positive screening was recalled for thalassemia gene and blood routine test.

Results: In 2018, there were 31,140 live births in Huangshi city, among them, 30,554 neonatal disease screening were carried out, with a screening rate of 98.12% and a descent-positive rate of 1.79%. α-thalassemia accounted for 73.44%, including -SEA/αα (54.11%), -α 3.7/α(37.66%),-α 4.2/α(6.48%), light 221 cases, stationary 177 cases and middle type 3 cases. β-thalassemia accounted for 26.01% and 13 types of mutations were detected. The top three genotypes were: IVS-2-654(50.7%), CD41-42(19.72%), CD17(8.45%), 133 light cases and 9 intermediate cases. There were 3 cases of αβ-thalassemia and 2 cases of Southeast Asian missing α-thalassemia type combined with Hong Kong type HK αα. The incidence of anemia in α-thalassemia is 21.94% and 78.41% of slight anemia type; the incidence of anemia in β-thalassemia is 42.25%, mild type accounts for 63.33%, the incidence of anemia gene deficiency was 27.29% and mild type accounts for 76.43%.

Conclusion: The rate of thalassemia in Huangshi city is 1.79%, which is mainly α-thalassemia with -SEA/αα genotype. β-thalassemia is most common with IVS-II-654 genotypes, with mainly static and light clinical phenotypes. For the poor carriers carrying anemia and most of them are mild anemia, β-thalassemia anemia is more likely to occur than α-thalassemia. Above all, it is necessary to conduct thalassemia screening in the newborn population and will help for preventing thalassemia and decrease birth defects.

**Keywords:** capillary electrophoresis; thalassemia; gene types; anemia; clinical phenotype

### P100. Analysis of 30,554 Neonatal Disease Case of Thalassemia Screening in Huangshi City

JiangHongHuangshi Maternity and Children’s Health Hospital, Laboratory Medicine, Huangshi, China

Objective: To investigate the status of thalassemia gene carrying in Huangshi city and its occurrence in different genotypes and clinical phenotypes.

Methods: The initial screening of thalassemia was carried out by capillary electrophoresis and the positive screening was recalled for thalassemia gene and blood routine test.

Results: In 2018, there were 31,140 live births in Huangshi city, among them, 30,554 neonatal disease screening were carried out, with a screening rate of 98.12% and a descent-positive rate of 1.79%. Alpha poverty accounts for 73.44%, including -SEA/αα (54.11%),-α 3.7/α(37.66%),-α 4.2/α(6.48%), light 221 cases, stationary 177 cases and middle type 3 cases. Beta poverty accounted for 26.01% and 13 types of mutations were detected. The top three were genotypes were: IVS-2-654 (50.7%), CD41-42 (19.72%), CD17 (8.45%), 133 light cases and 9 intermediate cases. There were 3 cases of α, β compound poverty and 2 cases of Southeast Asian missing α-poverty type combined with Hong Kong type HK αα. The incidence of anemia in alpha poverty is 21.94% and 78.41% of slight anemia type; the incidence of anemia in beta-poverty is 42.25 %, mild type accounts for 63.33%, the incidence of anemia in the population with 27.29% gene deficiency and mild type accounts for 76.43%. There is no severe anemia.

Conclusion: The rate of poverty in Huangshi city is 1.79%, which is mainly alpha poverty with -SEA/αα genotype. Beta poverty is most common with IVS-II-654e genotypes, with mainly static and light clinical phenotypes. Though a quarter of the poor carriers have the possibility of anemia and most of them are mild anemia, beta-poor anemia is more likely to occur than alpha poverty. Above all, it is necessary to conduct thalassemia screening in the newborn population. Through the screening of thalassemia, it will make measures for preventing thalassemia and decrease birth defects.

### P101. Challenges of Integrating Screening for Hemoglobin Disorders into the Philippine Newborn Screening Program

PadillaCarmencitaAlcausinMelanie Libertyde CastroReynaldoReyesMa. ElouisaJomentoCharitySuarezRiza ConcordiaAbarquezConchitaWinston PosecionJ. EdgarElizagaAnna LeaPanganibanAlmaMendozaBernadetteOtayzaMa. Paz VirgniaTherrellBradfordHoppeCarolynUniversity of the Philippines Manila, Newborn Screening Reference Center, National Institutes of Health, Ermita Manila, Philippines

Background: The Philippine Newborn Screening (NBS) Program began in 1996 and was formalized through the NBS Act of 2004. Six conditions were initially included. With improving coverage and pilot data on expanded NBS, expansion of the screening panel to include additional metabolic conditions and hemoglobin disorders was introduced in 2014.

Objectives: This presentation will briefly review the integration of NBS for hemoglobin disorders (including thalassemias) into the Philippine NBS Program, the challenges and opportunities, as well as the results to date.

Methods: Expansion of NBS to include hemoglobin disorders was aided by experiences from other international programs and experts. The laboratory screening protocol includes initial screening with HPLC, which provides improved detection capability for alpha thalassemias known to be highly prevalent in the country. Confirmatory testing is provided by a central laboratory utilizing capillary electrophoresis with molecular testing available as needed.

Results and Conclusions: NBS in the Philippines now includes screening for 29 conditions including hemoglobin disorders. There were 480 cases with hemoglobin disorders from Dec 2014 to 2018, including over 400 cases of Hb H disease. Over 4000 thalassemia carriers have also been identified. The major challenge is minimizing patient recall for the non-clinically significant conditions. Molecular studies are ongoing to assist in setting appropriate semi-quantitative cutoffs for Hb Barts.

### P102. Neonatal Screening of α-Thalassemia by Capillary Electrophoresis for 78,449 Neonates in Guangxi Region of Southern China

ZhouXunzhaoZuoYangjinLinLiWangLiangChenQiuliHeShengChenBiyanFanXinThe Maternal and Child Health Hospital of Guangxi Zhuang Autonomous Region, Prenatal Diagnosis Center, Nanning, China

Background: The detection of Hb Bart’s (γ4) by capillary electrophoresis (CE) in 3–7 days-old neonates suggested at least one of four α-globin genes are dysfunctional caused α-thalassemia trait or HbH disease. The study aimed to explore the accuracy and sensitivity of Neonatal screening of α-thalassemia by CE.

Method: We collected heel stick sample of 78499 newborns in Guangxi Region from 2016 to 2018 used Capillary’s Hemoglobin Kit to quantify Hb Bart’s. A part of newborns with positive screening results were recalled and then performing thalassemia genetic testing.

Results: There were 8495 newborns who were detected of Hb Bart’s among 78499 individuals. We recalled 1165 positive children among them to do the molecular diagnosis of thalassemia and 1157 of 1165 positive children were diagnosed of α-thalassemia. There were 454 α+ mutation carries (one α-globin gene loss, 39.2%), 656 α0 mutation carries (two α-globin genes loss, 56.7%) and 47 HbH diseases (three α-globin genes loss, 4.1%). The positive coincidence rate of the method was 99.3%. In addition, we found 62 children were diagnosed of α-thalassemia without Hb Bart’s detected. 38 of 62 negative screening newborns were α+ mutation carries, the type of αWSα/αα were especially failed to detect Hb Bart’s and other 24 individuals were α-thalassemia carries compound β-thalassemia Heterozygotes.

Conclusion: The method of CE in neonatal screening of α-thalassemia has the high accuracy of 99.3% and may influenced by lab error, slightly sample contaminated or unknown mutation type unable to identify yet. From the data of the false negative, α+ mutation carries or α-compound β-thalassemia are preferred to be missed especially in some genotype or delayed blood collecting time.

**Keywords:** α-thalassemia; neonatal screening; capillary electrophoresis

### P103. Correlation Between HPLC and Ce Values and Alpha Globin Gene Mutations of Filipino Newborns Screened Positive for Alpha Thalassemia

Tipton SilaoCatherine LynnPadillaCarmencita DavidFabellaTerence DianeMalaqueEmilyne FeElizagaAnna LeaWinston PosecionEdgarAbarquezConchitaMendozaBernadettePanganiban-AndalAlmaOtayzaMaria PazJomentoCharityAlcausinMaria Melanie LibertyHoppeCarolynTherrellBradfordInstitute of Human Genetics, National Institutes of Health, Dept. of Pediatrics, University of The Philippines-Philippine General Hospital, Ermita, Manila, Philippines

Background: Presumptive diagnosis of alpha (α)-thalassemia includes evaluation of hemoglobin levels/variants through High Performance Liquid Chromatography (HPLC) and Capillary Electrophoresis (CE) and mutational analysis of the alpha globin genes, HBA1 and HBA2. The Expanded Newborn Screening (eNBS) Program in the Philippines has included alpha-thalassemia in the additional 22 panel of diseases detected through the program. The screening and confirmatory of this disease were done through HPLC analysis and CE and mutational analysis, respectively. This study aimed to identify the cut-offs of HPLC and CE values in the screening of alpha-thalassemia cases specific to Filipino newborns.

Methods: Evaluation was done through correlation of HPLC and CE values with the HBA1 and HBA2 mutations of the newborns tested. One hundred forty seven (147) and one hundred forty one (141) newborns were included in the correlation of HPLC values and HBA1 and HBA2 mutations and CE values and HBA1 and HBA2 mutations, respectively. Screening cut-offs for HPLC and CE were determined by identifying the lowest Hb Barts value detected with corresponding three (3)-affected/gene deletion of the HBA1 and HBA2.

Results: Hb Barts values of 26.20% and 1.00% were found to be the lowest value with corresponding three (3)-gene affected/deletion of the HBA1 and HBA2 for HPLC and CE analysis, respectively.

Conclusions: This result suggests that Hb Barts values of 26.20% and 1.00% may be used as cut-offs for HPLC and CE analysis in the screening of alpha thalassemias in Filipino newborns. The results may aid in the improvement of the screening of alpha thalassemia cases in the country.

**Keywords:** alpha thalassemia; HPLC; electrophoresis; HBA1; HBA2; mutations; newborns; Filipinos

### P104. Prevalence of Common HBA1 and HBA2 Mutations in Filipino Newborns Screened Positive for Alpha Thalassemia

SilaoCatherine Lynn TiptonFabellaTerence DianeYusonErnestoNaranjoMaria LizaPadillaCarmencita DavidInstitute of Human Genetics, National Institutes of Health, Dept. of Pediatrics, University of The Philippines—Philippine General Hospital, Ermita, Manila, Philippines

Background: Alpha (α)-thalassemia results from the absence/reduced synthesis of the α-globin subunit of hemoglobin. Mutational variants in the HBA1 and HBA2 genes, which code for α-globin, have been reported and cause varying degrees of disease severity. These variants are unique for every population. However, local data on alpha-globin gene mutations in Filipino alpha-thalassemics is currently lacking.

Objective: The Expanded Newborn Screening (eNBS) Program in the Philippines has included thalassemias in the additional 21 panel of diseases detected via the program in December 2015. In response, this study aimed to identify the prevalence of the common α-globin gene mutations in Filipino newborns screened positive for α-thalassemia.

Methods: One hundred (100) newborns underwent DNA extraction and Vienna Lab Alpha Globin Strip Assay mutational analysis.

Results: The (--SEA/αα), (-3.7/--SEA), (-3.7/--FIL), (-4.2/-4.2), (-4.2/--SEA), (-4.2/--FIL) and (--SEA/HbCS) mutations were found in 38.00%, 31.00%, 26.00%, 1.00%, 1.00%, 1.00% and 1.00% of newborns, respectively. The allelic frequencies of the following alleles, (--SEA), (-3.7), (FIL), (-4.2) and (HbCS) were found to be 35.50%, 28.50%, 13.50%, 2.00% and 0.50%, respectively.

Conclusions: This result suggests that the (--SEA/αα), (-3.7/--SEA) and (-3.7/--FIL) mutations are prevalent in Filipino newborns tested. The high prevalence of the (--SEA), (-3.7) and (--FIL) alleles is important to note as these mutations may increase the risk of Hb Bart’s and Hydrops Fetalis cases in the population.

**Keywords:** alpha thalassemia; HBA1; HBA2; mutations; newborns; Filipinos

### P105. Optimization and Application of a Dried Blood Spot-Based Genetic Screening Method for Thalassemia in Shenzhen Newborns

WenWeiGuoMengPengHongbingMaLiShenzhen Maternity and Child Healthcare Hospital, Neonatal Screening Centre, Shenzhen, Guangdong Province, China

Thalassemia is fast becoming a public health concern, inducing a large burden on the healthcare system in many counties, including China. Launching large-scale carrier screening would prevent the birth of children with severe thalassemia. However, these screenings are not compulsory for any individual or population in China. The birth rate of newborns with severe thalassemia remains high; therefore, there is a need for newborn thalassemia screening. Dried blood spots (DBS) are routinely collected from newborns in Chinese hospitals using a heel stick. Using DBS instead of fresh blood makes shipping and storage easier and lowers costs. In this study, we optimized a large-scale neonatal thalassemia genetic screening program in China using DBS and applied the approach to screen newborns

To optimize and apply an approach suitable for large-scale neonatal thalassemia genetic screening in China, thalassemia genotypes were determined by PCR-RDB using DNA extracted from DBS obtained from newborn screening programs. Firstly, the most suitable commercial DNA extraction kit for DBS were screened. Then the appropriate amount of DBS required for the automated high-throughput DNA extraction system was evaluated. Finally, the thalassemia prevalence and genotype spectrum in Shenzhen were investigated in 2028 newborns using the optimized screening procedure.

The Magentec extraction kit was best suited for the automated DBS DNA extraction system using diameter 3-mm DBS discs. The neonatal thalassemia prevalence in Shenzhen was 9.12%; 6.31% α-thalassemia, 2.37% β-thalassemia and 0.44% α-/β-thalassemia.

In conclusion, genetic-screening based on DBS can precisely identifying the thalassemia genotypes. Both α- and β-thalassemia are widely distributed in Shenzhen newborns. Newborn genetic screening is important for establishing a comprehensive thalassemia prevention program and for public education.

### P106. The use of Capillary Electrophoresis for Newborn Screening of Hemoglobinopathies: Preliminary Analytical Performances of CAPILLARYS 3 TERA, SEBIA

ReymondPhilippeClementJean-BaptisteHologneFlorenceGhaouarMaryamDamiotPaulineSimoninDenisRobertFredericSebia, Marketing, EVRY CEDEX, France

Background: Hemoglobinopathies affect 2.7 conceptions per 1000. Numerous countries have implemented NBS for severe hemoglobinopathies, allowing affected children to lead healthy lives. Common techniques (HPLC, IEF and Capillary Electrophoresis) are based on detection of abnormal Hb fractions. Here we describe the analytical performances of the newly developed Hb NBS kit on CAPILLARYS 3 TERA, high-throughput and high-volume CE instrument from SEBIA. Integration into the laboratory workflow and benefits of the solution in term of automation, traceability and interpretation will be also discussed.

Methods: Accuracy of the method was assessed. Effect of storage of the DBS, prematurity and late collection on Hb variants detection were measured. Interferences with acetylated Hb F, triglycerides and bilirubin were assessed. Correlation study with existing CE technique was carried out. Workflow studies as well as ergonomic aspects have been evaluated.

Results: This new CE technique can pick up on newborn all major common Hb variants (Hb S, C, D-Punjab, E, G-Philadelphia, O-Arab, etc.) and thalassemia (Beta-thalassemia major, Hb Bart), at high sensitivity and specificity. No interference or carry-over have been detected. Reproducibility after qualitative identification of Hb fractions is excellent. Agreement between CAPILLARYS 3 TERA and existing CE technique is very good. The high throughput of 70 samples/h, the full traceability from punch to result and the interpretative aids embedded in the software allow easy integration in large NBS centers.

Conclusion: CAPILLARYS 3 TERA is a suitable CE instrument to analyze DBS for hemoglobinopathies newborn screening. Detection of the Hb fractions is accurate. Its good overall analytical performances and its high throughput and loading capacity make it an instrument of choice for high volume NBS laboratories that would perform hemoglobinopathies screening at high degree of confidence.

I. Various (New) Disorders

### P107. Including Classical Galactosaemia in the Expanded Newborn Screening Panel Using Tandem Mass Spectrometry for Galactose-1-Phosphate

CohenAriehBaurekMartaLundAllanDunøMortenHougaardDavidStatens Serum Institut, Congenital Diseases, Copenhagen, Denmark

Galactosaemia has been included in various newborn screening programs since 1963. Several methods are used for screening; however, the predominant methods used today are based on the determination of either galactose-1-phosphate uridyltransferase (GALT) activity or the concentration of total galactose. They cannot be multiplexed and therefore require one full punch per sample. Since the introduction of mass spectrometry in newborn screening, many diseases have been included into newborn screening programs. Here we present a method for including classical galactosaemia in an expanded newborn screening panel based on specific determination of galactose-1-phosphate by tandem mass spectrometry. The existing workflow only needs minor adjustments and it can be run on the tandem mass spectrometers in routine use. Furthermore, compared with currently used methods, the novel method has a superior screening performance producing significantly fewer false positive results. We present data from 5500 routine newborn screening samples from the Danish Neonatal Screening Biobank. The cohort was enriched by including 14 confirmed galactosaemia positive samples and 10 samples positive for other metabolic disorders diagnosed through the Danish newborn screening program. All galactosaemia positive samples were identified by the method with no false positives. Furthermore, the screening performance for other metabolic disorders was unaffected.

### P108. Non-Invasive Screening of Wilson Disease in School Children: A Feasibility Study

TangNelsonHuiJoannieLeungT.F.Chinese University of Hong Kong, Department of Chemical Pathology, Shatin, Hong Kong (China)

Early treatment of Pre-symptomatic Wilson’s disease (WD) is highly effective. However, there is no good non-invasive biomarker available for large scale screening.

Hypothesis: Random spot urine copper excretion indexes are informative biomarkers for population screening of pre-symptomatic WD among healthy school children.

Aims: With the ultimate goal of defining the screening biomarker of high sensitivity and specificity and the optimal screening age, our study will provide the feasibility and background data to support a large scale study. Copper and zinc concentration were analyzed in 76 school age children of age between 5 to 10 years old. by inductively-coupled-plasma-mass-spectrometry. In addition, urine creatinine and osmolality are also measured. Various spot urine indexes will be calculated and analysed with Receiver-operator-curve analysis.

Results and conclusion: Random Spot urine copper concentration showed a fairly good correlation with spot urine creatinine concentration among controls (Pearson *r* = 0.7, *p* < 0.05). It indicates that spot urine copper/creatinine ratio should be an informative biomarker in children of this age group. Both random spot urine copper concentration and the copper to creatinine ratio can easily differentiate pediatric Wilson patients and healthy controls. ROC analysis showed area under curve value of 0.98 and over 98% sensitivity. Our latest preliminary data suggested that spot urine copper excretion index could differentiate WD from healthy children.

### P109. Reliability of a Multiplex qPCR Assay for the Newborn Screening of SCID, SMA and XLA

Gutierrez-MateoCristinaFilippovGalinaRichardsJoel W.DallaireStephanieGoldfarbDavidWuRongcongPerkinElmer, Molecular Diagnostics, Waltham, MA, USA

The Recommended Uniform Screening Panel (RUSP) is a list of disorders that are recommended by the Secretary of the Department of Health and Human Services (HHS) for states to screen as part of their state universal newborn screening (NBS) programs in the US. Disorders on the RUSP are chosen based on evidence that supports the potential net benefit of screening, the ability of states to screen for the disorder and the availability of effective treatments. In 2010, SCID screening was added to the RUSP allowing affected infants to be identified and receive treatment sooner. Similarly, evidence supporting the benefits of universal newborn screening for SMA has been reviewed and the condition was added to the RUSP in July 2018.

We have developed a four-plex, real-time PCR assay to screen for SCID, XLA and SMA in DNA extracted from a single 3.2mm punch of a dried blood spot (DBS). A simple, high-throughput, buffer DNA extraction method was developed for a Janus liquid handler that can process 384 DBS punches in four 96-well plates in just over one hour. The PCR assay identifies the absence of exon 7 in the SMN1 gene while simultaneously evaluating the copy number of T-cell receptor excision circles (TREC) and Kappa-deleting recombination excision circles (KREC) molecules. Additionally, the amplification of a reference gene, RPP30, was included in the assay as a quality/quantity indicator of DNA isolated from the DBS.

The assay performance was demonstrated on over 1000 DNA samples isolated from punches of putative normal newborn DBS. The reliability and analytical accuracy was further evaluated using DBS control and contrived positive samples. The results from this study demonstrate the potential of future molecular DBS assays and highlight how a multiplex assay could benefit newborn screening programs.

### P110. A demonstration of the Robustness of a 4-Plex qPCR Assay that Detects SMN1, TREC, KREC and RPP30

DallaireStephanieGutierrez-MateoCristinaFilippovGalinaRichardsJoelGoldfarbDavidWuRongcongPerkinElmer, Molecular Diagnostics R&D, Waltham, MA, USA

A four-plex, real-time PCR assay was developed to effectively identify the homozygous deletion of exon 7 in the SMN1 gene and simultaneously evaluate the copy number of T-cell receptor excision circles (TREC), Kappa-deleting recombination excision circles (KREC) in the DNA extracted from a 3.2 mm newborn DBS punch. A simple buffer DNA extraction method was developed to get high quality DNA out of this DBS punch. Lack of TREC and KREC molecules in the blood and the homozygous deletion of SMN1 exon 7 are widely-accepted biomarkers of SCID, XLA and SMA for newborn screening.

To evaluate the effectiveness of the assay, we compared both the DNA extraction and the PCR mix to comparative methods that are being used to test for SCID, XLA and SMA in newborn screening. More than 1000 newborn samples were extracted using the simple buffer DNA extraction as well as using an in-situ method of extraction. For the in-situ method, an in-house elution solution was compared to the commonly used Extracta DBS solution using our multi-plex assay.

The PCR assay is comprised of two reagents: one containing a reaction buffer and enzyme and one with the primers and the probes for the multiplex assay. In the second study, we used the simple buffer DNA extraction material and swapped the reaction buffer with Perfecta ToughMix^®^ which is commonly used for SCID testing.

The results of these tests suggest that the in-situ assay is compatible with the PCR chemistry developed to identify the homozygous deletion of exon 7 in the SMN1 gene and simultaneously evaluate the copy number of TREC and KREC using a simple elution solution or Extracta DBS solution. The results also confirm that the PCR System developed is optimized fully to detect the four targets in the qPCR assay as compared to Perfecta ToughMix^®^.

### P111. Newborn Screening Facilitates Early Diagnosis of Spinal Muscular AtrophyNewborn Screening Facilitates Early Diagnosis of Spinal Muscular Atrophy

LiuMei-YingChenHsiao-JanWangLi-YunChouYun-HuiChiangSzu-HuiKaoShu-MinHoHui-ChenHsiaoKwang-JenJongYuh-JyhThe Chinese Foundation of Health, Newborn Screening Center, Taipei, Taiwan

Background: Spinal muscular atrophy (SMA) is an autosomal recessive disease characterized by defect in the survival of motor neuron 1 (*SMN1*) gene. Type 1 SMA patients typically develop symptoms within the first six months after birth and most patients die before age 2 years. Therefore, early detection by SMA newborn screening (NBS) provides neonates the opportunity for early SMA diagnosis and standard care. We report our results of 18-month period of SMA NBS.

Methods: Two NBS centers, Taipei Institute of Pathology (TIP) and The Chinese Foundation of Health (CFOH), screened for SMA using dried blood spots between September 2017 to February 2019. TIP detected the homozygous SMN1 deletion by genotyping 4 polymorphisms between the SMN1 and SMN2, including the c.840C/T alleles, using Sequenom MassArray. CFOH performed a real-time polymerase chain reaction (RT-PCR) assay to detect the presence of the c.888+100A in the SMN1 intron 7. Molecular diagnosis of neonates with SMN1-deleted status was confirmed by multiplex ligation-dependent probe amplification (MLPA) using genomic DNA samples.

Results: of the 164,352 screened newborns, 10 and 12 newborns were screened positive by RT-PCR and Mass spectrometry, respectively. Thirteen cases with the absence of SMN1 were confirmed by MLPA and then all remained asymptomatic. During 6-month follow-up, five of them with 2 copies of SMN2 became symptomatic and diagnosed as type I SMA, 2 of then died of necrotizing enterocolitis and acute respiratory failure, respectively. While six cases with 3 or 4 copies of SMN2 remained asymptomatic, another 2 lost follow up. The other 9 cases revealed 1 copy of SMN1 by MLPA and eight of the nine false-positive cases were resulted from gene conversions between SMN1 and SMN2.

Conclusion: SMA NBS by RT-PCR and Mass spectrometry is feasible and can provide early diagnosis of SMA. Our results revealed an incidence of newborns with homozygous SMN1 deletion in Taiwan is about 1 in 12,642.

### P112. Newborn Screening of Congenital Toxoplasmosis Infection in northern Taiwan

ChenHsiao-JanChungYing-HsuanWenKai-TingLiaoHsuan-ChiehLiuMei-YingKaoShu-MinHsiaoKwang-JenTsaoPei-ChenThe Chinese Foundation of Health, Newborn Screening Center, Taipei, Taiwan

Background Congenital Toxoplasmosis is a cause leading to development of central nervous system anomalies and neurological sequelae in neonates if their mother acquired infection during pregnancy. Early detection and accurate management can reduce the burden from these long-term neurological sequelae. This study aimed to investigate the seropositive rate of Toxoplasma infection in pregnant women and for early diagnosis and treatment of congenital Toxoplasmosis in newborns.

Methods Dried blood spots (DBS) were collected for detection of Toxoplasma-specific IgG and IgM using the ELISA and FEIA, respectively. Newborns with any of positive results were referred for second test within one month. Newborns with seroconversion to positive IgM in the second test or with two positive results of IgM in the first and second tests were identified as congenital Toxoplasmosis. The confirmed newborns were suggested for follow-up of auditory function and neurodevelopment for 3 years.

Results of the 723 neonates in northern Taiwan using DBS screened for Toxoplasma from March to December 2018, the seropositive rate of Toxoplasma IgG was 4.98%. In total, none of these seropositive infants had increased the titer of IgG and changed to positive titer of IgM during regular follow-up. Among the seropositive cases, one of these participants’ mother was seropositive for both IgG and IgM and a subsequent IgG avidity test showed high avidity index for IgG antibodies which indicated that the mother got new infection during early pregnancy.

Conclusion Congenital toxoplasmosis has negative impact on not only neurological outcome of the infected newborns but also on public health system. Newborn screening of Toxoplasma and regular follow-up will improve the neurodevelopmental outcomes due to early detection and treatment.

### P113. Newborn Screening of Congenital Cytomegalovirus Infection in Northern Taiwan

ChenHsiao-JanChungYing-HsuanWenKai-TingLiaoHsuan-ChiehLiuMei-YingKaoShu-MinHsiaoKwang-JenTsaoPei-ChenThe Chinese Foundation of Health, Newborn Screening Center, Taipei, Taiwan

Background Cytomegalovirus (CMV) is the most common congenital infection in human. The incidences of congenital CMV infection–related progressive hearing loss and neurodevelopmental impairment are underestimated due to unrecognition of most infected newborns lacking of clinical manifestations at birth. This study aimed to early detection of congenital CMV infection and set up a follow-up protocol to investigate the incidences of neurological sequelae.

Methods Dried blood spots (DBS) were screened for human CMV-specific IgM. The presence of DNA fragments of CMV in dried saliva swabs was detected using qPCR. Swabs were collected at least 1 hour after breast milk feeding. Newborns with any of positive results were referred to confirm using urine CMV PCR or cultures. The confirmed newborns were suggested for follow-up of auditory function and neurodevelopment evaluation for 3 years.

Results of the 723 newborns in northern Taiwan from March to December 2018, six has positive results. There were 83 cases born prematurely and three of them had positive results. of positive cases, five newborns was reconfirmed congenital CMV infection. The incidence of congenital CMV infection is 0.7%. All these cases passed their hearing screening exams. Until Dec. 2018, four confirmed cases had complete their follow-up exams at the age of 6 months old. Two infants still had positive results of urine CMV culture and no case had the diagnosis of hearing defect or neurodevelopment impairment.

Conclusion Saliva qPCR is a feasible approach for screening of congenital CMV infection. We expect that a follow-up protocol could provide the epidemiology data of late-onset neurological sequelae in children with asymptomatic CMV infection. Furthermore, early detection and intervention may improve the outcomes of these children.

### P114. Screening of Inborn Genetic Disorders X-ALD, ADA-SCID, ASA-LD and OTCD with Specific New Analytes Included in the NeoBase™ 2 Non-Derivatized MSMS Kit

AppelblomHeidiLehtonenTeroBrozinskiJenny-MariaChristineDorleyMcKinlayMargoPölönenTuukkaKoivuAkiVaahteraKatjaPerkinElmer, Diagnostics, Turku, Finland

In total 57 analytes can be screened with the CE marked next generation NeoBase™ 2 Non-derivatized MSMS kit, providing more conclusive screening results and possibility to screen more inborn errors of metabolism when compared to the previous non-derivatized MSMS kit, NeoBase™.

New analytes include very long-chain (C20-C26) lysophospholipids and acylcarnitines, which levels reflect the abnormal very long-chain fatty acid (VLCFA) profiles in the X-linked adrenoleukodystrophy (X-ALD) patients. X-ALD occurs, when mutated ABCD1 gene encodes defected adrenoleukodystrophy protein (ALDP) involved in VLCFA transport and thus leading to VLCFA accumulation in body. Specific markers for Adenosine deaminase (ADA), adenosine and 2-deoxyadenosine, have been included in order to effectively screen ADA patients. Deficient ADA enzyme has reduced ability to convert deoxyadenosine to non-toxic metabolites causing eventually severe combined immunodeficiency (SCID). 10–15% of all SCID cases have ADA deficiency, which onset may be delayed. Additional specific markers, Argininosuccinic acid (ASA), Glutamine (Gln) and Glutamic acid (Glu) were included to improve screening performance of two of the most common Urea cycle disorders, Argininosuccinic acid lyase deficiency (ASA-LD) and Ornithine transcarbamylase deficiency (OTCD).

Two external studies were conducted in US. In both studies approximately 2000 newborn specimens were tested for population distribution and 2500 specimens to demonstrate screening performance including 4 X-ALD, 4 ADA-SCID, 3 ASA-LD and 3 OTCD confirmed positive specimens. All listed specimens were identified or screening performance was shown to be improved when NeoBase™ 2 assay with new analytes was used.

### P115. Clinical Performance of the GSP^®^ Neonatal Creatine Kinase—MM kit for Duchenne Muscular Dystrophy (DMD) Newborn Screening in the US and European Population

PolariHannaHougaardDavidBakerMeiKorpimäkiTeemuPölönenTuukkaMeriöLiisaSkogstrandKristinAirenneSariLloyd-PuryearMicheleKennedyAnnieVershaveLisaTimonenAnnePerkinElmer, Diagnostics, Turku, Finland

Duchenne muscular dystrophy (DMD) is the most common muscular dystrophy among children affecting mostly males (1:5000) and is caused by mutations in the dystrophin gene. With DMD, muscle specific creatine kinase isozyme CK-MM is released into the bloodstream enabling screening for DMD with this biomarker. GSP^®^ Neonatal Creatine Kinase –MM kit is a new fully automated assay based on DELFIA^®^ technology for the GSP platform. The assay is the first IVD kit bearing CE mark for DMD newborn screening.

Three clinical performance studies were conducted to produce newborn CK-MM distribution data in the Danish and US population using the GSP CK-MM assay and evaluate the assay’s ability to distinguish newborns at an increased risk for DMD from the normal population.

The study specimens included 5145 de-identified residual routine newborn screening dried blood spot samples from US and Europe. To enrich the study populations, 65 confirmed positive samples from children that had been collected from DMD affected newborns were also analysed. The samples were obtained from the newborn screening laboratory in the US, the Danish Newborn Screening Biobank and from California Biobank Program.

With 99.5th percentile cut-off, all DMD positive specimens except for one, which was from a very preterm (27 GA) and very low birth weight (1200 g) newborn, were classified as screen positive. Across the three studies specificity was 99.6%, sensitivity 98.6%, positive predictive value 4.6% and negative predictive value 100%.

The studies demonstrated good performance of the GSP^®^ Neonatal Creatine Kinase –MM kit and the assay discriminated well between normal population and affected DMD cases. The device appears to be well suitable for first tier testing in newborn screening for DMD.

### P116. Newborn Screening for X-Linked Adrenoleukodystrophy by LC-MS/MS

ChenHsiao-JanHuangYi-HuiLiuMei-YingKaoShu-MinThe Chinese Foundation of Health, Newborn Screening Center, Taipei, Taiwan

Background: X-linked adrenoleukodystrophy (X-ALD) is a rare inherited metabolic disorder caused by mutations in ABCD1 gene. The defects result in accumulation of C26:0-lysophosphatidylcholine (C26:0-LPC). Newborn screening of X-ALD has been suggested to add to the recommended uniform screening panel (RUSP).

Method: Analysis of C26:0-LPC in dried-blood spot (DBS) was performed using LC-MS/MS. Suspicious cases were picked against the cutoff values (0.20 μmole/L) and recalled for a second DBS. For the second specimen, the individuals who had C26:0-LPC ranged from 0.20 to 0.99 μmole/L were requested a third DBS and the individuals who had C26:0-LPC equal to or higher than 1.00 μmole/L were referred to a confirmatory hospital for further confirmation diagnosis and following up. If the third specimen still has an amount of C26:0-LPC higher than the cutoff value, the individuals were referred to a confirmatory hospital as well.

Result: Analysis of C26:0-LPC using LC-MS/MS reduced potential interference from other lipids. Total run time was 2.5 min per sample. C26:0-LPC in normal newborns was 0.08±0.03 (mean±SD) μmole/L. From March 2018 to December 2018, 40,902 newborns have been screened for X-ALD. A total of 11 newborns were recalled for second DBS and 1 male newborn who had 0.62 μmole/L C26:0-LPC was referred to a confirmatory hospital and confirmed as an X-ALD case.

Conclusion: Instead of two-tiered screening approach (first tier: flow-injection analysis MS/MS; second tier: LC-MS/MS) for detection of C26:0-LPC, we use one-tiered approach (LC-MS/MS directly screening method), which reduced the screening turnaround time. Our preliminary results reveal the incidence of X-ALD was about 1 in 20,000 male newborns.

### P117. A Demonstration of the Robustness of a 4-Plex qPCR assay that Detects SMN1, TREC, KREC and RPP30

DallaireStephaniePerkinElmer, Molecular Diagnostics R&D, Waltham, USA

A four-plex, real-time PCR assay was developed to effectively identify the homozygous deletion of exon 7 in the SMN1 gene and simultaneously evaluate the copy number of T-cell receptor excision circles (TREC), Kappa-deleting recombination excision circles (KREC) in the DNA extracted from a 3.2 mm newborn DBS punch. A simple buffer DNA extraction method was developed to get high quality DNA out of this DBS punch. Lack of TREC and KREC molecules in the blood and the homozygous deletion of SMN1 exon 7 are widely-accepted biomarkers of SCID, XLA and SMA for newborn screening.

To evaluate the effectiveness of the assay, we compared both the DNA extraction and the PCR mix to comparative methods that are being used to test for SCID, XLA and SMA in newborn screening. More than 1000 newborn samples were extracted using the simple buffer DNA extraction as well as using an in-situ method of extraction. For the in-situ method, an in-house elution solution was compared to the commonly used Extracta DBS solution using our multi-plex assay.

The PCR assay is comprised of two reagents: one containing a reaction buffer and enzyme and one with the primers and the probes for the multiplex assay. In the second study, we used the simple buffer DNA extraction material and swapped the reaction buffer with Perfecta ToughMix^®^ which is commonly used for SCID testing.

The results of these tests suggest that the in-situ assay is compatible with the PCR chemistry developed to identify the homozygous deletion of exon 7 in the SMN1 gene and simultaneously evaluate the copy number of TREC and KREC using a simple elution solution or Extracta DBS solution. The results also confirm that the PCR System developed is optimized fully to detect the four targets in the qPCR assay as compared to Perfecta ToughMix^®^.

### P118. Development of Hear Loss Genetic Screening in Newborn Using MALDI TOF Assay

WangLi-yunHoHui-chenChangShu-MingLinChien-HsingTaipei institute of pathology, Clinical pathology, Taipei city, Taiwan

The feasibility of hear loss genetic screening is well recognized to finding additional subgroup of infant with late-onset hearing impairment that might not be missed by newborn hearing screening (NHS) program. Here we establish 22 mutations screening method within GJB2, GJB3, SLC26A4, mitochondrial 12S rRNA, OTOF genes using MALDI TOF assay. DNA is extracted from dry blood spot (DBS) and processed in one reaction well. Agena’s MassARRAY system is used to detect the reaction product and the genotype of each mutation is acquired using TYPER software. The assay validation was complete using synthetic double stranded gene fragments carrying mutation and DBS from genotype conclusive infants. All of the mutations are correctly determined. In the pilot study to evaluate mutation carrier rate, 195 cases of samples are screened. 31 case of GJB2(15.8%), 2 cases of SLC26A4(1.02%) and 3 cases of OTOF (1.53%) are identified. Comparing the carrier frequency of 1000 genome EAS, all of the mutations carrier rate higher than 0.5% are seen in the study. The workflow is established within automatic liquid handling system using 384 PCR plate format which make the sample throughput up thousands per week. This method is promising for newborn screening lab offering low labor and time cost for multiple genetic marker screening.

### P119. Evaluation of a Suitable CK-MM Assay in Newborn Screening for Duchenne Muscular Dystrophy

BakerMeiLoeheMandieKlockziemKellyLasarevMichaelUniversity of Wisconsin School of Medicine and Public Health, Wisconsin State Laboratory of Hygiene, Madison, WI, USA

Duchenne muscular dystrophy (DMD) is an X-linked disorder characterized by rapid progression of muscle degeneration that occurs early in life. The incidence rate is estimated at 1 in 3500 boys worldwide. The effective emerging treatment methods and strategies have resulted in growing interest and discussion regarding newborn screening (NBS) for DMD.

It has long been recognized that creatine kinase (CK) enzyme activities are increased in asymptomatic boys with DMD. A recently reported study has indicated that CK-MM, one of three isoenzyme forms of CK and found predominantly in skeletal muscle, can be reliably measured in dried blood samples and used for DMD NBS. Our study further evaluated the performance of the reported automated immunoassay for CK-MM. We have assessed the assay accuracy, precision and linearity. After obtaining the results that met clinical testing acceptance criteria, we have further assessed the CK-MM distribution in newborns using 9224 de-identified residual dried blood NBS specimens collected within 24-120 hours after birth. Except one male newborn’s data that has a CK-MM concentration at 14, 674 ng/mL, the CK-MM assay data were analyzed for the overall distribution and distribution by sex, age at specimen collection, gestational age and birth weight. In the overall distribution data set, the median CK-MM concentration is 355 ng/mL and the CK-MM concentration at 99.5th percentile is 2168 ng/mL (90% CI: 2020–1310 ng/mL). There were 46 samples at 99.5th percentile with CK-MM concentration ranging from 2179–6923 ng/mL.

We conclude that CK-MM appears to have the required accuracy and precision for clinical screening for DMD when used as the first tier and followed by DMD gene mutation analysis; CK-MM distribution in male newborns and female newborns have a similar pattern; CK-MM concentrations in newborns appear to be associated with age at specimen collection, gestational age and birth weight.

### P120. Metabolic Pathway and Diagnostic Markers Analysis of Congenital Heart Disease Based on Metabolomics

LiYahongSunYunZhangXiaojuanWangYanMengLuluZhouRanZhouXiaoyanLuoChunyuHuPingJiangTaoXuZhengfengWomen’s Hospital of Nanjing Medical University, Center of Prenatal Diagnosis, Nanjing, China

Congenital heart disease (CHD) is the most common birth defect with 6‰～11‰ incidence rate worldwide. However, the current understanding of the relationship between the metabolite profiles of amniotic fluid and fetal CHD during pregnancy is extremely limited. Global metabolite profiling was performed on amniotic fluid samples from the CHD and the control group. A third group of other abnormal pregnancy outcomes or other birth defects (OBDs) was used to validate the metabolite markers associated with fetal CHD. Compared with the control group, several important metabolic pathways in the CHD group, including alanine, aspartate and glutamate metabolism; valine, leucine and isoleucine biosynthesis; arginine biosynthesis; glutamine and glutamate metabolism; glutathione metabolism; and phenylalanine, tyrosine and tryptophan biosynthesis, was disrupted. Four metabolite markers, that is, the ratio of glutamic acid/pyroglutamic acid, glutamic acid, the ratio of glutamic acid/oxoglutaric acid and the ratio of oxoglutaric acid/glutamic acid, which were significantly different between the CHD and the control group, were selected for CHD diagnosis, thereby producing a good diagnostic efficacy by using ROC curve. Another group of various mixed OBDs demonstrated more dysregulated metabolic profiles than those present in CHD. However, the four CHD diagnostic markers were specific because of the insignificant change among these markers in the OBD group. In this study, disturbed pathways and diagnostic markers of CHD were revealed. These findings improved our understanding of the physiopathological mechanism of CHD, thereby leading to considerable potential for CHD diagnosis.

### P121. Comparison of C26:0-Carnitine and C26:0-Lysophosphatidylcholine as Diagnostic Markers of Chinese X-Linked Adrenoleukodystrophy

TianGuoliXuFengWangYanminJiWeiShanghai Children’s Hospital, Neonatal Screening Center, Shanghai, China

X-linked adrenoleukodystrophy (X-ALD) is a progressive neurodegenerative disease caused by mutations in the ABCD1 gene which results in the accumulation of very long-chain fatty acids (VLCFA) in tissues of the body. Early diagnosis of patients with X-ALD is essential and it can lead to life-saving interventions. Two different VLCFA-markers, C26:0-carnitine and C26:0-lysophosphatidylcholine (C26:0-LPC), have been identified as diagnostic molecules for X-ALD in dried blood spots (DBS) from newborns and patients. However, the application of them in Chinese DBS is unclear. Therefore, we evaluated the feasibility of very long chain acylcarnitines and lysophosphatidylcholines for Chinese patients of X-ALD.

We collected DBS cards from 7 boys with X-ALD, 114 age-matched control and 3063 putative normal newborns and then we extracted 1/8-inch DBS punches with 125 μl extraction solution containing D3-C26:0-carnitine and D4-C26:0-lysoPC stable isotope-labeled internal standards. The extractions were transferred to 96-well plates for tandem mass spectrometry (MS/MS) analysis.

The concentrations of C24:0-carnitine, C26:0-carnitine, C24:0-LPC and C26:0-LPC were elevated in analyzed DBS samples of 7 patients with X-ALD. However, only C26:0-carnitine can clearly differentiate patients from newborn controls. The distribution of C26:0-LPC concentration in Chinese normal newborns is wider so that it overlaps with the corresponding concentration of patients. Similarly, C26:0/C22:0-carnitine has better performance than C26:0/C22:0-LPC to distinguish patients with X-ALD from controls. We recommend that the inclusion of C26:0-carnitine detection into the routine analysis of acylcarnitines and amino acids for effective screening for X-ALD in China.

J. Genomics

### P122. Clinical Application of Chromosome Microarray Analysis in a Case of Sex Chromosome Abnormality

ZhuLingYangRulaiWuDingwenThe Children’s Hospital Zhejiang University School of Medicine, Department of Genetics and Metabolism, Hangzhou, China

Objective: Chromosomal Microarray Analysis (CMA) can simultaneously detect chromosome number and structure abnormalities. The purpose of this study was to investigate the clinical application of CMA in a patient with sex chromosome abnormality.

Methods: A case of sex chromosome abnormality was detected and analyzed by SNP genome-wide chromosome microarray analysis (Affymetrix CytoScan HD chip). On the basis of chromosome karyotype analysis, CMA technology was used for further analysis to make a definite diagnosis. The samples were peripheral blood of patients and the results were analyzed by ChaS software. Through the retrieval and analysis of ISCA, DECIPHER, UCSC, OMIM, PubMed, ClinVar and other databases, the clinical diagnosis was made clear.

Results: The chromosome karyotypes of the subjects were 46, X, +mar(91)/45, X(9). About 14 Mb genome fragment of Y chromosome deletion detected (because of the probes uncovered the Yq12 region, the actual deletion region of Y chromosome was estimated to be Yq11.21-qter, about 44.4 Mb and the Smooth Signal value of this region was about 0.6, suggesting the possibility of chimeric deletion variation). A number of pathogenicity reports less than or similar to the deletion variants detected in this report were retrieved in ISCA, DECIPHER, ClinVar database. Further searches of OMIM and PUBMED databases showed that Yq11.21q11.23 deletion variants were associated with diseases and their clinical phenotypes were diverse, including spermatogenesis disorders, gonadal dysplasia, lack of secondary sexual characteristics in adolescence and short stature.

Conclusion: For patients with abnormal sex chromosomes, selective CMA detection can help to identify the genetic causes and assist in clinical diagnosis, which is conducive to evaluating the risk of recurrence of the disease in the family.

**Keywords:** chromosome microarray analysis; CMA; sex chromosome

### P123. Pseudodeficiency Alleles Effect the Fluorescence Assay of Acid α-Glucosidase Activity for Newborn Screening of Glycogen Storage Disease type II

ChenTingQiuWenjuanShanghai Institute for Pediatric Research, Department of Pediatric Endocrinology and Genetic, Shanghai, China

Objective To investigate the effect of pseudodeficiency alleles on the fluorescence assay of acid α-glucosidase (GAA) activity in dried blood spot (DBS) for newborn screening of glycogen storage disease typeII (GSDII).

Methods A total of 30507 cases of newborns were screened for GSDII, then the suspected positive newborns were confirmed by gene test to figure out the carrying of GAA gene mutations and pseudo deficiency alleles. A retrospective analysis of 3729 cases of non-GSDII patients and 36 cases of GSDII patients was processed to investigate the carrying of pseudo deficiency alleles. Statistical analysis was carried out by Chi-square test or Fisher exact probability test.

Results The distribution of acid GAA activity of 30507 newborns was positively skewed. 29 cases of newborns were suspected positive after the screening and the median value of acid GAA activity was 3.25 (2.20~4.11) μmol/L/h. Gene test confirmed that none of the 29 cases was GSDII patient. of the 29 cases, 24 cases were homozygous for pseudo deficiency alleles c.[1726A/A; 2065A/A] and the other 5 cases were c.[1726G/A; 2065G/A] heterozygote. The frequency of c.1726G>A homozygote was 2.08%. When c.1726G>A was homozygous, 100% of c.2065G>A was homozygous. Frequency of c.[1726A; 2065A] haplotype was 3.2%. Frequency of diplotype c.[1726A/A; 2065A/A] homozygote in 36 cases of GSDII patients (16.67%) was significantly higher than that in non-GSDII patients (2.08%) (*p* value <0.001).

Conclusions Pseudodeficiency alleles show a high carrier rate in the Mainland China population, which leads to a high false positive rate in the newborn screening of GSDII and further interferes the process of newborn screening procedure.

### P124. Misdiagnosis of Three Boys in One Family with Xp21 Contiguous Gene Deletion Syndrome

LuMeiYangYanlingXiamen Maternal and Child Care Hospital, Department of Pediatrics, Xiamen, Fujian, China

Introduction: Xp21 contiguous gene deletion syndrome is a rare X-linked recessive disorder resulting in glycerol kinase deficiency, congenital adrenal hypoplasia, intellectual disability and/or Duchenne muscular dystrophy. In this study, Xp21 contiguous gene deletion syndrome was confirmed in one family with three boys by clinical and genetic analysis.

Subjects and Methods: The first child of the family was a boy. He showed dark skin and development delay at the age of one month and died at the age of 1 year without definite etiological diagnosis.

The second boy had similar clinical features. Congenital adrenal hyperplasia was suspected. Elevated serum creatine kinase and myohemoglobin were noticed. He was also died aged 1 year because of a febrile illness.

The third boy was hospitalized for dark skin and hypotonia at the age of 7 days with the suspected diagnosis of congenital adrenal hyperplasia but his17-OHP in dried blot spot of newborn screening was normal.

Results: Significantly increased serum creatine kinase and myohemoglobin were found in the third boy of the family, indicating rhabdomyolysis.

Elevated plasma adrenocorticotropic hormone with continuous low 17-OHP, severe hyperkalemia, hypertriglyceridemia and glyceroluria were observed.

Xp21 contiguous gene deletion syndrome was confirmed by a SNP array for the boy. After the supplements of hydrocortisone and fludrocortisone, the patient improved rapidly. But the serum creatine kinase was not decreased.

Conclusions: A family affected by Xp21 contiguous gene deletion syndrome was reported. Three boys experienced misdiagnosis. Although they had the similar syndrome of CAH, no positive results were found in newborn screening.

The differential diagnosis should be paid attention to for the patient with early-onset hypoadrenocorticism.

### P125. PCR Amplicon-Based NGS Panel for Newborn IEM

GuoYongchaoShenzhen Uni-Medica Co., Ltd., Shenzhen Uni-Medica Co., Ltd., Shenzhen, China

Background: Tandem mass spectrometry (MS/MS) is widely used for the newborn screening. However, some genetic diseases with relatively high incidence are not covered by MS/MS platforms and the misdiagnosis rates by MS/MS are relatively high for some diseases. So, the next generation sequencing (NGS) technology has become the alternative technology for clinical diagnosis.

Methods: The "Baby Health" newborn NGS project adopts the “all-in-one” ultrahigh PCR amplicon capture technology independently developed by Uni-Medica to achieve rapid and efficient library construction based on dry blood spots (five 3 mm spots) for down-stream sequencing. The newborn NGS panel contains 140 genes of 135 genetic diseases suitable for neonatal detection, including diseases that are conventionally detected by MS/MS and other diseases with screening value for neonates, for example, lysosomal diseases, Wilson disease and so on. In addition, the panel also covers metabolic epilepsy such as pyridoxine-dependent epilepsy, serine synthesis disorder, creatine synthesis disorder, which can be used to explore more possibilities for newborn screening.

Results: The exons coverage of the newborn NGS panel is greater than 98%, while the coverage of pathogenic sites in public databases (ClinVar, HGMD) is 99.9%. The accuracy rate was 99.8% based on the results of single blind tests with five hundred samples. So far, the project has analyzed more than ten thousand clinical samples and has accumulated a database with both genetic mutations and clinical features in the Chinese population.

To enable easy analysis of NGS data of the newborn NGS panel, an automated gene analysis system platform (AGAS, http://nbsgenomics.uni-medica.com/home)has been developed. With AGAS, it takes only five minutes to automatically analyze one sample of sequencing data, including report output. The entire process from sample to report can be completed within 36 to 48 hours, which well meets the practical clinical needs.

### P126. The SLC26A4 Gene Mutations in Children with Deafness and Large Vestibular Aqueduct Syndrome

LiJunDaiXiangWuhan Children’s Hospital (Wuhan Maternal and Child Healthcare Hospital), Tongji Medical College, Huazhong University of Science & Technology, Department of Otolaryngology, Wuhan, China

Objective To analysis the SLC26A4 mutations in families associated with large vestibular aqueduct syndrome (LVAS) by genetic testing method, for the purpose of understanding etiology of hearing loss and providing appropriate genetic counseling.

Methods Blood samples and clinical data of 4 families associated with large vestibular aqueduct syndrome and 153 sporadic cases with non-syndromic hearing loss were collected. The SLC26A4 gene of the patients and their family members were amplified by polymerase chain reaction(PCR), then subjected to automatic DNA sequencing.

Results Homozygous mutation SLC26A4 c.919-2A>G was detected in 3 patients from 3 families and heterozygous mutation SLC26A4 c.919-2A>G was detected in their parents. Compound heterozygous mutations SLC26A4 c.919-2A>G and H723R were detected in one patient, heterozygous mutation c.919-2 was detected in the father, heterozygous mutation H723R was detected in the mother. In 153 sporadic cases, homozygous mutation SLC26A4 c.919-2A>G was detected in 7 patients and compound heterozygous mutations SLC26A4 c.919-2A>G and H723R were detected in 2 patients.

Conclusion Homozygous mutation SLC26A4 c.919-2A>G and compound heterozygous mutations SLC26A4 c.919-2A>G/H723R are the most common causes of hearing loss with enlargement of the vestibular aqueduct. The screening for the SLC26A4 gene mutations is useful in the clinical diagnosis of LVAS.

### P127. Phenotypic and Genetic Analysis of a Child Featuring Multiple Malformations Due to 10p13p15.3 Duplication

XuXiaochaZhangTingShenYapingZhengJingYangRulaiThe Children’s Hospital Zhejiang University School of Medicine, Genetic Metabolism, Hangzhou, China

Objective: To analyze a child with multiple malformations and the correlation of phenotype-karyotype.

Methods: The karyotype of the child was analyzed with G-banding chromosome analysis and Low-coverage massively parallel CNV sequencing (CNV-seq) was used to definite the aberrant region. The child and his parents were verified by Fluorescence in situ hybridization (FISH).

Results: The karyotype was 46, XX, 3pter by G-banding. CNV-seq has identified a 13.5 Mb duplication at 10p13p15.3(60.466-13.556.655) and a 636 kb microdeletion at 3p26.3 (60.064-695.821), the karyotype is specified as 46, XX, ish der (3) t (3:10) (10p+, 3pdim) by FISH, the parents were normal, indicating the de novo origin of the variant in the proband.

Conclusion: The de novo 10p13p15.3 duplication in this child underlies the clinical malformations such as mental retardation, development delay and dysmorphosis, gastroesophageal reflux. The CHL1 gene at 3p26.3 is playing an important role in the building and functioning on the brain and maybe overlap with the child’s intellectual deficit.

### P128. A Novel SPECC1L Mutation Causing Teebi Hypertelorism Syndrome: Expanding Phenotypic and Genetic Spectrum

ZhangTingHuangXinwenThe Children’s Hospital Zhejiang University School of Medicine, Department of Genetics and Metabolism, Hangzhou, China

Only five SPECC1L mutations have been reported worldwide which were associated with autosomal dominant oblique facial clefts, Opitz G/BBB Syndrome and Teebihypertelorism syndrome. In this study, we reported the first Chinese patient with Teebihypertelorism syndrome. Utilizing whole exome sequencing and Sanger sequencing, we identified ade novo missense mutation in SPECC1L gene. With common manifestations in Teebihypertelorism syndrome such as special facial appearance, umbilical malformations and congenital heart defects, the patient also had unusual symptoms including recurrent infections, febrile seizures and widely opened anterior fontanelle. We analyzed all the SPECC1L mutations reported so far byI-TASSER and COILS program. Positions e and g in Coiled-coil domain 2 of SPECC1Lwas most frequently mutated and proline seemed to be the predominantly substituted residue. A broader phenotype of Teebihypertelorism syndrome was expanded and molecular characteristics of SPECC1L mutations were firstly elaborated.

### P129. Two Novel Mutations of Ornithine Transcarbamylase Gene Identified from Three Chinese Ornithine Transcarbamylase Deficiency Patients

ShenYapingZhengJingThe Children’s Hospital Zhejiang University School of Medicine, Department of Genetics and Metabolism, Hangzhou, China

We aim to analyze the clinical symptoms, blood metabolic profiling and the gene mutation of ornithine transcarbamylase (OTC) in three ornithine transcarbamylase deficiency (OTCD) patients. The profiling of amino acids and acylcarnitine was determined using MS-MS assay. The OTC exons were amplified using PCR amplification. DNA sequencing was performed, based on which mutation analysis of OTC genes was carried out. For the clinical symptoms, case 1 was a male patient with blood ammonia 1600–1700 μmol/L, lactate urine, serum citrulline concentration decreased significantly and died 5 days after birth. Case 2 was a male patient with citrulline decreased and blood ammonia increased in neonatal genetic metabolic disease screening. Case 3 was a 4Y1M female patient with character changes, stuttering and walking instability, high blood ammonia. The gene mutation result was case 1, a c.870delT was identified in exon 9, which is a frameshift mutation. In case 2, a c.931G>A substitution was identified in exon 9, leading to replacement of valine by methionine in codon 311. In case 3, c.566T>C mutation was noted in exon 6, resulting in missense mutation of leucine to proline in codon189. c.870delT and c.566T>C are novel and the c.931G>A is de novo. So, from our study, the OTC genetic diseases spectrum is expanded.

### P130. A De Novo Heterozygous Frameshift Mutation Identified in BCL11B Causes Neurodevelopmental Disorder by Whole Exome Sequencing

QiaoFengchangHuPingXuZhengfengNanjing Maternity and Child Health Hospital, Prenatal Diagnosis, Nanjing, China

Next-generation sequencing has been invaluable to delineate the genetic etiology of neurodevelopmental disorders in recent years. BCL11B, encoding Cys2His2 zinc finger transcription factor, is essential for the development of immune and neural systems. Herein we describe a Chinese girl presenting craniofacial abnormalities, developmental delay and intellectual disability with speech impairment. Using whole exome sequencing, we identified a de novo heterozygous 11bp frameshift mutation (c.2190_2200delGGACGCACGAC [p.Thr730Thrfs*151]) in exon 4 of BCL11B (NM_138576), which is expected to escape nonsense-mediated mRNA decay and probably result in a truncated protein with lack of the C-terminal DNA-binding zinc-finger domains. This is the first report of non-syndromic neurodevelopmental disorder caused by a BCL11B variant in a Chinese population. The mutation identified in this report broadens the knowledge of mutation spectrum of BCL11B and might help in clinical management, genetic counseling and reducing reproductive risk.

This work was supported by the National Natural Science Foundation of China (81602300, 81770236, 81801445, 81701427), National Key R&D Program of China (2018YFC1002402), Natural Science Foundation of Jiangsu Province (BK20160139, BK20181121) and the Project on Maternal and child health Talents of Jiangsu Province (FRC201791).

### P131. GNPTAB Mutation Causes Mucolipidosis II: A Chinese Case Report

QiaoFengchangHuPingXuZhengfengNanjing maternity and child health hospital, Prenatal Diagnosis, Nanjing, China

Mucolipidoses II is a lysosomal disorder in which the essential mannose 6-phosphate recognition marker is not synthesized on to lysosomal hydrolases and other glycoproteins. The disorder is caused by mutations in GNPTAB, which encodes two of three subunits of the heterohexameric enzyme, *N*-acetylglucosamine-1-phosphotransferase. Herein we described a Chinese boy presenting coarse face, large joints contractures, kyphosis, clubfeet, restricted range of motion in the shoulders and cardiac involvement. The affected boy and his parents were detected by exome sequencing. A homozygous mutation c.3091C>T (p.Arg1031X, 226) in exon15 of GNPTAB was found in the proband inherited from the parents respectively. Our results both extended the knowledge of mutation spectrum in mucolipidoses II patients and highlighted the clinical application of exome sequencing.

This work was supported by the National Natural Science Foundation of China (81602300, 81770236, 81801445, 81701427), National Key R&D Program of China (2018YFC1002402), Natural Science Foundation of Jiangsu Province (BK20160139, BK20181121) and the Project on Maternal and child health Talents of Jiangsu Province (FRC201791).

### P132. Functional Characterization of Argininosuccinate Lyase Gene Variants by Mini-Gene Splicing Assay

WangYanyunNanjing Maternity and Child Health Care Hospital, Center for Genetic Medicine, Nanjing, China

Objective: Argininosuccinate lyase (ASL) gene mutations account for argininosuccinic aciduria (ASA). This study aimed to design a minigene construct of ASL gene in order to investigate the impact of variants on splicing.

Methods: The peripheral blood samples were collected from the family members and genomic DNA was extracted for gene diagnosis using the total exon sequencing method. The novel mutation gene was cloned into pEGFP-C1 vector and the pathogenicity of the mutation was examined in cultured cells in vitro.

Results: The clinical diagnosis of the proband as ASA was clear. Two pathogenic mutations, c.281G>T (p.Arg94Leu) and c.208-15 T>A were detected in the ASL gene and the two mutations had not been reported. The minigene expression in vitro confirmed that c.208-15 T>A could cause aberrant splicing, resulting in the retention of 13 bp in intron 2 to exon 3.

Conclusion: Two new pathogenic mutations of ASL gene, c.208-15 T>A and c.281G>T, were found in an ASA family, which enriches the mutational profile of the ASL gene and provides a basis for genetic diagnosis of ASA. Minigenes are optimal approaches to determine whether the intron mutation can cause aberrant splicing.

K. Method Development

### P133. The Clinic Practice to Screen and Diagnose Multiple Inherited Metabolic Diseases in High Risk Patient by Use of GC-MS

JiangJianhuiZengWeihongLiuShuXieXunjieZengWeihongLiuShuXieXunjieJinQunGuangdong Women and Children Hospital, Children Inherited Metabolism and Endocrine Department, Guangdong Neonatal Scree, Guangzhou, China

Metabonomics has been used widely in disease diagnosis and chromatography-mass spectrometry (GC-MS) has been used to examine multiple findings of urine to screen and the diagnose multiple inherited metabolic diseases (IMDs) clinically.

From the March of 2015 to the December of 2018, 132 findings in the urine of 7875 high risk IMDs patient’ urine samples were examined by GC-MS. IMDs were confirmed by come together with MS-MS, NGS analysis clinically.

In this clinic practice, 1–15 abnormal high findings were detected in total 2565 urine samples and 42 IMDs or abnormal findings were confirm diagnosed in total 556 patients. The ratio of IMDs was 556/7875 (7.06%).

The relations of the findings with the IMDs and the Cut-off values of the markers were discussed.

**Keywords:** metabonomics; gas chromatography-mass spectrometry (GCMS), finding marker; inherited metabolic diseases (IMDs), high risk screening; diagnosis

### P134. Rapid UPLC-MS/MS Dried Blood Spot Analysis of Steroid Hormones Using the Xevo TQ-S Micro for Clinical Research

FoleyDominicBrownHeatherCaltonLisaWaters Corporation, Scientific Operations, Wilmslow, UK

Dried Blood Spots (DBS) are an established microsampling technique providing a low-cost approach of collecting, shipping and analyzing samples for clinical research. Ligand-binding assays (LBAs) are the established frontline testing methodologies for DBS samples in steroid hormone analysis. Although rapid, the relatively low analytical specificity of the LBAs may necessitate follow-up, using liquid chromatography—tandem mass spectrometry (LC-MS/MS). Analysis of steroid hormones panels provides greater detail about the underlying enzyme activity, compared with single-analyte LBAs. The challenge is to create a LC-MS/MS methodology which separates interferences, whilst maximizing throughput. Ultra Performance Liquid Chromatography (UPLC™) on the Waters™ ACQUITY UPLC™ combined with CORTECS™ 2.7µm particle columns provides UPLC separations at high linear velocities with minimal loss in column performance.

An LC-MS/MS method for analysis of steroid hormones in DBS samples for clinical research was developed. An automated offline Oasis MAX µElution SPE protocol was employed for the extraction of steroid hormones from two 3mm DBS samples. Chromatographic separation was performed using a CORTECS C18 2.7 µm column coupled to ACQUITY UPLC I-Class with detection on the Xevo TQ-S micro. The method enabled a rapid injection cycle of 2.3 minutes for 17-OHP, androstenedione, cortisol, 11-deoxycortisol and 21-deoxycortisol with baseline resolution of steroid isobars. Calibration lines were linear from 0.5–500 ng/mL for androstenedione and 11-deoxycortisol; and 1.0–500 ng/mL for cortisol, 17-OHP and 21-deoxycortisol with correlation coefficients (r^2^) >0.99 over five occasions. Coefficients of variation (CV) for total precision and repeatability over five occasions at three concentrations were ≤ 9.3% (*n* = 25).

For Research Use Only. Not for use in diagnostic procedures.

### P135. Discriminant Analysis of Two Kinds of Amino Acid Metabolic Disease by Gas Chromatography-Mass Spectrometry Combined with Chemometrics

GengGuoxingFanXinZhengHaiyangLuoJingsiLinCaijuanHuangXiaotaoChenShaokeMaternal and Child Health Hospital of Guangxi, Newborn Screening Center of Guangxi Province, Laboratory of Genetics and Metabolism, Nanning, China

Objective Determination of the related metabolites in 2 kinds of Amino Acid Metabolic Disease (hyperphenylalaninemia and Citrin deficiency) by Gas chromatography-mass spectrometry, in order to discriminate the possibility of disease and enhance the accuracy of diagnostic.

Methods Base on the R Programming Language, The2 kinds of Amino Acid Metabolic Disease prediction model was established by Variance Analysis, Correlation distribution analysis and discriminant analysis. We have developed the discrimination function which are used to discriminate the possibility of disease.

Results The hyperphenylalaninemia and Citrin deficiency were predicted by the discrimination function and the accuracy of training and model validation were 100% and 69% respectively. Meanwhile the discrimination results were forecasted by ROC curve, the values of AUC are 0.99 and 0.78.

Conclusions Gas chromatography-mass spectrometry combined with Chemometrics gas chromatography-mass spectrometry can be used to predict and discriminate inherited metabolic diseases, however, prediction of some diseases requires a combination of multiple indicators to improve predictive power and chemical measurement method can be used to improve chemical analysis experiments process.

### P136. The Use of 2nd Tier Tests in Newborn Screening for the Determination of Metabolites by Tandem Mass Spectrometry to Improve Performance of Newborn Screening for Inborn Error of Metabolism (IEM)

MasEmilieGeraceRosemarieSA Pathology Women’s & Children’s Hospital, Biochemical Genetics, Genetics & Molecular Pathology, Adelaide, Australia

The impact of tandem mass spectrometry (MS/MS) on both routine newborn screening and biochemical genetic testing for metabolic disorders is considerable. Advancement in MS/MS instrument performance has greatly improved the sensitivity requiring minute amounts of material for the detection of metabolites to levels of less than 1nmol/L. This increase in sensitivity and specificity provides the capacity for the determination of additional metabolites as intermediates of biochemical reactions. Metabolites play a very important role in connecting the many different pathways that operate within a cell. The levels of the metabolites are determined by the concentration and the properties of the enzymes and its co-factors as a complex function of many different regulatory processes inside the cell. Thus, the level of metabolites represents integrative information of the cellular function and defines the phenotype of a cell, tissue and organ in response to genetic or environmental changes. The development of a number of 2nd tier MS/MS tests and their implementation into existing screening strategies has reduce the resample and recall rates and the impact of a screening recall on families. To date, these MS/MS 2nd tier assays include the routine determination of methylmalonic acid, 3-hydroxy propionic acid, homocysteine, alloisoleucine succinylacetone and steroids including 17-a-hydoxyprogesterone. In addition, MS/MS has been used to screen for lysosomal storage disorders by measuring both substrates and products in order to determine deficiencies in specific enzyme activities. This is followed by 2nd tier assays for the determination of metabolites such as Globotriaosylsphingosine (lyso-GB3 & lyso-CTH), Sphingomylein-509, Lyso-PC, Sulphatides and Glycosaminoglycans. Using MS/MS (Sciex & QSight) data from the South Australian program we will show how these novel developments have impacted on expanded newborn screening to significantly improve newborn screening testing for IEM.

### P137. A Retrospective Analysis of Inherited Metabolic Diseases by Gas Chromatography-Mass Spectrometry

TianGuoliZhouZhuoGuoJingWangYanminJiWeiShanghai Children’s Hospital, Neonatal Screening Center, Shanghai, China

Objective To explore the application of gas chromatography-mass spectrometry (GC-MS)in the diagnosis of inherited metabolic diseases.

Method It is a retrospective study. The urine of 5778 children with suspected inherited metabolic diseases who were treated in our hospital from June 2014 to May 2018 was collected. After extraction and derivatization,132 metabolites were detected by gas chromatography-mass spectrometry and analyzed by inherited metabolic disease analysis software and combined with other laboratory tests to definite diagnosis.

Result of the 5778 urine samples, 1100 had abnormal metabolites, accounting for 19% of the total samples; A total of 106 patients with 17 diseases were diagnosed, among which 80 patients were diagnosed with abnormal characteristics in urinary and 26 patients were diagnosed with blood tandem mass spectrometry. Among the 80 cases, there was 65 patients with 11 types of organic acid metabolic disorders and 15 cases with 3 amino acid metabolic disorders, including 33 cases of methylmalonic acidemia, 12 cases of hyperphenylalanine acidemia, 7 cases of propionemia, 7 cases of glutaric acidemia type I, 6 cases of urea circulatory disorders, 3 cases of β-ketothiolase,2 cases of ethylmalonic acidemia, maple syrup urine disease, isovaleric acidemia and multiple carboxylase deficiency, 1 cases of tyrosinemia type I,2-hydroxyglutaratemia,3-methylcrotonyl –CoA carboxylase deficiency and 3-hydroxy-3-methylglutaraturia.26 cases were diagnosed by tandem mass spectrometry, including19 cases of Hitlin deficiency, 5 cases of very long-chain acyl -CoA dehydrogenase deficiency and 2 cases of medium-chain acyl -CoA dehydrogenase deficiency.

Conclusion The gas chromatography-mass spectrometry was specific for the abnormality of organic acids and partial amino acid metabolism abnormality. The results of GC-MS were influenced by many factors but has no difference in clinical judgment.

L. Quality Control/Quality Assurance

### P138. A Verification of the Application of the Non-Derivatized Mass Spectrometry Method in Newborns Screening of Metabolic Disorders

ZhuWenbinZhengYulanFujian Provincial Maternity and Children’s Hospital, affiliated hospital of Fujian Medical University, Neonatal Screening Center, Fuzhou, China

Objectives: It is required that the clinical screening of metabolic disorders in newborns meet International Organization for Standardization (ISO) 15189-2012 approval. The new tandem mass spectrometry (MS/MS) based screening system and its companion reagent should be independently authenticated before their implementation in clinical diagnosis laboratories.

Methods: Linearity, stability, accuracy and precision evaluations were carried out to verify the performance of the Waters ACQUITY TQD MS/MS system with the NeoBase™ non-derivatized MS/MS PerkinElmer kit for detecting amino acids and acylcarnitine in newborns with metabolic disorders.

Results: Statistically, the correlation coefficient (R2) of 0.9982~0.9999 indicates good linearity. The measurements at the beginning and end of the reagent storage procedure were taken for stability verification. No significant difference was detected between the two periods. The amino acid exhibited a degree of bias in the range of 0~14.17%, with acylcarnitine’s being was in the range of 0~14.84%; they consequently passed the quality assessment requirements for clinical laboratories of the China National Centre. The amino acids’ within run, between run and day-to-day run precision were 1.19–7.68%, 1.63–5.01% and 4.77–12.48%, respectively, while the total imprecision was 5.55–13.33%. Acylcarnitine’s within run, between run and day-to-day run precision was 1.2–8.43%, 0.19–9.60% and 2.33–10.74%, respectively, while its total imprecision was 6.57–13.99%. The manufacturer declared that the total imprecision of the tests, using Multiple Reaction Monitoring (MRM), should be less than or equal to 25% of the coefficient of variation (CV) for the kit’s high and low quality control levels.

Conclusion: The performance of the non-derivatized MS/MS screening system in detecting the amino acids and acylcarnitines passed the test’s requirements. It was maintained in accordance with the routine clinical chemical detection system.

### P139. Design and Implementation of 16 Quality Indices for Neonatal Screening in Information Management System

ChenYaoZhuWenbinLiuGuanghuaFujian Provincial Maternity and Children’s Hospital, Affiliated Hospital of Fujian Medical University, Neonatal Screening Center, Fuzhou, China

Objective: To explore the realization of 16 quality control indices of neonatal disease screening in neonatal disease management information system and to establish the multi-center management evaluation model under the framework of the whole province.

Method: Based on the network management system for neonatal screening originally used in our province, the quality management module of neonatal disease was developed and applied according to the quality control index. A quantitative evaluation method was established for the quality of all maternal and child health care institutions involved in neonatal disease screening related to neonatal disease screening, specimen collection and recall of suspected cases.

Results: The quality management information was shared by all neonatal screening agencies. Comprehensive evaluation was given to the multi-center quality management measurements. Comprehensive evaluation was performed to quality among different medical institutions. Evaluation of applicability and timeliness of quality in practical work was conducted accordingly. The timeliness of self-improvement in centers and medical institutions were improved.

Conclusion: The application of neonatal disease screening quality management index improved the requirements for neonatal screening management. The quality management module was adopted to conduct dynamic management of the whole management process, which greatly improved the overall management level of neonatal screening.

### P140. Development and Application of Tracking System by Cold Chain Logistics in Management of Neonatal Screening Specimens

MaoHuaqingThe Children’s Hospital Zhejiang University School of Medicine, Department of Genetics and Metabolism, Hangzhou, China

Objective To develop and apply the tracking system throughout cold chain logistics for specimen delivery of neonatal screening, provide safe and timely specimen delivery to neonatal screening center and keep their proper location and preservation on the basis of original version for neonatal screening system.

Methods By mobile Internet processing technology, the information of specimens delivered in the existing management system of neonatal screening was automatically generated by the system after packaging and processing. The whole process of logistics delivery including specimen reception from blood collection, transportation and eventual specimen reception at screening centers was accomplished by scan completion.

Results The false positive rate of screening was decreased. After specimen was collected, its time arriving screening center was shortened from original 8.01 to 5.45 days currently. The safety of specimen delivery was enhanced and safe arrival rate for specimens was reached 100 %. The specimen could be found by information system and currently its location could be traceable, which was different from the past.

Conclusion The tracking system of whole process by cold chain logistics can be used to make more standardized and secure for blood specimen delivery. It improves the screening quality, makes the test results more accurate and reliable and provides the better guarantee for early detection, diagnosis and treatment of children.

### P141. Ensuring Quality Outcomes by Integrating Long Term Care for Confirmed Cases at the Philippine Newborn Screening Program

PadillaCarmencitaAlcausinMelanie LibertyPanolKarenBeltranFrederick DavidJubanNoelUniversity of the Philippines Manila, Newborn Screening Reference Center, National Institutes of Health, Ermita Manila, Philippines

Background: The Implementing Rules and Regulations (Section 13 H-J) of the Philippine Newborn Screening (NBS) Law or Republic Act 9288 of 2004 states that the Department of Health (DOH), being the lead agency, shall ensure: 1) a network of facilities for referral and management of all positive cases and; 2) a harmonized set of protocols for referral and management of the positive cases. This was the basis of the administrative order issued in 2014 outlining the guidelines in setting up of the NBS Continuity Clinics (NBSCCs).

Objectives: This session will present the development of the regional NBSCCs that takes charge of long term care of patients confirmed under the National Comprehensive NBS Program.

Methods: A program audit was conducted at the 10th year to identify areas for improvement of the program. Focused group discussions led to recommendations to DOH. Datasets on patients seen at the NBSCCs were reviewed.

Results and Discussion: The audit identified a major gap in long-term care due to the lack of hospitals with specialists. The NBSCC was defined as a clinic in a government hospital, equipped for continuity of care of confirmed patients. By 2018, there are 14 strategically located NBSCCs that facilitate referral of patients needing consults to subspecialists, either face-to-face or through teleconferencing. The NBSCCs are manned by a pediatrician and a nurse or genetic counselor. As a minimum, the host hospital must have at least a neonatologist, hematologist and nutritionist/dietitian. Access to endocrinologists and geneticists has been primarily through scheduled regional endocrinology medical missions and telegenetics. Despite these strategies, support is needed particularly for managing acute patients. With expanded newborn screening to be fully implemented in May 2019, this lack is further underscored. The proposed creation of Regional Clinical Genetic Centers is necessary to assist the NBSCCs in responding to the acute and long term needs of the patients that will be diagnosed through ENBS.

### P142. The National Newborn Screening Laboratory in Qatar—Continuous Progress

MitriRolaSkrinskaVictorRamaswamyMamathaJoshiRaviTahtamoniMontherAlabedMamoonAbdohGhassanAbdeinLubnaMohammedKhadijaRamaKahlil>Hamad Medical Corporation, Laboratory Medicine and Pathology, Doha, Qatar

More than 28,000 newborns in Qatar are tested annually for inborn errors of metabolism. The Metabolic Laboratory at Hamad Medical Corporation serves as the National Neonatal Screening Laboratory for Qatar. The Laboratory screens all newborns in Qatar for 83 disorders. The screening panel includes amino acid, fatty acid oxidation and organic acid disorders by liquid chromatography/tandem mass spectrometry (TMS); biotinidase, galactosemia and endocrine disorders by PerkinElmer Genetic Screening Processor; and hemoglobin by Bio-Rad Variant nbs System. Due to the prevalence of homocystinuria, all newborns are also screened for homocysteine. Second tier tests for methylmalonic, methylcitric and ethylmalonic acids are performed to reduce false positive rates. The Laboratory participates in the Collaborative Laboratory Integrated Reports database for population comparison and cutoff adjustments. The Laboratory is accredited by the College of American Pathologists. In 2018, a total of 22 confirmed positive screens were detected for amino acids and acylcarnitines. No false negative screens were identified and a total of 91 false positive screens were reported. Positive screens included: PKU (3), TYR II/III (1), HPRO (1), ASA (1), CUD (2), MCAD (2), 3MCC (5), GAII (1), MMA/PA (5) and B12 (1). The most frequent false positive screens included: PKU (5), TYR II/III (7), CIT (8), MSUD (7), NKHG (6), CUD (13), 3MCC (8), GAI (5), SCAD (6) and MMA/PA (3). Review of false positive screens showed greater reliance of interpretation on second tier results would significantly lower the false positive rate. Also, plasma amino acid analysis for all 7 suspected MSUD screens showed that the false positive screens were due to an increased hydroxyproline. A second tier screen that detects hydroxyproline on dried blood spot has the potential to further reduce the false positive rate. The Metabolic Laboratory in Qatar seeks ways to improve newborn screening by reliable detection of affected newborns and by reducing false positive screens.

### P143. Accreditation of Newborn Screening Centers in the Philippine Setting

JomentoCharityLisingJovyCanlasMargarita AzizaEscorealMaria TrudaPanolKarenJubanNoelPadillaCarmencitaTherrellBradfordWileyVeronicaNational Institutes of Health-University of the Philippines-Manila, Newborn Screening Reference Center, Manila, Philippines

Background: As stated in the Republic Act 9288 or the Newborn Screening Act of 2004, Newborn Screening Centers shall be established strategically and be duly accredited by the Department of Health. Currently, there are six NSCs in the Philippines. Each NSC is required to provide laboratory and follow-up services to all Newborn Screening Facilities (NSFs) within their service area. Before a NSC can begin formal operation, it is required to successfully undergo an initial accreditation. Once licensed, the NSC must undergo reaccreditation every three (3) years.

Objective: This presentation reviews the accreditation process among Newborn Screening Centers (NSCs) in the Philippines.

Method: The process of NSC Accreditation for the past 8 years was reviewed.

Results and Discussion: The accreditation process includes satisfactory completion of requirements outlined in a published DOH Checklist. The Philippine Performance Evaluation and Assessment Scheme (PPEAS) was developed to aid in determining readiness. Over time and with use, this tool and has been updated so that it remains current. PPEAS addresses in some detail the various components of the NBS system. Periodically, outside reviews by a team comprised of selected NSC members takes place to augment the 3-year formal review cycle and help prepare for the formal review. As part of the formal DOH review, foreign experts experienced in such accreditation reviews have been included as members of the accreditation team.

Accreditation reviews consist of two parts. The first involves service delivery, technical operations and information management, which includes the international experts. The second focuses on education and regulation, human resource, administration and financing linkages and facility management. All findings and recommendations are discussed with the NSC management and require compliance before issuance of an operating license. The accreditation process has facilitated harmonization of newborn screening in the Philippines.

### P144. Internal Quality Control for Newborn Screening by Tandem Mass Spectrometry

ChiangSzu-HuiFanMei-LingChenHsiao-JanKaoShu-MinWangLi-YunHoHui-ChenGengGuoxingFanXinShiauYu-Shih ShiauHsiaoKwang-JenPreventive Medicine Foundation, Executive office, Taipei, Taiwan

Background: Expanded newborn screening by tandem mass spectrometry (TMS) has been adopted for many newborn screening laboratories worldwide in past decade. Many analytes, including amino acids (AA) and acylcarnitines (AC), could be analyzed concurrently in a single test from dried blood spot sample. However, most TMS reagent kits do not provide the control sample for all the analytes intended to be determined. An internal quality control (QC) is developed to assist the screening laboratory carrying out routine daily quality assessment independent of reagent kit.

Materials & Methods: QC materials were obtained from spotting bloods spiked with different levels of target analytes, including 10 AA and 28 AC (C0–C26), on newborn sample collecting filter paper (Whatman 903) by a GMP manufacture (ISO 13485 certified). Three newborn screening centers, one used derivatized method, one used non-derivatized method, the other used both methods, have evaluated these QC samples. QC results were reported via TMS IQC MIS System online. Real time statistic and control chart were shown on web for QC assessment.

Results: The between QC samples variations were less than 10% (CV). After package opened, the QC samples were stable at 4 degree C for intended daily use in one week. Two levels (low and high) of QC samples were included in routine analytical runs in each of the newborn screening laboratories. More than 3,000 set of results have been reported since January 2018. The within laboratory variations (CV) for most items were

### P145. Comparison of the TSH and OHP Values Between the Initial Contaminated and the Repeat Acceptable NBS Samples

AbarquezConchitaSouthern Philippines Medical Center, Newborn Screening Center Mindanao, Davao City, Philippines

Background: A very important quality indicator of an effective newborn screening system is a good quality of dried blood spot sample. Poorly collected samples are labeled unsatisfactory and are not fit for testing because of risk of giving inaccurate results. One type of unsatisfactory specimen is a contaminated sample. There are many causes of contamination and newborn screening coordinators are encouraged to submit acceptable samples at all times.

Objective: This study aims compare the TSH and OHP values between the initial contaminated and the repeat acceptable newborn screening samples, to determine if contaminated samples will give erroneous laboratory NBS results for the metabolites for CH and CAH and to determine if there is a need to change the protocol in testing contaminated samples

Methods: All initial NBS samples that were received at the NSC Mindanao from October 1, 2013 to July 7, 2017 and that were labeled as having contaminated dried blood spot were tested for the six-test NBS disorders. Following the usual protocol of NSC Mindanao, babies with contaminated samples were actively recalled by the NSCM Follow up Nurse to ask for an urgent repeat collection. Repeat acceptable samples were tested for the 6-tests. Retrospectively, values of the TSH and OHP for each sample were compared to the values detected in the initial contaminated samples.

Results: From October 1, 2013 to July 10, 2017, there were 4,558 initial contaminated samples received at NSC Mindanao. Only 75% submitted a repeat acceptable sample. Overall results for TSH and OHP screening revealed that TSH and OHP values for both initial and repeat cards were positively skewed and showed significant difference on its mean values.

Conclusion: There were significant differences in the TSH and OHP values of contaminated and acceptable NBS samples leading to false positive NBS screening for CH and CAH.

M. Evaluation of NBS Programme

### P146. Expanded Newborn Screening in Chile (26 Conditions): Results of a Pilot Program

CornejoVeronicaValienteAlfCabelloJuan FranciscoLobosGabrielBerriosCarmen GloriaValdebenitoSusanaInta, University of Chile, Genetic and Metabolic Disease Lab, Santiago, Chile

Introduction: Since 1992, Chile implemented a national program of Newborn Screening (NBS) for Phenylketonuria (PKU) and Congenital Hypothyroidism (CH), preventing intellectual disability in more than 2500 people. In September 2017 INTA started the Pilot Program (PP) to screen 26 pathologies. The first stage of the PP was initiated at Hospital San Juan de Dios (HSJD), which runs the regional laboratory of the national PKU-CH program.

Objectives: To present the implementation and results of the PP of NBS for 26 pathologies.

Materials and methods: INTA trained medical and laboratory team of HSJD in preanalytical, analytical and postanalytical processes of the expanded NBS and analyzed samples of dried blood spots (DBS) of all the newborn at HSJD maternity. The applied techniques were: Tandem Mass Spectrometry (MS/MS), Fluorometry (FL) and Immunofluorometry (IFL). For FL and IFL commercial kits were used and for MS/MS “in house” non-derivatized technique was used.

Results: of total 4554 DBS analyzed, 3752 (82.4%) corresponded to newborn of term. Recall rate was 1.6% (95% for MS/MS reanalysis). Samples were taken at ẋ 1, 7±0, 6 days of life. Results reports were available at ẋ 8, 4±2, 2 days of life. of the total samples analyzed, 2/4.554 were positive. A case of a medium chain acyl-CoA dehydrogenase deficiency (MCAD) (dg: 29 days of age) and the second case a Glutaric Aciduria type-1 (dg: 23 days of age) could be stablished.

Conclusions: the excellent results obtained with the training of the staff of the HSJD, allowed a low recall rate and early diagnosis of 2 positive cases and allows us to assert that this applied system it can be reproduced all over the country. However, there are some challenges that must be considered within its expansion: the acquisition of adequate equipment capabilities, preparing human capital in these technologies and to modernize the registration system to implement the PP in a national scale.

### P147. Revival of a Newborn Screening Program in the People’s Democratic Republic of Laos

HoehnThomasBounnackSaysanasongkhamUniversity Hospital Düsseldorf, General Pediatrics, Neonatology and Pediatric Cardiology, Düsseldorf, Germany

Background: Neonatal screening programs have been established and are in use in most countries worldwide. Laos belongs to the few countries which still have not established any kind of newborn screening.

Description: Participants of an initial pilot project between 2008 and 2010 were the large maternity hospitals within the city of Vientiane (Mahosot Hospital [2500 deliveries], Sethathirath Hospital [2000 deliveries], Friendship Hospital [700 deliveries] and Mother & Child Health Hospital [3500 deliveries]). Samples were taken immediately prior to hospital discharge and once weekly air-shipped to a German screening laboratory.

Lesson learned: Altogether 11,362 samples of newborn infants have been examined. The rate of retests was above European average due to very early discharge policies in Laotian maternity hospitals. Confirmed cases of neonatal congenital diseases included two infants with hypothyroidism and one infant with congenital adrenal hyperplasia without salt-loss. All three infants received early therapy and are currently doing well.

Next steps: To re-establish newborn screening in a sustained manner following the demonstration of feasibility during the pilot project. Newborn screening for TSH will be initiated in early 2019 in all delivery hospitals in the urban area of Vientiane, capital of Laos. As opposed to the pilot project, measurements in the lab will take place within the country at the laboratory of the largest delivery hospital in Vientiane, the Mother & Newborn Hospital.

### P148. The Analysis of Unrecalled Suspicious Positive Items during the Neonatal Disease Screening from 2005 to 2017 in Shanxi Province

ZhangHongmeiShanxi Maternal and Child Health Care Hospital, Neonatal Disease Screening Center, Taiyuan, China

Aim Analysis the screening rate of neonatal diseases screening and the reasons why the suspected positive items were not recalled in Shanxi Province from 2005 to 2017. Moreover, in order to improve the recall rate, we would like to provide recommendations and measures.

Methods The screening rate and suspicious positive non-recall status of screening for neonatal diseases in Shanxi Province from 2005 to 2017 were analyzed. The level of nursing is an important step in improving the screening rate in neonatal disease screening. The main screening item are the function of thyroid gland and phenylketonuria.

Results The newborn disease screening center of Shanxi maternal and child health hospital (3+1 mode) screened a total of 2,325,785 newborns in Shanxi Province from 2005 to 2017. The screening rate increased from 5.0% in 2005 to 91.2% in 2017. The recall rate of phenylketonuria increased from 74.4% in 2005 to 92.7% in 2017. The recall rate of congenital hypothyroidism increased from 54.0% in 2005 to 89.1% in 2017. Because of the clearing system and fluctuation in the number of the staff members in 2015 and 2016,1100 cases of congenital hypothyroidism were detected. The detection rate was 1/2114. 667 cases of phenylketonuria were detected. The detection rate was 1/3486. The detection rate of both diseases was higher than the national average. (The average detection rate of PKU was 1/11144 and the average detection rate of CH was 1/3009).

Conclusion Due to parents and inadequate medical supervision from 2005 to 2017 the suspicious positive items were not recalled in Shanxi Province.

### P149. Quality Analysis of Neonatal Screening from 1989 to 2017 in Beijing

ZhaoJinqiYangHaiheYangNanGongLifeiKongYuanyuanBeijing Obstetrics and Gynecology Hospital, Capital Medical University, Beijing, China, Department of Newborn screening, Beijing, China

Objective To analyze the screening results of neonatal diseases in Beijing, and to explore the incidence and treatment of congenital hypothyroidism (CH) and phenylketonuria (PKU).

Methods In 1989-2017, radioimmunoassay (RIA) (1989–1995), ELISA (1996–2003), and time-resolved fluorescence immunoassay (DELFIA) (2004–2017) were used to detect thyroid stimulating hormone (TSH). content. The phenylalanine (Phe) content was determined by Guthrie Bacterial Suppression (BIA) (1992–2003) and Fluorescence (2004–2017).

Results A total of 3,507,643 newborns were screened from 1989 to 2017. The screening rate increased from 14.01% in 1989 to 100.13% in 2017. The positive rate of CH positive rose from 80.8% in 1989 to 96.7% in 2017. PKU positive recall rate from 54.2% in 1992 to 97.8% in 2016. A total of CH1472 cases were diagnosed, the incidence rate was 1:2383, 495 cases were PKU, and the incidence rate was 1:7086. In terms of treatment effect, the proportion of diagnosed time in the first half of the month of CH patients increased from 0 to more than 10%, showing an upward trend in 2012–2017. The ratio of the postnatal treatment time of children in the same period was 14–20 days (c2 = 4.391, *p* = 0.036) increased from 0 to 44.5%, and the proportion of 28–90 days (c2 = 4.139, *p* = 0.042) decreased from 83.3% to 22.6%. The follow-up effect of children with PKU was also improved but not statistically significant.

Conclusions Neonatal screening is not only a laboratory test, but a systematic multidisciplinary closed-loop service system. Each step in the system is closely related to the accuracy and reliability of screening results. Beijing has established a screening system for neonatal diseases. In the future, it will continue to conduct quality control on the screening of genetic metabolic diseases through screening networks, and use various quality indicators as indicators to promote the quality of screening for metabolic diseases in Beijing.

### P150. Where Are They Now? Long Term Follow up of Patients with Amino Acid Disorders Screened for in NSW (excluding phenylketonuria)

JunekZenaWileyVeronicaEllawayCarolynThe Children’s Hospital at Westmead, NSW Newborn Screening Program, Westmead, Australia

The NSW Newborn Screening Program has been screening for amino acidopathies and related disorders by tandem mass spectrometry, with varying reliability, since 1998. We have reviewed the progress of patients born over a ten-year period between January 2007 and December 2016 detected by newborn screening and treated for amino acid disorders, excluding those with phenylketonuria. Amino acid disorders included are: pterin defects, maple syrup urine disease, tyrosinemia type II, methionine adenosyl transferase deficiency, homocystinuria, carbamoyl phosphate synthase deficiency, citrullinemia type I and arginine succinate synthase deficiency.

Over the 10-year period, 1,030,000 babies were screened in the neonatal period using dried bloodspot samples. 45 children were detected through newborn screening and diagnosed with one of the amino acid disorders in this cohort. Patients labelled with the same disorder have been grouped together. A brief synopsis of the expected natural course of the untreated disorder is included for comparison. We reviewed treatment, anthropometrics and developmental progress noted over time when seen in the metabolic clinic.

The overall improvement in morbidity for affected individuals who were treated from the neonatal period compared to the expected course of the untreated disorder confirms our belief that newborn screening for these amino acid disorders is valid and worthwhile within our program.

### P151. Philippine PEAS: Over 15 Years of Experience in Program Monitoring and Evaluation

Asuncion PanolKarenConcordia SuarezRizaLisingJovy AnnBeltranFrederick DavidOrbilloLitaPadillaCarmencitaUniversity of the Philippines Manila, Newborn Screening Reference Center, National Institutes of Health, Manila, Philippines

Background. The Philippine Performance Evaluation and Assessment Scheme (PEAS) was developed to monitor quality and improvement of NBS program implementation at the level of laboratory testing centers or Newborn Screening Centers (NSCs), DOH Centers for Health Development (CHDs), collecting facilities or Newborn Screening Facilities (NSFs) and long-term follow-up clinics or Newborn Screening Continuity Clinics (NBSCCs). Philippine PEAS started in 2006 with tools developed to monitor the CHDs and NSFs. Monitoring tools for NSCs and NBSCCs were added in 2011 and 2018, respectively.

Objective. This presentation will present: 1) the PPEAS tools for the different stakeholders; 2) the critical issues, recommended policies/action identified in the monitoring process; and 3) the impact of these policies/actions.

Methodology. Reports spanning 12 years were reviewed. These include accomplished PPEAS tools; monitoring reports; minutes of meetings of the national advisory committee for NBS, the NBS technical working group and different consultative meetings; and DOH issuances. Indicators for assessment fall under the following components: operational structure, plan of action, systems in place, health promotion plan and contingency plan. For CHDs and NBSCCs, innovation strategies were also assessed.

Results and Discussion. Significant issues identified over the years in the implementation of the Philippine PEAS that led to policies include 1) expansion of the treatment and management referral network; 2) enhanced guidelines on the use of program funds; 3) NBS inclusion in social insurance; 4) establishment of G6PD Confirmatory Centers and NBSCCs; 5) formulation of the National Strategic Framework for ENBS, among others. Use of PPEAS in monitoring of NSCs, CHDs, NSFs and NBSCCs significantly contributed to the continuous improvement of the program. With the release of the Strategic Framework for ENBS, it is also timely and necessary to update the Philippine PEAS.

### P152. The Retrospect and Prospect of Neonatal Screening for Inherited Metabolic Disease in the City of Wuhan over two Decades

CaiWenqianHuXijiangShenShanshanWuhan Children’s Hospital (Wuhan Maternal and Child Healthcare Hospital), Tongji Medical College, Huazhong University of Science & Technology, Eugenic Genetics Laboratory, Wuhan, China

Objective: Neonatal Screening (NS) is widely recognized as one of the most valuable three-level preventive measures to prevent birth defects. Summarize the screening and diagnosis of neonatal genetic metabolic diseases in Wuhan in the past 23 years, improve the construction of NS platform and explore the development of NS.

Method: A retrospective analysis was carried out on the types, methods and platform construction of screening for neonatal diseases in Wuhan from 1996 to 2018.

Result: NS was conducted in Wuhan in 1996, including congenital hypothyroidism (CA) and hyperphenylalaninemia (HPA). In 1996–2002, CH and HPA screening was performed on newborns in the city by ELISA and BIA method. NS was performed by FEIA and fluorescence analysis from 2002 to 2015; the fully automatic new screening system detects the TSH and Phe content in the dried blood spots. Since 2016, Wuhan has increased the screening of erythrocyte glucose-6-phosphate dehydrogenase deficiency (G6PD, fluorescence assay) and congenital adrenal hyperplasia (CAH, time-resolved fluorescence) with a fully automated screening system and screened 42 genes using tandem mass spectrometry. NS covers 13 districts in Wuhan and the number of screenings has increased gradually year by year. So far, more than 1.2 million people have been screened. Screening positive children are recalled for diagnosis through a web platform covered Wuhan. The number of confirmed cases of CA and HPA was 721 (0.061%), 68 (0.006%) and that of G6PD and CAH was 26 (0.013%), 186 (0.093%).

Conclusion: After more than 20 years of exploration and development, Wuhan’s NS methods have made great progress and the platform has been gradually improved to reduce birth defects and improve birth quality. It has become the main means of tertiary prevention and has achieved good Social benefits.

**Keywords:** neonatal screening; genetic metabolic disease; incidence

### P153. Raising Awareness about Newborn Screening in the Philippines

MendozaVinaPadillaCarmencitaSuarezRiza ConcordiaReyesMa. ElouisaAlmazanAnthony JamesAguirreRosemarieUniversity of the Philippines Manila, Newborn Screening Reference Center, National Institutes of Health, Manila, Philippines

Background. A year before the passage of Newborn Act of 2004, the law that requires newborn screening to be offered in the Philippines, newborn screening uptake was low at 3.9% of the approximately 1.7 million births in 2003. With the implementation of the law, intensive efforts were undertaken to increase coverage. In the past several years, uptake has now reached 76.9%. Education is a key part of the newborn screening system and has played a major role in increasing screening coverage.

Purpose: This paper will 1) present the various educational tools used by the program and 2) review the successful strategies in raising awareness.

Methods and Results: The Department of Health (DOH) and the Newborn Screening Reference Center (NSRC) targeted various audiences considered to be key stakeholders in the screening process (parents, health professionals and policy makers) with an overtone of urgency in each activity. Differing forms of media have been used—audio/video (television, cinema, radio), written (newspaper and magazine articles, standardized written materials, calendars), advertisements (malls, jeepneys, tricycles) and popular social media and digital platforms. NBS information was also relayed to mothers at or near the time of birth.

Discussion. DOH and NSRC continually explore options to create a more reliable and consistent stream of educational activities and promotions as it implements the program in 7100+ hospitals and birthing centers. NSRC is now developing fifteen (15) series 20-minute segments to be shown at the lobbies and outpatient units of hospitals and maternity hospitals. The national target is at least 95% all Philippine births receiving expanded newborn screening by 2030.

### P154. Analysis of 1.26 Million Newborns’ Disease Screening in Guangxi

XuYuqiFanXinLinCaijuanHuangXiaotaoLiWeiMaternal and Child Health Hospital of Guangxi, Laboratory of Genetics and Metabolism, Nanning, China

Objective: To summarize the screening situation and diagnosis of neonatal Congenital hypothyroidism (CH), Phenylketonuria (PKU), G6PD deficiency and Congenital adrenal hyperplasia(CAH) in Guangxi Zhuang during 2013 to 2018.

Methods: All the subjects from 2013 to 2018 were collected heel blood after 72 hours of birth and 6 times of full lactation. Blood Phe and G6PD enzyme activity were determined by fluorescence assay, TSH and 17-OH levels were determined by time-resolved immunofluorescence assay. In addition, the treatment follow-up files were established for all confirmed children.

Results: 1,209,143 neonates were screened within the jurisdiction of Guangxi Screening Center during 2013 to 2018, taking up 95.43% for two items of new screening routine of the total amount. There were 18040 suspected positive cases(1.49%) of CH screening, finally, 570 cases were confirmed, at the same time 1631 cases of high TSH were diagnosed, and the prevalence rates of the two groups were 1/2121 and 1/1139; The number of suspected positive cases screened by PKU was 405 with a suspected positive rate of 0.03% and 10 cases were finally confirmed with a prevalence rate of 1/120,914; 1626 of 432,651 cases were Positive in CAH screening, with the suspected positive rate was 0.38%, 23 cases were confirmed and the prevalence rate was 1/18,811; of 191,702 G6PD screening cases, 13,718 were suspected positive, with a suspected positive rate of 7.16%.

Conclusion: The detection rate of CH and G6PD deficiency in Guangxi is relatively high, and the detection rate of CAH is close to the national level but the detection rate of PKU is relatively low. By combining tandem mass spectrometry, dozens of genetic metabolic diseases including amino acids, organic acids and fatty acids were detected. Therefore, early treatment for children can not only effectively control the harm of families but also improve the quality of the birth population.

**Keywords:** neonatal disease screening; G6PD; CH; CAH; PKU

### P155. Cost Benefit Analysis of Newborn Mass Screening for Phenylketonuria and Congenital Hypothyroidism in Korea

LeeDong-HwanLeeJeonghoSoonchunhyang University Hospital, Pediatrics, Seoul, Korea

The phenylketonuria (PKU) and congenital hypothyroidism (CH) are major disorders of inborn error of metabolism causing mental retardation if proper therapy is not promptly initiated. But these serious problems can be prevented by early diagnosis and treatment on neonatal test and early intervention for all newborn infant and medical expenses for treatment and institutionalization of already clinically developed patients.

1. The estimated incidence of the PKU and CH were 1/71,250 and 1/3817, respectively, in 213,749 newborns examined mass screening for inborn error of metabolism in Korea.
2. The cost for neonatal screening test for PKU and CH per one newborn is ￦8000, thus the total cost for screening test for all newborn babies for 1 year (650,000 newborn babies/year) will be ￦5.2 billion.
3. The cost for early intervention of each patient detected by neonatal screening test is ￦106.5 million for one PKU patient and ￦13.62 million for one CH patient. Annual total cost for neonatal screening test and early intervention of detected cases amount to ￦8.474 billion.
4. The cost for treatment and institutionalization of already developed patients are ￦1.62 billion for self-care and ￦ 62.51 billion for institutionalization, thus total expenses amount to ￦64.13 billion.

In conclusion, annual costs for neonatal screening test and intervention of early detected cases are less expensive than the expenses for treatment and institutionalization for already developed patient due to delayed diagnosis (￦8.747 billion versus ￦64.13 billion), so a cost-benefit rate is 1:7.4. Therefore, neonatal mass screening test for PKU and CH are necessary and economically beneficial for individual patient as well as national finances.

### P156. 10-Year Analysis of Inherited Metabolic Diseases Diagnosed with Tandem Mass Screening Test in Korea

LeeJeonghoLeeDong-HwanSoonchunhyang University Hospital, Pediatrics, Seoul, Korea

Purpose: From the early 1990’s, use of Tandem Mass spectrometry in neonatal screening test, made early stage detection of disorders that was not detectable by the previous methods of inspection. This research aims to evaluate the frequency of positive results in national neonatal screening test by tandem mass spectrometry and its usefulness.

Methods: A designated organization for inherited metabolic disorder executed neonatal screening test on newborns using tandem mass spectrometry followed by the investigation of these data by the Planned Population Federation of Korea (PPFK).

Results: There were 4,590,908 total newborns with 3,445,238 of them tested and showed 120 cases of amino acid metabolism abnormality, 31 cases of fatty acid metabolism abnormality and 110 total cases of organic acid metabolism abnormality. Within amino acid metabolism abnormality, most of them were due to: 30 cases of Citrullineuria Type 1, 25 cases of Tyrosinemia and 23 cases of Phenylketonuria. For fatty acid metabolism abnormality, most were related to 10 cases of Carnitine Uptake Deficiency, 9 cases of Very Long Chain Acyl-CoA Dehydrogenase Deficiency and 7 cases of Medium Chain Acyl-CoA Dehydrogenase Deficiency. For organic acid metabolism abnormality, 25 cases of Isovaleric acidemia, 24 cases of 3-Methylcrotonyl-CoA Carboxylase Deficiency and 13 Methylmalonic Acidemia was highly shown.

Conclusion: Until now, it was difficult to precisely grasp an understanding on the national incidence of inherited metabolic disorder, due to lack of overall data and inconsistent and incomplete long-term result analysis. However, this research attempted to comprehensively approach the domestic incidence, by analyzing previous 10 years of data. In future, it seems a system is required to accurately understand and diagnosis prevalence rate, by efficiently realizing re-diagnosis and definite diagnosis test if the results of neonatal screening test is to be positive.

### P157. Expanded Newborn Screening for Inborn Error of Metabolism Using Tandem Mass Spectrometry in Thailand

LiammongkolkulS.^1^SanomchamK.^2^SathienkijkanchaiA.^2^VatanavicharnN.^2^WasantP.^2^1Faculty of Medicine, Siriraj Hospital, Pediatrics, Bangkok, Thailand2Div. of Medical Genetics, Dep. of Pediatrics, Faculty of Medicine, Mahidol University, Thailand

Introduction: At present, only congenital hypothyroidism (CH) and phenylketonuria (PKU) are included in the national newborn screening in Thailand. A pilot expanded newborn screening program to detect other inborn errors of metabolism (IEM) using tandem mass spectrometry (TMS) was implemented at Siriraj Hospital, Bangkok in May 2014 with funding support from National Health Security office (NSHO) Bangkok Region.

Methods: Between May 2014 and December 2018, dried blood spots (DBS) from 164,855 neonates sent from 17 public hospitals in Bangkok were screened for inborn errors of amino acids, organic acids and fatty acids metabolisms were collected on days 3 of life in well newborns. In addition, the samples were collected on days 14 and 28 of life in preterm or sick newborns. Samples were analyzed for amino acids and acylcarnitine profiles using TMS by PerkinElmer’s Neobase non-derivatized MSMS kit. Samples with abnormal results were repeated and the babies were recalled to confirm diagnosis with plasma amino acids, plasma acylcarnitines and urine organic acids.

Results: A total of 17 newborns and 11 mothers were confirmed to have inborn errors of metabolism, including 5 primary carnitine deficiency, 1 citrullinemia, 1 MMA; 2 MSUD, 2 GA I, 1 GA II, 2 IVA, 1 PA, 1 VLCAD deficiency, 1 CPT I deficiency, 8 maternal primary carnitine deficiency, 2 maternal beta-ketothiolase deficiency and 1 maternal GA I. The overall incidence of IEM in Bangkok identified by expanded newborn screening was approximately 1:9,697 live births or 1: 5,888 including mothers. Most of these babies received treatment much earlier than ones without receiving newborn screening which led to prevention of death and disability and significant reduction of hospital admission rates.

Conclusion: Expanded NBS could early detect several neonates affected with IEM in Bangkok. NHSO is considering expansion of neonate metabolic screening to other provincial areas of Thailand in the future.

### P158. An Assessment on the Performance of the Different Courier Service Providers in the Mindanao Newborn Screening Program

GuilaranSheila MaeAbarquezConchitaNewborn Screening Center Mindanao, Program Development, Davao City, Philippines

Background: The effectiveness of the newborn screening system depends on the timely collection and submission of the samples for early detection and management. In the Philippines, the newborn screening program depends heavily on the courier delivery system for transmittal of NBS samples from collecting facility to NBS centers. Poor courier performance may be costly to the facility and detrimental to patients.

Objective: The study aims to assess the performance of the different courier service providers in Mindanao in terms of timely delivery and acceptance of the samples delivered, to identify factors that affect delays in delivery and to determine if facilities were able to perform repeat sample collection for unsatisfactory samples caused by courier errors.

Methods: A two-year courier data was retrieved from the Neometrics database, corrective and preventive action request forms filed by NSC Mindanao staff and monthly reports of incoming pouches received at the Center. Relevant data included actual delivery time (days) of pouches, number of pouches delivered by each courier service provider and the number of courier errors reported.

Results: Form 2017–2018, a total of 163,731 pouches was received at NSC Mindanao from the different courier service providers. Majority (83%) of the samples were transmitted through JRS courier. On the average, the courier service providers were able to deliver the NBS pouches to NSC Mindanao in 4 days. A total of 24 courier errors were reported within the two year period. Seventy four NBS samples needed repeat collection as a result of the courier errors but only 41 (55.4%) of the babies came back for a repeat newborn screening test.

Conclusion: Courier services play a crucial role in the successful implementation of the newborn screening program. Newborn screening centers should work closely with all courier services in achieving the goal of saving babies’ lives. Both stakeholders should implement measures to prevent recurring errors in the transmittal of NBS samples.

### P159. Newborn Screening Program in Saudi Arabia: An Overview

AlodaibAliAlAhaidibLujane Y.SaadallahAmalKFSHRC, Genetics, Riyadh, Saudi Arabia

The Saudi Newborn screening is a public health Program that prevents serious complications of a selection of 17 metabolic and endocrine inherited disorders when detected shortly after birth in their asymptomatic stage. The program was kicked off in August 2005. The aims of this review are, to present estimates of the prevalence of all screened disorders in the country, in addition to highlight on some of the epidemiological and quality characteristics of detected cases.

It is anticipated that the results of this retrospective cross-sectional review of data obtained from the Newborn Screening program at King Salman Center for Disability Research and King Faisal Specialist Hospital and Research Center will help in detecting trends and prevalence of these screened disorders in the kingdom. Also, it will add evidence about the magnitude of the screened disorders and magnify the need toward the establishment of a nationwide registry system for all newborn screened disorders. Also, it might tempt authorities to move towards preventive health actions as carrier genetic testing of the newborn screened conditions to the currently existing Saudi Premarital Screening Program.

### P160. Response Time of Newborn Screening Coordinators to Late Newborn Screening Samples

BermudezPerlyAbarquezConchitaNewborn Screening Center Mindanao, Program Development, Davao City, Philippines

Background: One of the many hurdles in the implementation of newborn screening is the poor quality of specimen submitted to the Center. Unsatisfactory specimens are dried blood spots on filter cards that are not fit for testing because of improper collection or transport. Dried blood spot samples which are received at the center more than 14 days from date of collection are considered late samples and cards of this type are considered unsatisfactory specimen.

Objective: This study aims to assess the response time of NBS coordinators to late NBS samples, to identify the causes of late transmittal of samples and to identify the effect of late transmittal of samples.

Methods: Between January 2014 and December 2018, a total of 1,608,758 newborn infants were screened by NSC Mindanao. A review of late NBS samples data from the Neometrics database and CAR forms of NSC Mindanao was done retrospectively. Relevant information gathered include total number of late samples and total number of return samples per region, causes of late transmittal and turnaround time of repeat samples.

Results: Out of the 1,608,758 initial samples received from 2014 to 2018, a total of 3,747 (0.23%) were labeled as late samples. Only 2,825 (75%) of these samples were repeated and gave an average screening age of 62 days for the affected newborns. Majority of the causes for late transmittal of NBS samples were due to errors by the NBS coordinators.

Conclusion: Unsatisfactory specimens are costly on many levels and are highly discouraged because these could give erroneous laboratory results. Furthermore, these types of specimen require a repeat collection causing delays in the screening and management of affected cases. Unrecalled unsatisfactory specimen represents no screening.

### P161. Newborn Screening Technical Assistance Evaluation Program (NewSTEPs): A Model Resource Center for Us Newborn Screening Programs

SinghSikhaOjoduJeliliAssociation of Public Health Laboratories, Newborn Screening and Genetics, Silver Spring, Washington, DC, USA

Measurable Objective: To describe how NewSTEPs, the nationally funded resource center in the United States, ensures that US newborn screening (NBS) programs can use data and implement quality improvement practices to evaluate, analyze and benchmark their performance.

Methods: Reporting mechanisms for three tiers of data entered by NBS programs are made available to stakeholders. Public state profiles share high level demographic, policy, program and disorder related information.

Results: NewSTEPs offers a voluntary data repository with uniform data elements.

State NBS programs serve newborn populations ranging in size from 6100 births per year to over 500,000 (median 52,200). Regional laboratories serve 13 states, while all states have their own short term follow-up programs. Funding of NBS activities is secured from different sources, however most programs (*n* = 47/51) have an NBS fee (median $71, range $15–157). Storing residual dried blood spots ranges from 1 month to more than 27 years, with a median of 1 year. New disorders are added to state NBS panels via a variety of mechanisms (legislation *n* = 22, commissioner/board of health *n* = 18, other *n* = 2, unknown *n* = 9).

Conclusion: NewSTEPs is the central source for data on NBS programs and supports quality improvement practices. The website contains reports, maps and infographics. The NewSTEPs repository and technical assistance resource center serves as a model for collecting, analyzing and sharing data in support quality improvement within a national public health surveillance program. This model can be extrapolated to serve other, similar surveillance activities.

NewSTEPs is funded through Cooperative Agreement # U22MC24078 HRSA/MCH.

### P162. Inborn Errors of Metabolism Detectable by Tandem Mass Spectrometry in Beijing

ZhaoJinqiGongLifeiYangHaiheKongYuanyuanBeijing Obstetrics and Gynecology Hospital, Beijing Maternal and Child Health Care Hospital, Capital Medical University, Department of Newborn Screening, Beijing, China

Background and objectives Individual inborn errors of metabolism (IEM) are rare disorders but may not be that uncommon in our patient population. An early diagnosis allows presymptomatic treatment which can prevent severe permanent sequelae and in some cases death. Expanded newborn screening for IEMs by tandem mass spectrometry (MS/MS) is an efficient approach for early diagnosis. Here we report the application of this approach to the identification of newborns in Beijing at risk of developing a potentially fatal disease between July 2014 and February 2019.

Methods The amino acids and acylcarnitines in dried blood spots were analyzed by tandem mass spectrometry. Diagnoses of newborns with elevated metabolites were confirmed by gas chromatography-mass spectrometry, biochemical studies and genetic analysis.

Results Among the healthy newborns, 14 metabolic disorder cases were confirmed, giving a total birth prevalence of 1:4100 live births. OA were the most common (6/14 patients; 43%) and methylmalonic aciduria was the most frequently observed OA (5/6 patients; 83%). Four infants were diagnosed with methylmalonic acidemia with homocystinuria type CblC, 1 with isolated methylmalonic academia and 1 with isovaleric academia. HPA were diagnosed in 4/14 patients (29%). One was diagnosed with medium-chain acyl CoA dehydrogenase deficiency, 1 with carnitine uptake deficiency, 1 with maple syrup urine disease and 1 with citrin deficiency. Eight cases underwent genetic analysis. Thirteen mutations in six IEMs-associated genes were identified in 8 confirmed cases.

Conclusion We believe that our data underestimate the true incidence of IEM in the region. Regional newborn screening programs will provide a better estimation of the incidence of IEM. We recommend a centralized newborn screening program that employs tandem mass spectrometry.

**Keywords:** inborn errors of metabolism; tandem mass spectrometry; newborn screening

### P163. Routine Newborn Screening of Newborns—Why can ISNS & other International organizations not make it mandatory?

KumarKishoreNagarNandiniS.V.GirishCloudnine Hospitals, India, Neonatology, Bangalore, India

Study Objectives: Routine newborn screening program for various disorders was started mandatorily in our group of hospitals 11 years ago for 2 reasons: World over, there is enough evidence of Newborn Screening Saving Lives (NSSL) and to see if it is applicable to Indian subcontinent?

Methods: Screening for various disorders including congenital hypothyroidism (CHT), Glucose-6-Phosphatase Dehydrogenase Deficiency (G6PD), Congenital Adrenal Hyperplasia (CAH), Galactosaemia (GLT), Biotinidase deficiency (BD), metabolic disorders along with screening for disorders like Developmental Dysplasia of Hips (DDH), Retinopathy of Prematurity (ROP), Newborn Hearing Screening (NHS), Screening for Critical Cyanotic Congenital Heart Disease (CCCHD) was performed on all babies born from various hospitals in the group in South India who consented to be part of this between 19th January 2007 and 19th January 2018.

Results: In the study period, a total of 69,426 babies were screened. There were 86 cases of CHT (incidence of 1:807), 1735 cases of G6PD (incidence of nearly 2.5%), 27 cases of CAH (incidence of 1:2,500), 2 cases of Galactosaemia (incidence of 1: 34,713), 1 case of Biotinidase deficiency (incidence of 69,426), many metabolic disorders – almost 245 cases—0.35% incidence - predominant among them being Urea Cycle Defects (UCD—10 cases of Citrullinemia—incidence of 1:6,942), 4 cases of deafness (amounting for 1:17,356 cases), 13 cases of DDH (incidence of 1:5340) and 24 cases of CCCHD (incidence of 1:2,900), 4 cases of ROP (incidence of 1:17,350)—suggesting that there is enough proof in the pudding to enforce mandatory screening in all member countries.

Conclusions: (1) ISNS has a responsibility for all members & countries with its knowledge~base. (2) Why can ISNS not issue a statement asking member countries---which can save lives and help decrease morbidity and mortality (and reduce IMR in member countries).

**Keywords:** newborn screening; newborn screening saves lives (NSSL)

### P164. Results from a Pilot Study Evaluating 26 Additional Target Disorders for the German Newborn Screening Panel

GramerGwendolynFang-HoffmannJunminFeyhPatrikKlinkeGlynisMonostoriPeterOkunJürgen G.HoffmannGeorg F.University Hospital Heidelberg, Centre for Pediatric and Adolescent Medicine, Division for Neuropediatric and Metabolic Medicine, Heidelberg, Germany

Background: Until March 2018 newborn screening in Germany included 15 target disorders. Diagnostic improvements suggest a further extension of the panel.

Methods: Since August 2016 a prospective study evaluating 26 additional target disorders (25 metabolic disorders and vitamin B12 deficiency) is performed at the Newborn Screening Centre Heidelberg, using second-tier strategies for 15 of the additional target disorders.

Results: From August 2016–December 2018 190,589 children participated in the study. Second-tier analyses were performed in 7% of samples. The recall rate was 0.1% for the additional target disorders. Target disorders from the study panel were confirmed in 58 children: 1 HMG-CoA-lyase deficiency, 1 citrullinemia type I, 3 MAD deficiency, 2 MTHFR deficiency, 2 propionic aciduria, 5 OTC deficiency, 1 carnitine transporter deficiency, 1 tyrosinemia type I, 1 maleylacetoacetate isomerase deficiency and 42 children with maternal vitamin B12 deficiency (1 with MAD deficiency + vitamin B12 deficiency). All mothers of children with vitamin B12 deficiency were offered standardized work-up for vitamin B12 deficiency and were referred to internal medicine for further diagnostics and treatment if indicated.

At diagnosis 52 patients were asymptomatic, 6 symptomatic (5 OTC deficiency, 1 severe MAD deficiency). 3 of 6 children diagnosed symptomatically deceased despite early treatment (2 OTC deficiency, 1 severe MAD deficiency).

Conclusion: Within 29 months the study “Newborn Screening 2020” identified additional 58 children with potentially treatable conditions while only marginally increasing the recall rate. Most children were diagnosed pre-symptomatically. Maternal vitamin B12 deficiency was the most frequent finding with 1 in 4500 newborns. Even more children could benefit from screening for the additional target disorders in case of a future comprehensive extension of the German and other newborn screening panels.

Funding: Dietmar Hopp Foundation, St. Leon–Rot, Germany.

### P165. Newborn Screening Program by Tandem Mass Spectrometry for Inborn Metabolic Disease in South China: Incidence, Genetic Characteristics and Clinical Outcome

JiaZhengjunLiuJingZhangYanghuiTangHuaXiHuiChenJingFangJunqunXieDonghuaWangHuaMaternal and Child Health Hospital of Hunan Province, Department of Genomic Medicine, Newborn Screening Center of Hunan Province, Changsha, China

Objective This study sought to investigate the prevalence, mutation characteristics and clinical outcomes of inborn metabolic disorders (IMDs), detected by tandem mass spectrometry (MS/MS) newborn screening in Hunan province, south of China.

Method We studied 565,182 newborns who underwent MS/MS screening for IMDs, including fatty acid oxidation disorders (FAODs), amino acid disorders (AAs) and organic acidemias (OAs) between March 2013 and September 2017. For patients with positive results, recall screening test was performed and the results were further confirmed by specific biochemical and genetic analysis. For all patients with IMD, guideline-directed medical treatment was administrated and the follow-up outcomes were evaluated.

Result A total of 107 newborns were diagnosed with IMDs, with an overall prevalence of 1:5282, including 65 newborns with FAODs (1: 8695), 29 with AAs (1:19,489) and 13 with OAs (1:43,476). The Primary carnitine deficiency (1:12,845), Hyperphenylalaninemia (1:33,246), Short chain acyl-CoA dehydrogenase deficiency (1:47,099), Citrine deficiency (1:94,197) were the four most common IMDs. The hotspot mutation in SLC22A5 gene of PCD were c.51C>G (25.3%), c.1400C>G (23.0%) and c.760C>T(13.8%); in PAH gene of HPA were c.728G>A (22.2%) and c.721C>T(14.8%); in ACADS gene of SCAD was c.1031A>G (38.9%); and in SLC25A13 gene of NICCD was c.851_854delGTAT (50.0%). During a mean follow-up of 26.1 ± 5.6 months, 7 patients were died, 4 were suffered intelligent disability, whereas the remaining 96 subjects had normal physical and intelligent development.

Conclusion The overall prevalence of IMDs is not fairly low in south of China. The spectrum of IMDs presents with typical regional characteristics and the hotspot mutation profile is consistent with that detected in Chinese population. Newborn screening by MS/MS enables the early detection of IMDs. Early appropriate management can significantly improve the outcomes of these patients.

### P166. The Results of 1.1 Million Newborns Screening by Tandem Mass Spectrometry in Shanghai China

HanLianshuYeJunGuXuefanXinhua Hospital, Shanghai Institute for Pediatric Research, Shanghai Jiaotong University School of Shanghai, China

Objective The aims of this study were to determine the prevalence of amino acids disorders, organic acidemias and fatty acid oxidation disorders in Shanghai China by newborn screening using tandem mass spectrometry (MS/MS).

Methods 1.1 million neonates were collected and screened by MS/MS from January 2003 to December 2018 in Shanghai China. The Patients with confirmed diagnosis were treatment and follow-up.

Results In total, 315 cases were diagnosed with 21 kinds of diseases (1/3507). 163 cases(51.7%) were diagnosed with 9 kinds of amino acids disorders, include 126 with hyperphenylalaninemia, 13 with hypermethioninemia, 8 citrin deficiency, 7 with maple syrup urine disease, 2 with citrullinemia type I, 2 with tyrosinemia type II, 2 with homocystinuria, 2 with Arginemia and 1 with argininosuccinic aciduira); 85 cases (27.0%) were diagnosed with 7 kinds of organic acidemias, include 38 with methylmalonic academia, 26 with 3-cethyl crotonyl-CoA carboxylase deficiency, 8 with propionic acidemia, 7 with isovaleric academia, 3 with glutaric acidemia type I, 2 with isobutyryl-CoA dehydrogenase deficiency and 1 with 3-hydroxy-3-methylglutaryl-CoA lyase deficiency); 67 cases (21.3%) were diagnosed with 5 kinds of fatty acid disorders (32 with primary carnitine deficiency, 21 with short chain acyl-CoA dehydrogenase deficiency, 11 with medium chain acyl-CoA dehydrogenase deficiency, 2 with multiple acyl-CoA dehydrogenase deficiency and 1 with very long chain acyl-CoA dehydrogenase deficiency).

Conclusion Newborn screening with MS/MS was valuable in early diagnosis of severe and treatable amino acid diseases, organic acidemias and fatty acid oxidation disorders. Hyperphenylalaninemia, methylmalonic acidemia and primary carnitine deficiency are common diseases in Shanghai China

### P167. 14 Years’ Experience in Expand Newborn Screening Using MSMS and Confirmation Positive Results in KFSHRC Riyadh, Saudi Arabia

AlamoodiMohamed SKFSHRC, Genetics, Riyadh, Saudi Arabia

Newborn screening is a public health program of screening in infants shortly after birth for a list of conditions that are treatable but not clinically evident in the newborn period. In 1994, metabolic screening lab in KFSHRC introduced expanded newborn screening for amino acids oxidation, amino acids disorders and organic acids acidemia. The talk will include the following

1. Newborn Screening in Saudi Arabia
2. Sampling/from collection from the baby to receive in the lab
3. Samples process/from revived in the lab to final steps before injection
4. Injection/program the system
5. Interpretation/data for normal and positive
6. MSMS operation/brief explanation about the system
7. Biochemical confirmation for positive results/example of OA results
8. Conclusion

### P168. Overview of Newborn Screening Program of Bionet Vietnam Newborn Screening Center

QuocHai LuyenThanhHoan Vu ThiThiHoai NguyenThuMai Do ThiThiHue PhamBionet Viet Nam Newborn Screening Center, Newborn Screening, Ha Noi, Vietnam

Bionet Newborn Screening (NBS) Center, a private entity located in Vietnam, developed a Newborn screening program in 2012. Bionet NBS Center instituted the NBS process with the initial intent of i) expanding the screening panel. In 2012, Bionet NBS Center implemented screening for five basic conditions included in the NBS panel: G6PD, PKU, GAL, CH and CAH. Expanded panels also began which included: hemoglobinopathies using VARIANTTMnbs Sickle cell program (Bio-Rad) and 60 inherited metabolic disorders using Shimadzu’s LC-MS/MS 8040 system. In order to ii) expand geographic areas and increase the rate of children screened, Bionet NBS Center cooperates with more than 200 medical units from 45 provinces. This amounts to the testing of approximately 100,000 babies each year. In order to perform these tests and communicate results, Bionet NBS Center iii) provides an effective, safe and professional blood collection kit for NBS specimens and iv) deploys a comprehensive process from genetic counseling and screening to diagnostic testing, treatment, follow- up and management. Bionet NBS center applies a dual procedure for NBS to minimize false positive cases. After the initial screen, all positive specimens are followed up closely by biochemical diagnostic tests such as: quantitative assay of organic acids in urine by GC/MS, quantitative testing of enzyme activity or intensive diagnosis by molecular techniques including identification of G6PD gene mutations or thalassemia gene mutations. In addition to screening, Bionet NBS Center cooperates with doctors at Children’s hospitals to support the treatment method quickly and accurately. To manage all data associated with the NBS program, Bionet NBS Center has developed an exclusive software which allows for quick and accurate reporting. With clear orientation and objectives, in 2019, Bionet NBS Center will continue to update and deploy the newest technology in NBS to develop a more comprehensive NBS program in Vietnam.

### P169. Utilizing a National Data System for Newborn Screening Improvement

ReyesMa ElouisaPadillaCarmencitaSuarezRiza ConcordiaJubanNoelUniversity of The Philippine Manila, Newborn Screening Reference Center, National Institutes of Health, Manila, Philippines

Introduction The National Data System for Newborn Screening is comprised of data coming from different aspects of program implementation. Newborn Screening in the Philippines has screened more than 12 million babies since 1996. Records of screened babies are kept and stored in newborn screening laboratories across the country utilizing one laboratory information management system. The system records both laboratory data and follow-up/case management related information. Pertinent data are submitted to and consolidated by Newborn Screening Reference Center, the technical arm of the Department of Health. Aside from data coming from the newborn screening laboratories, the program has generated data from other tools used in various program implementation activities including the Philippine Performance Evaluation and Assessment Survey tool and record of evidences attached to the tool. With the establishment of the long-term follow-up program in 2014, the Continuity Clinic Online Record System was put in place to record compliance to treatment and management.

Purpose This paper reports on 1) how data were and are currently being utilized by the program and 2) some recommendation to further improve the system for use of other health programs.

Materials and Methods A review of all program activities both in local and national that utilized data coming from the NBS Data System was done.

Results Results show that NBS data were utilized in 1) gap analysis and measurement of national key performance indicators that is, coverage, quality of samples, timings, patient outcomes, 2) aid in laboratory operations that is, dealing with unsatisfactory samples, determining personnel workloads, case audits, 3) fulfilling national mandates that is, establishing protocols, review of algorithms, 4) improvement of implementation at the regional level that is, training, monitoring and evaluation plans, 5) in aid of policy, 6) researches, 7) establishment of referral centers that is, confirmatory centers, follow-up/treatment network.

### P170. Screening of Inherited Metabolic Diseases in 20,307 Li Nationality Newborns

WangJieHainan Maternal and Child Health Hospital, Neonatal Disease Screening Center, Haikou, China

Objective: To investigate the incidence of multiple genetic metabolic diseases in Li nationality in Hainan Province.

Methods: A total of 20,307 samples of dried blood samples from Li nationality newborns born in Hainan Province from October 1, 2016 to September 31, 2016 were collected. The non-derivatized multi-amino acid, carnitine and succinylacetone assay kits produced by PE were used. (tandem mass spectrometry) for tandem mass spectrometry screening, the initial screening indicators abnormal call recall and still abnormal blood samples to Beijing Maikeno Gene Company for genetic diagnosis.

Results: There were 840 cases of abnormal primary screening index among 20307 newborns and the initial screening abnormal rate was 4.14% (840/20,307). Five patients were followed up and 835 patients were recalled. After review, 770 patients were excluded, 65 patients were suspected of having IEM and 12 patients with IEM were diagnosed by genetic diagnosis. Among them: 5 cases of primary carnitine deficiency, 3 cases of phenylketonuria, 2 cases of 3-methyl crotonyl-CoA carboxylase deficiency, 1 case of methylmalonic acidemia, malonateemia 1 example. The incidence of multiple inherited metabolic diseases in this minority area of Hainan Province is 59/100,000 (13/20,307).

Conclusion: The incidence of multiple genetic metabolic diseases is high in the Li ethnic group in Hainan Province, especially the highest incidence of primary carnitine deficiency. Screening of multiple genetic metabolic diseases by tandem mass spectrometry is of great significance.

**Keywords:** Li; newborn; genetic metabolic disease; tandem mass spectrometry;

### P171. Provincial Distribution of Cases Detected Through Newborn Screening in The Philippines

ReyesMa ElouisaPadillaCarmencitaJubanNoelUniversity of The Philippine Manila, Newborn Screening Reference Center, National Institutes of Health, Manila, Philippines

Introduction: The Philippines is an archipelago of more than 7000 islands grouped in 3 major islands. It is further politically subdivided into 84 provinces clustered into 17 regions. It also has 175 ethnolinguistic groups. Newborn Screening (NBS) in the Philippines started in 1996. In its 22 years of implementation, it has screened 12,233,971 newborns, yielding 4,335 positive cases for CH, 614 for CAH, 91 for PKU, -----131 for GAL, 196,160 for G6PD Deficiency. In 2012, MSUD was included in the panel, since then picking up 122 affected. Expanded NBS was started in December 2014 and of 665,578 screened, 16 cases of fatty acid disorders, 16 cases of organic acid disorders, 4 cases of urea cycle defects 480 cases with hemoglobin disorders were identified. This aggregated data is currently the basis for strategies employed in the implementation of the different components of the newborn screening program.

Purpose: This paper aims to 1) show the distribution of detected cases in the newborn screening panel since 2005 at the regional and provincial level, 2) establish if the distribution of disorders in the Philippines is concentrated only in specific areas, 3) determine usability of results in creating strategies to improve some aspects of the program implementation.

Methods: Review of data submitted by the different follow-up programs and use of mapping tool for graphical presentation.

Result: The study shows that though almost all 84 provinces in the country has cases of CH. Distribution of MSUD cases seemed to support a study on MSUD in the Philippines [Silao, 2004] identifying a founder mutation with a carrier frequency of one in 100 Filipinos. Most MSUD cases are found in adjacent provinces of Metro Manila, Region 3 and Region 4, with most cases found in Pampanga. Patients with CAH are concentrated in Metro Manila, Pampanga, Bulacan, Pangasinan, Cagayan and Cebu. The results can aid different program stakeholders in planning or creating strategies, for example, setting up of continuity clinics and health profiling.

